# A chemical tool for blue light-inducible proximity photo-crosslinking in live cells†

**DOI:** 10.1039/d1sc04871f

**Published:** 2021-12-10

**Authors:** Pratyush Kumar Mishra, Myeong-Gyun Kang, Hakbong Lee, Seungjoon Kim, Subin Choi, Nirmali Sharma, Cheol-Min Park, Jaewon Ko, Changwook Lee, Jeong Kon Seo, Hyun-Woo Rhee

**Affiliations:** Department of Chemistry, Seoul National University Seoul 08826 Korea rheehw@snu.ac.kr; Department of Chemistry, Ulsan National Institute of Science and Technology (UNIST) Ulsan 44191 Korea; Department of Biological Sciences, Ulsan National Institute of Science and Technology (UNIST) Ulsan 44919 Korea changwook@unist.ac.kr; Department of Brain and Cognitive Sciences, Daegu Gyeongbuk Institute of Science and Technology (DGIST) Daegu 42988 Korea; UNIST Central Research Facilities (UCRF), Ulsan National Institute of Science and Technology (UNIST) Ulsan 44919 Korea jkseo6998@unist.ac.kr; School of Biological Sciences, Seoul National University Seoul 08826 Korea

## Abstract

We developed a proximity photo-crosslinking method (*Spotlight*) with a 4-azido-*N*-ethyl-1,8-naphthalimide (AzNP) moiety that can be converted to reactive aryl nitrene species using ambient blue light-emitting diode light. Using an AzNP-conjugated HaloTag ligand (VL1), blue light-induced photo-crosslinked products of various HaloTag-conjugated proteins of interest were detected in subcellular spaces in live cells. Chemical or heat stress-induced dynamic changes in the proteome were also detected, and photo-crosslinking in the mouse brain tissue was enabled. Using *Spotlight*, we further identified the host interactome of SARS-CoV-2 nucleocapsid (N) protein, which is essential for viral genome assembly. Mass analysis of the VL1-crosslinked product of *N*-HaloTag in HEK293T cells showed that RNA-binding proteins in stress granules were exclusively enriched in the cross-linked samples. These results tell that our method can reveal the interactome of protein of interest within a short distance in live cells.

## Abstract 2

We developed a spatiotemporal proximity photo-crosslinking by visible light activation (*Spotlight*) method to capture protein–protein interactions in live systems.

## Graphical abstract

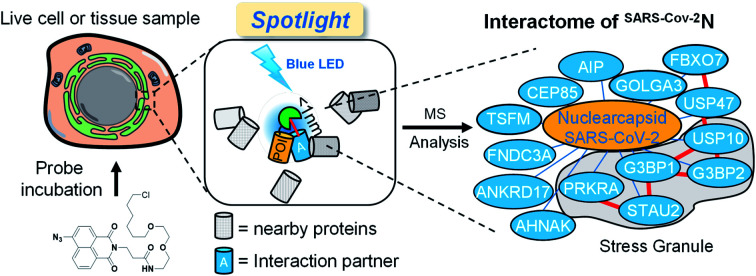

## Introduction

Most proteins physically interact with other proteins and form macromolecular complexes to perform their biological function(s) in living cells. However, only a few methods are available to reliably reveal unknown protein–protein interaction (PPI) networks in live cells. Recently, proximity labeling (PL) was developed based on *in situ*-generated reactive species using genetically encodable enzymes such as ascorbate peroxidase (APEX)^1^ or promiscuous biotin ligase (BioID^2^ or TurboID^3^). The reactive species generated by APEX or BioID/TurboID are covalently conjugated to proximal proteins near the enzymes in live cells and analyzed using mass spectrometry after cell lysis. This method has become increasingly popular in cell biology and has revealed local proteome information in diverse subcellular compartments within a live cellular context.^4^

**Fig. 1:**
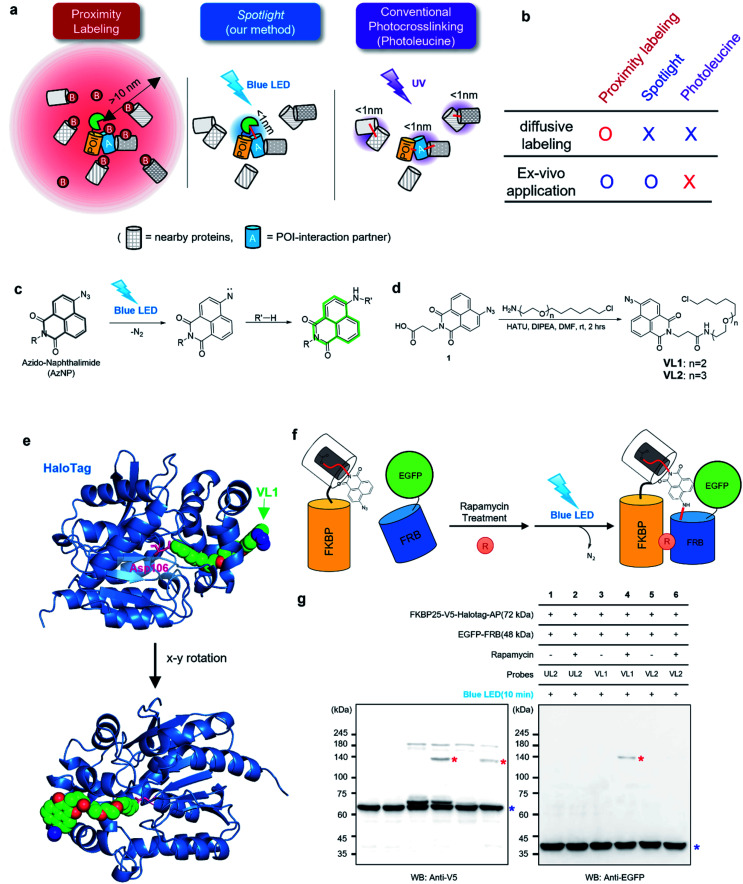
AzNP moiety-mediated proximity photo-crosslinking reaction by visible light in live cells. (a) Scheme of proximity labeling (left), spotlight (middle) and conventional photocrosslinking method (right). (b) Comparison of methods described in (a). (c) Photo-activation reaction mechanism of the AzNP moiety (above) (d) synthetic scheme of its HaloTag-conjugated version probe, VL1 (below, see ESI† for further details). (e) X-ray structure of co-crystalized HaloTag and VL1. Scheme (f) and western blot results (g) of VL1-mediated FKBP-FRB photo-crosslinking in the presence of rapamycin under blue LED light illumination. Photo-crosslinked products of FKBP25-V5-HaloTag and EGFP-FRB are marked with red asterisks. Non-cross-linked FKBP25-V5-HaloTag and EGFP-FRB are marked with blue asterisks in the anti-V5 and anti-EGFP western blot results, respectively. Ponceau S stain images of cell lysates are shown in Fig. S2b and c.†

The labeling radius of APEX, BioID/TurboID, and other PL tools (*e.g.*, T-Rex,^5^ PhotoPPI,^6^ MicroMap^7^) is estimated to range from a few nm to hundreds of nanometers, depending on the lifetime of the reactive species and local protein concentration in live cells.^4–6^ This labeling radius is suitable for sub-compartmental proteome mapping;^4^ however, this radius is rather diffusive for the identification of the physical interacting partners (*i.e.*, interactome analysis) of proteins of interest (POI) (Fig. 1a). For interactome mapping, genetically encoded chemical crosslinkers have also been used to capture PPI networks and simultaneously capture the nucleophilic residues of nearby proteins for crosslinking^8–10^ although this system requires the expression of multiple subunits for the metabolic incorporation of unnatural amino acid (UAA).

On the other hand, more controllable photo-crosslinking reactions also have been utilized to capture the physical interactome.^11^ These methods utilizes aryl azide, diazo, or diazirine moieties that can be converted to reactive nitrene or carbene species by ultraviolet (UV) light activation (Fig. 1a). Since these species usually have a short lifetime (*T*_1/2_ <2 ns)^7^ in aqueous solution, they can conduct an N–H or C–H insertion reaction with physically interacting proteins (Fig. S1b†). Photo-crosslinking methods using UAAs such as photo-methionine or photo-leucine^12^ or those containing diazirine moieties enable photo-crosslinking reaction of the proteins into which UAAs are incorporated *via* the translational machinery in live cells. However, this method faces two critical issues: (i) toxicity from proteome-wide nonspecific incorporation of UAAs (especially for the photo-leucine method^13^), and (ii) UV light irradiation that has detrimental consequences for a live system.^14^ To overcome the issues of current PL and photo-crosslinking techniques (Fig. 1b), we here introduce a new method that can perform a photo-crosslinking reaction with proximal proteins using a visible light-activable chemical ligand (*Spotlight*) in a live cell. This system can be envisioned having two parts: (i) visible light-activable photo-crosslinking moiety and (ii) selective delivery of this moiety to the POI, thus addressing toxicity from the proteome-wide incorporation of UAAs. Our literature search has revealed that most of the available visible light-activable moieties (*e.g.*, benzoyl azide,^15^ vinyl azide^16^) are unstable in a physiological system. Among the photo-crosslinkable handles, naphthyl azide-based 4-azido-*N*-ethyl-1,8-naphthalimide (AzNP) (Fig. 1c) attracted our interest for the following reasons: (1) it is highly stable in aqueous solutions, and has been widely used in hydrogen sulfide sensors;^17,18^ (2) it emits strong green fluorescence, which has been used in the fluorogenic imaging of alkyne-modified biomolecules^19^ in live mammalian cells; and (3) it shows good photo-crosslinking ability: upon UV illumination, AzNP can convert to the aryl nitrene species, which can covalently attach to proteins.^14^ However, the visible light-induced photo-crosslinking activity of AzNP in live cells has not been explored to date.^14^

We hypothesized that AzNP can be utilized for capturing proximal proteins under a photo-crosslinking reaction if it is delivered selectively to the proximal site of the POI. Currently, engineered aminoacyl tRNA synthetases^12,20,21^ and post-translationally modified enzymes (*i.e.*, LplA^11^) have been utilized to deliver a small-size UV-cross-linkable moiety (*i.e.*, benzylazide,^22,23^ diazirine^24,25^); however, it is highly unlikely that the currently engineered enzymes are able to deliver bulky AzNP due to the limited size of active sites. For this reason, we utilized the HaloTag system to efficiently deliver AzNP to the POI. HaloTag is an engineered tag protein that can covalently capture the haloalkane ligand within a few minutes in live cells with high efficiency.^26,27^ Conjugated with haloalkane or HaloTag ligand (HTL), any chemicals (*e.g.*, fluorophores,^26,28,29^ VHL binders,^30^ kinase inhibitor^31,32^) could be decorated with HaloTag with a nearly 100% conjugation yield in a covalent fashion while the haloalkane is buried in the cavity of HaloTag.^33^ Since protein-ligand binding interactions and various “in-cell chemical reactions,” including the strain-promoted click reaction^34^ and photo–activation reactions,^5,35^ have been successfully realized with HaloTag, we expected HaloTag to be a suitable platform for our proximity photo-crosslinking system using an AzNP-conjugated ligand.

## Results

### AzNP conjugated HaloTag ligand can cross-link proximal proteins in live cells under ambient blue light activation

To test this model, we synthesized AzNP-conjugated HaloTag ligands (VL1 and VL2) with two linker sizes (Fig. 1d, see ESI†). VL1 showed fairly strong absorbance in the visible light range (*λ*_max_ = 375 nm, extinction coefficient value = 2230 M^−1^ cm^−1^; Fig. S3†). For control experiments, we also prepared other photo-crosslinkable HaloTag ligands that contain short-range UV light-absorbing *para*-azidophenyl and diazirine moieties (UL1–4, Fig. S1c†).^36^ After chemical synthesis and characterization of the ligands (see ESI†), X-ray protein structure analysis of the co-crystalized holo–protein complex of HaloTag-UL2 (Fig. S1d†) and Halotag-VL1 (Fig. 1e) was respectively used to confirm that both the *para*-azidophenyl moiety of UL2 and the AzNP moiety of VL1 are well exposed at the surface of HaloTag.

We next tested the photo-crosslinking activities of the ligands using the rapamycin-inducible FKBP25-FRB protein complex^37^ by co-expression of FKBP25-HaloTag (64 kDa) and EGFP-FRB (48 kDa) in HEK293T cells (Fig. S2a†). In this model system, VL1 successfully generated the photo-crosslinked product of FKBP25-HaloTag and EGFP-FRB that appeared around 120 kDa only in the presence of rapamycin and in the blue LED light illumination condition (Fig. 1f and g). In this reaction on the FKBP25-HaloTag, VL1 containing the PEG1 linker showed stronger crosslinking efficiency with EGFP-FRB compared with that of VL2 containing the PEG2 linker (Fig. 1g). The VL1-mediated photo-crosslinked product of HA-FRB (13 kDa) and FKBP25-V5-HaloTag appeared at around 80 kDa, which is close to the expected molecular weight of the cross-linked product of HA-FRB and FKBP25-V5-HaloTag (Fig. S2d†). This experiment successfully demonstrated that VL1 selectively cross-links the interactome of a HaloTag-conjugated POI (POI-HaloTag), independent of the size of the tag protein on the interacting partner.

**Fig. 2:**
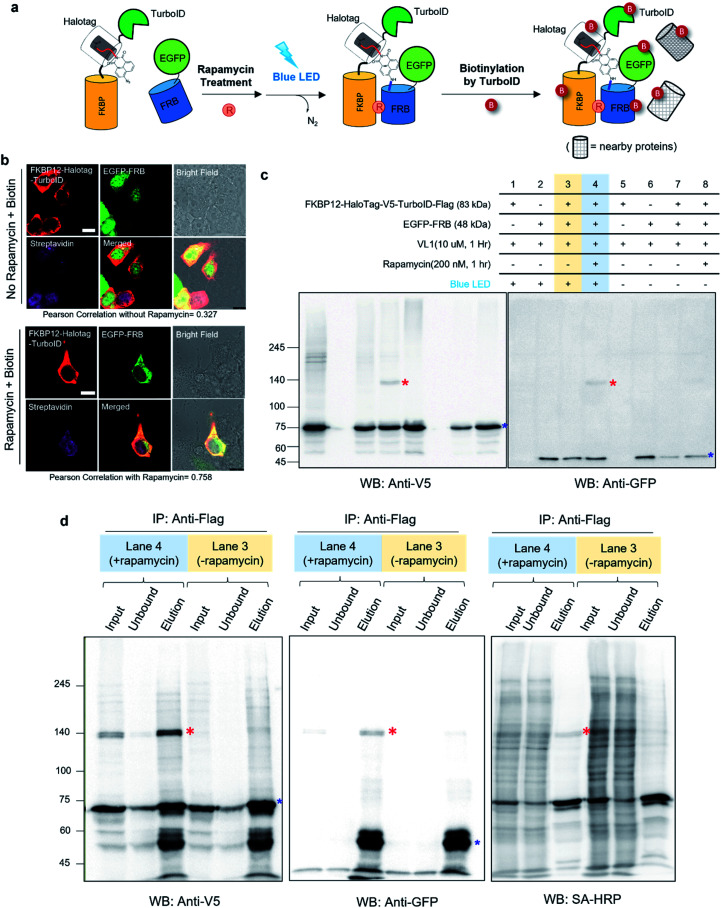
Spotlight selectivity captures interacting proteins in live cells (a) scheme of sequential reactions of VL1-mediated photo-crosslinking and biotin labeling of FKBP12-V5-HaloTag-TurboID in live cells. (b) Confocal imaging of FKBP12-V5-HaloTag-TurboID and EGFP-FRB expression under rapamycin (100 nM) or vehicle treatment for 1 h in HEK293T cells. Cells were fixed and permeabilized after biotin (50 μM) incubation for 30 min. FKBP25-V5-HaloTag-TurboID was visualized by anti-V5 antibody and anti-mouse Alexa Fluor 568; biotinylated proteins were visualized using SA-647 antibody after fixation and permeabilization. The green channel shows the EGFP signal. Scale bar = 10 μm. (c) Western blot results of VL1-mediated FKBP12-V5-HaloTag and EGFP-FRB photo-crosslinking under blue LED light. Photo-crosslinked products are marked with red asterisks and non-crosslinked monomers are marked with blue asterisks. (d) Anti-Flag enrichment results of FKBP12-HaloTag-V5-TurboID-Flag in the samples of lane 3 and lane 4 of (c). VL1-mediated crosslinked product (red asterisk) shown in the anti-V5 and anti-GFP blot is also shown as a biotinylated protein in the SA-HRP western blot. Non-crosslinked monomers are marked with blue asterisks.

Notably, none of the other conventional photo-crosslinking ligands containing phenyl azide or diazirine moieties (*i.e.*, UL1–4) could generate the photo-crosslinked product of FKBP-HaloTag and EGFP-FRB with blue LED light activation (Fig. 1g), although UL1–4 generated a photo-crosslinked product with UV light illumination, which could be purified *via* immunoprecipitation (Fig. S1e–i†). Among these UL probes, phenyl azide-based UL2 showed robust photo-crosslinking efficiency both in live cells and in a test tube reaction under UV activation (Fig. S1i†), although phot-crosslinking was not efficient under blue LED light illumination (Fig. 1g). From these results, we could confirm that AzNP has promising photo-crosslinking ability under visible light activation. Notably, we used an ambient blue LED household lamp (∼0.057 W cm^−2^) for all of our photo-crosslinking experiments in test tubes and live cells with VL1, which highlights the sensitivity of AzNP and the requirement of only low-intensity visible light illumination. We also demonstrated that the photo-crosslinked product on the HaloTag protein appears within 1 min of blue LED illumination in the live cell experiment (Fig. S10g†), which was much faster than cross-linking in the untargeted *in vitro* reaction with bovine serum albumin (Fig. S4†) due to the macromolecular crowding environment in the live cell. These result support that our method of spatiotemporal proximity photo-crosslinking by visible light activation (*Spotlight*) can capture the temporal interacting partners under physiological conditions. We further tested whether our *Spotlight* method has a reduced labeling radius compared to the TurboID method, which is currently the most popular PL method tool for interactome mapping.^3^ Toward this end, we tagged HaloTag and TurboID on the FKBP12 protein, and determined whether FRB can be photo-crosslinked and biotinylated under rapamycin treatment (Fig. 2a). In an imaging experiment, FKBP12-HaloTag-TurboID co-localized with EGFP-FRB under rapamycin treatment, which indicated that conjugated FKBP12 is functional and can be complexed with the FRB domain (Fig. 2b). Western blotting showed that the cross-linked product of EGFP-FRB (48 kDa) and FKBP12-HaloTag-V5-TurboID (84 kDa) appeared at around ∼140 kDa only in the rapamycin-treated sample (lane 4, Fig. 2c). The marked change in the crosslinking pattern in lane 4 compared with the patterns in lanes 1 and 3 reflected a notable change in the proteomic interactome of FKBP12 following rapamycin treatment. Anti-Flag immunoprecipitation showed that the FKBP12 and FRB cross-linked product was biotinylated by SA-HRP western blot (Fig. 2d). In addition to this cross-linked FKBP12 and FRB product, numerous proteins biotinylated by TurboID were detected in the flow-through fraction that were not crosslinked with VL1. This indicated that *Spotlight* is a more stringent method to identify physically interacting proteins, whereas the PL method (*i.e.*, TurboID) labels all the nearby proteins spuriously within labeling radius (Fig. 2a).

### AzNP ligands (VL1, VL2) capture local protein networks in various subcellular compartments and can differentiate the stressed proteome in live cells

**Fig. 3:**
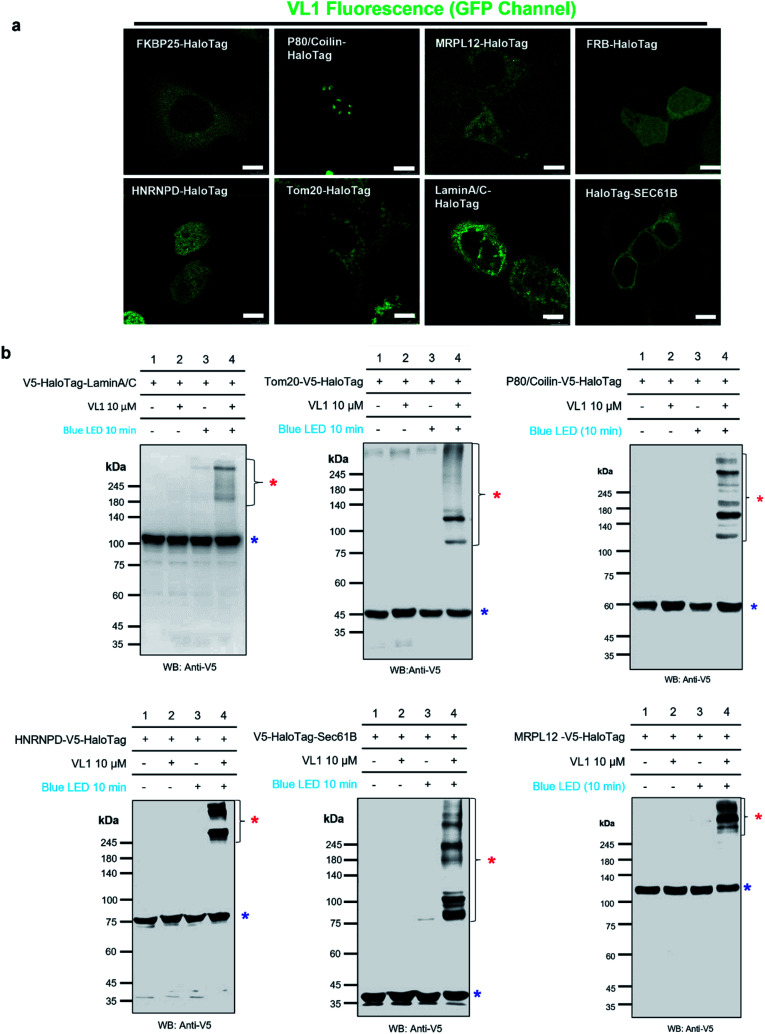
Fluorescence imaging and western blot detection of VL1-targeted HaloTag-conjugated POIs. (a) Fluorescence images of VL1 with various subcellular-localized HaloTag-conjugated POIs. Scale bar = 10 μm. VL1 fluorescence was observed in the GFP channel of confocal microscopy (excitation wavelength = 488 nm). Construct map and expected subcellular localization information are shown in Fig. S5,† and the merged immuno-fluorescence image with anti-V5 antibody stain (RFP channel) is shown in Fig. S6a.† (b) Western blot analysis of VL1-mediated photo-crosslinked products of various Halotag-V5-POIs using anti-V5 antibody. Photo-crosslinked products are marked with red asterisks and non-crosslinked POI-HaloTag proteins are marked with blue asterisks. Additional results of VL1-mediated cross-linked HaloTag-POIs under blue LED light are shown in Fig. S6b.† Reproducible results of UL2-mediated cross-linked HaloTag-POIs under UV light are shown in Fig. S6c.† For all the western blot analyses, negative control samples (no probe and/or no light illumination) were include.

We tested the specificity of AzNP-containing HaloTag ligands (VL1) to the HaloTag protein and whether it captures the local PPI network in various subcellular compartments in live cells. Since VL1 emits green fluorescence (*λ*_max_ = 540 nm, Fig. S3†) and its fluorescence is increased after the photo-crosslinking reaction (Fig. S4†), we expected that targeting of VL1 and formation of POI-HaloTag could be readily tracked in real time in live cells, whereas other UV-crosslinking ligands (*e.g.*, phenyl azide, diazirine) would not be visualized with microscope imaging due to their negligible fluorescence emission. As expected, the fluorescence imaging experiment revealed that green fluorescent VL1 was targeted to various HaloTag proteins (Fig. 3a) in various subcellular compartments, including Lamin-AC (nuclear inner membrane), p80-coilin (Cajal body), HNRNPD (SAM68 body), MRPL12 (mitochondrial matrix), Tom20 (outer mitochondrial membrane), SEC61B (endoplasmic reticulum membrane), and G3BP1 (stress granules) (Fig. 3a, S5 and S6†). Western blot analysis showed that all of the POI-HaloTag constructs in various subcellular organelles generated cross-linked products with VL1 under blue LED light illumination (Fig. 3b and S6†). Interestingly, each POI-HaloTag showed unique VL1-cross-linked patterns that might reflect their local microenvironments. This result supports that VL1 has good membrane permeability and that it can reach the inner parts of subcellular organelles such as mitochondrial matrix proteins (*i.e.*, MRPL12) to capture the interacting proteins in each compartment with blue LED light activation.

**Fig. 4:**
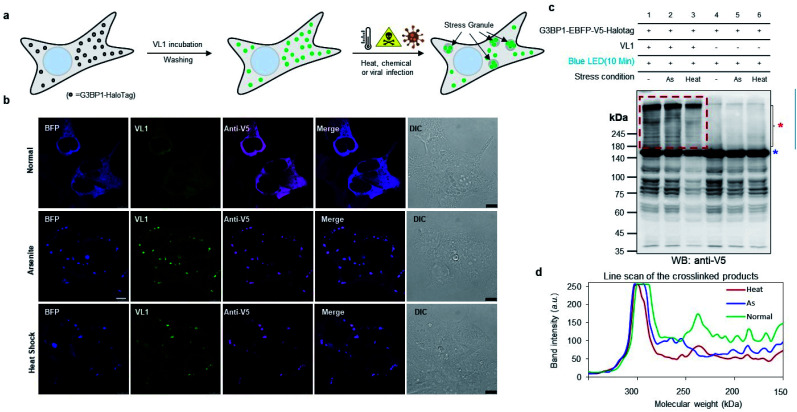
Crosslinking in stress granules and phase-separated non-membrane-bound protein complex using AzNP-conjugated HaloTag ligand (VL1) (a) scheme of monitoring stress granule formation of G3BP1-HaloTag with VL1 fluorescence under various stress conditions (*e.g.*, heat, chemical treatment, or viral infection). (b) Confocal imaging results of VL1-treated G3BP1-EBFP-V5-Halotag in the presence of 500 μM arsenite treatment or heat shock (43 °C) for 1 h. EBFP fluorescence of G3BP1-EBFP-HaloTag was observed in the BFP channel. VL1 fluorescence was observed in the GFP channel and anti-V5 immunofluorescence was visualized with a secondary antibody conjugated with Alexa Fluor 647. Scale bar = 10 μm (c) Western blot analysis of VL1-mediated cross-linked products of G3BP1-EBFP-HaloTag under the various stress conditions of (b). Samples not incubated with VL1 were used as negative controls. (d) Line scan analysis of band intensities of the cross-linked products of (c).

To check whether VL1 has stability to capture the spatiotemporal change in the interactome under stress conditions (Fig. 4a), we prepared HEK293T cells expressing HaloTag-EBFP-V5-G3BP1 (hereafter HaloTag-G3BP1). The cells were incubated with VL1 for 1 h, washed, and stimulated under oxidative stress using sodium arsenite (As_2_O_3_, 500 μM) treatment or heat stress (43 °C) for 1 h. Under these conditions, HaloTag-G3BP1 formed stress granules (*e.g.*, BFP and anti-V5 antibody), in good agreement with previous studies of G3BP1,^38^ and HaloTag-targeted VL1 fluorescence also well overlapped with BFP and anti-V5 signals (Fig. 4b). This result confirmed that VL1 can be used for real-time visualization of POIs under various stimuli. We also confirmed that the VL1-mediated photo-crosslinked products changed under the stress conditions (*e.g.*, arsenite stress and heat shock), which reflects that different protein factors accumulate at the stress granule when stimulus is varied.^39^ This result indicates that *Spotlight* can capture the change of the G3BP1 interactome (Fig. 4c and d). The change of the G3BP1 interactome was reproducibly observed in the G3BP1-GFP and GFP-binding protein (GBP)-HaloTag systems, which might be beneficial for rapid interactome mapping of the existing cloned library of the GFP-tagged POI^40^ (Fig. S7a and b†). In this system, VL1 showed more cross-linked proteins under blue LED light activation compared with those obtained using UL2 under the UV illumination, which is mainly attributed to the superior cell penetration of visible light than UV light (Fig. S7b†).

In addition, we tested whether *Spotlight* could capture the PPI of postsynaptic density protein 95 (PSD95, also known as DLG4) in an animal model. For this experiment, we prepared an adeno-associated virus that can express PSD95-HaloTag in the viral-infected region of the mouse brain. We injected this virus in the hippocampus region of the mouse brain and waited for 4 weeks for PSD95-HaloTag to be expressed in the mouse model (see ESI†). After expression of PSD95-HaloTag *via* the viral vector, VL1 (10 μM) was incubated with the sectioned slices of the mouse brain for 1 h after sacrifice, and the slices were illuminated with blue LED light for 30 min (Fig. S8a†). As shown in Fig. S8b–d,† VL1 generated photo-crosslinked products with PSD95-HaloTag in the brain tissue in a reproducible manner, whereas negative controls showed less cross-linked product. Although photo-crosslinking was not as efficient as in the cultured cells, our results demonstrated that the *Spotlight* method could be potentially applicable in animal model experiments.

### SARS-CoV-2-nucleocapsid (N) protein-HaloTag recruits RNA-binding proteins (RBPs) and viral host proteins

Motivated by the promising results in live cells, we used our *Spotlight* method to reveal the host interactome of the SARS-CoV-2 N protein.

^SARS-CoV-2^N is one of the core protein components of the viral particle, with an RNA-binding domain, which plays crucial roles in the replication and repackaging of the viral genome in host cells. Obtaining a better understanding of host proteins is essential for studying the viral replication mechanism in host cells. The host interactome of SARS-CoV-2 has been investigated to date using affinity purification mass spectrometry (AP-MS)^41^ and conventional PL methods.^42^ However, AP-MS data may reveal artificial interaction partners that can be enriched in the lysate condition, and PL may exhibit non-physiological interaction partners due to the aforementioned diffusive labeling characteristics of current PL methods. Thus, we expected that our *Spotlight* method would provide another important layer of information about the physical interactome of ^SARS-CoV-2^N.

**Fig. 5:**
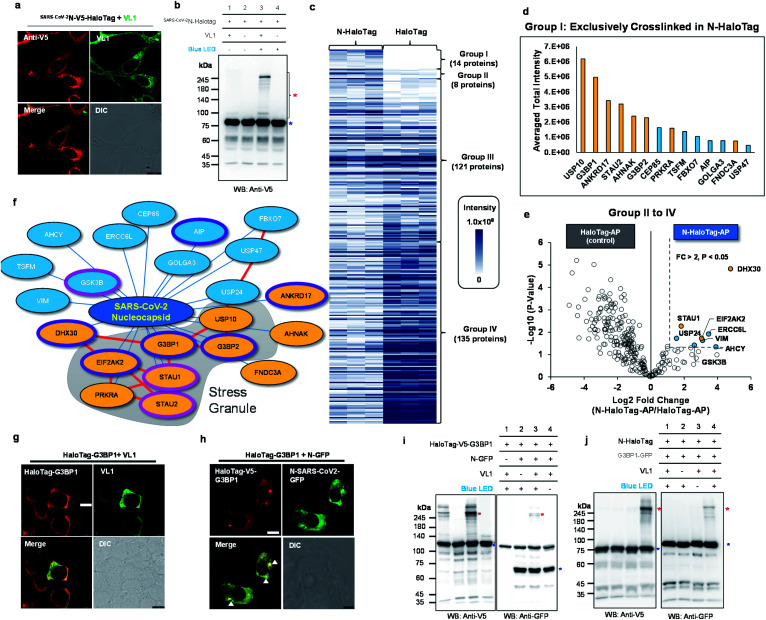
Spotlight reveals the host interactome of nucleocapsid (N) protein of SARS-CoV-2. (a) Confocal imaging results of *N*-HaloTag (^SARS-CoV-2^*N*-V5-HaloTag). (b) Western blot results of VL1-mediated cross-linked products of *N*-HaloTag (marked with a red asterisk). Non-crosslinked *N*-HaloTag is marked with a blue asterisk. (c) Overview of VL1-crosslinked proteins of *N*-HaloTag and HaloTag. The table shows the findings for mass-analyzed proteins per triplicate biological experiment. The color intensity represents the normalized mass intensity of the peptides per identified VL1-crosslinked protein with *N*-HaloTag or HaloTag. Detailed information is shown in ESI Dataset 3† (d) normalized mass intensity of 14 proteins of Group I from (c). RBPs are colored in yellow and non-RBPs are colored in blue. (e) Volcano plot of Groups II to IV proteins of (c) showing statistically significant enrichment of VL1-crosslinked proteins of *N*-HaloTag (8 proteins, Group II) over the VL1-crosslinkd proteins of the HaloTag only; see ESI Dataset 3† for details. (f) SARS-CoV-2 N primarily associates with stress granule proteins, and interactors are enriched for RNA-binding proteins (RBP). Proteins are color-coded based on the RBP (yellow) and non-RBP (blue) functions (see ESI Dataset 3†), and those involved in viral host protein and anti-viral signaling are circled in pink and purple lines, respectively. Protein network information in STRING database (https://string-db.org/) was marked with red lines (g and h) Confocal microscopy imaging of HaloTag-G3BP1 with VL1 (g) and HaloTag-G3BP1 with co-expressed *N*-GFP (h) in HEK293T cells. HaloTag-V5-G3BP1 was visualized by anti-V5 antibody (AF568-conjugated secondary antibody, red fluorescence channel) and VL1 or *N*-GFP was observed in the GFP channel. Arrows mark stress granule formation. Scale bar = 10 μm. (i) VL1-crosslinked product of HaloTag-V5-G3BP1 and *N*-GFP observed both in anti-V5 and anti-GFP western blot results. (j) VL1-crosslinked product of *N*-HaloTag and G3BP1-GFP observed both in anti-V5 and anti-GFP western blot results. Photo-crosslinked products are marked with red asterisks and non-crosslinked *N*-HaloTag proteins are marked with blue asterisks. See additional validation results in Fig. S12 and S13.†

To reveal the host interactome network of ^SARS-CoV-2^N in human cells, we cloned the ^SARS-CoV-2^*N*-V5-HaloTag-AP construct (hereafter ^SARS-CoV-2^*N*-HaloTag) and generated the stable cell line in HEK293T-rex cells. From sequential imaging and western blot experiments, VL1 was successfully targeted to the ^SARS-CoV-2^*N*-HaloTag protein and cross-linked the neighboring proteins (Fig. 5a and b). Moreover, nearly all of the cells expressing ^SARS-CoV-2^*N*-HaloTag were labeled with VL1, as post-treated biotin-HTL was negligibly conjugated with ^SARS-CoV-2^*N*-HaloTag after VL1 treatment (Fig. S9a and b†). In addition, VL1 showed robust cross-linking efficiency on ^SARS-CoV-2^*N*-HaloTag compared with other photo-crosslinking ligands (VL2 and UL probes) under blue LED light (Fig. S9c–f†), and its photo-crosslinking reaction on ^SARS-CoV-2^*N*-HaloTag was efficiently complete within 1 min of blue LED light illumination (Fig. S9g and h†).

Using this construct, we prepared triplicate biological samples for liquid chromatography-tandem mass spectrometry (LC-MS/MS) analysis with control samples (see Fig. S10†). For enrichment of the VL1-crosslinked product of ^SARS-CoV-2^*N*-HaloTag, we utilized acceptor peptide (AP, also known as Avitag), which was genetically conjugated at the C-terminus of ^SARS-CoV-2^*N*-HaloTag construct, and can be enriched on streptavidin (SA) beads after an *in vitro* biotinylation reaction using purified biotin ligase (BirA).^43^ We confirmed that biotinylation of ^SARS-CoV-2^*N*-HaloTag occurred by the addition of BirA and biotin/ATP in the mixture, and no or negligible biotinylated ^SARS-CoV-2^*N*-HaloTag remained in the flow-through fraction after SA-bead enrichment given the femtomolar binding affinity between biotin and streptavidin (Fig. S10†).^44^ This strong binding affinity enables using 10% sodium dodecyl sulfate (SDS) buffer as an incubation and washing buffer through the enrichment step, which is beneficial for efficient clearing of non-covalent binders on bait proteins. Indeed, in the no VL1 cross-linked and biotinylated ^SARS-CoV-2^*N*-HaloTag-AP sample, only four proteins (including the bait protein) were found to be significantly enriched compared with the non-biotinylated sample (Fig. S12a†). By contrast, the VL1-crosslinked and biotinylated *N*-HaloTag-AP sample (*i.e.* +VL1, +BirA) showed 161 proteins, indicating that numerous VL1-crosslinked proteins survived with *N*-HaloTag-AP protein (Fig. S12b†). Since there is the possibility that some unknown endogenous proteins may have intrinsic affinity to HaloTag and could also be cross-linked with VL1, we also prepared a free HaloTag-AP construct and confirmed its VL1 binding affinity; we also generated a VL1 cross-linked product under blue LED light illumination (Fig. S13a,† b). Following the same protocol of the mass analysis (Fig. S10†), HaloTag-AP results provided detail background protein information of *Spolight* and it enables to filter the VL1-crosslinked protein information of ^SARS-CoV-2^*N*-HaloTag-AP to obtain *N*-interactome.

By comparison of MS1 signal intensity in both samples,14 proteins (G3BP1, G3BP2, STAU2, AHNAK, ANKRD17, PRKRA, CEP85, FBXO7, FNDC3A, GOLGA3, TSFM AIP, USP10, USP47) that were exclusive to the VL1-crosslinked *N*-HaloTag sample (Group I, Fig. 5d). Additionally, among the overlapping proteins between *N*-HaloTag and HaloTag (Groups II to IV), another eight proteins (ERCC6L, USP24, GSK3B, STAU1, EIF2AK2, VIM, AHCY and DHX30) were significantly (*i.e.*, *p* < 0.05, fold change > 2) enriched in the VL1-crosslinked *N*-HaloTag sample over the VL1-crosslinked HaloTag-AP sample (Fig. 5e). From this relative quantitative analysis, we could select a total of 22 proteins as the “*N*-interactome” (Fig. 5f). Among these proteins, 11 (USP10, G3BP1, ANKRD17, STAU2, AHNAK, G3BP2, PRKRA, FNDC3A, STAU1, EIF2AK2 and DHX30) are characterized as RBPs.^45^ This ratio of RBPs in the *N*-interactome (50%, 11/22) is significantly higher than that in the human proteome (∼5%, 1072/20 380). Notably, 8 of the 22 proteins (36%) in the *N*-interactome are also found in the stress granules (*e.g.*, G3BP1, G3BP2, STAU1, STAU2,^46^ USP10, PRKRA, EIF2AK2 and CEP85)^47^ where viral RNA can reside.^48^ As shown in ESI Dataset 3,† PABPC1 and THRAP3, which are well-known stress granule resident RBPs, did not cross-link *N*-HaloTag-AP but were enriched in the cross-linked sample with the free HaloTag-AP. Our data suggest that ^SARS-CoV-2^N does not promiscuously interact with all resident stress granule proteins. Interestingly, the functions of RBPs in our *N*-interactome are mainly related to viral host proteins or anti-viral processes. For instance, STAU1 is a double-stranded RNA-binding protein that stabilizes viral genomic RNA^49^ and is found in retroviral particles.^50^ HIV-1 virus replication is facilitated bySTAU2 which interacts with viral protein Rev positively regulating the RNA export.^51^ In addition, GSK3B is known to promote the HCV virion assembly and release.^52^ In contrast, G3BP1, G3BP2, AIP, EIF2AK2, DHX30, ANKRD17,^53–56^ are involved in the anti-viral response (see ESI Dataset 3†). To validate whether ^SARS-CoV-2^N and the *N*-interactome are within the radius of VL1-mediated cross-linking, G3BP1 was selected because it is a well-known RBP and stress granule marker protein.^47,57^ We prepared a HaloTag-G3BP1 construct in which the entire HaloTag is conjugated at the N-terminus of G3BP1, which showed a cytoplasmic expression pattern in the basal state and VL1 anchoring did not affect this state (Fig. 5g). However, when ^SARS-CoV-2^*N*-GFP was co-expressed, HaloTag-G3BP1 formed granules where ^SARS-CoV-2^N was co-localized, which supports that G3BP1 and ^SARS-CoV-2^N are at a close distance (Fig. 5h). Western blot analysis showed that HaloTag-G3BP1 forms a VL1-crosslinked product with ^SARS-CoV-2^*N*-GFP under blue LED light illumination (Fig. 5i) and ^SARS-CoV-2^*N*-HaloTag also formed a VL1-crosslinked product with G3BP1-GFP (Fig. 5j), we also carried out anti-Flag IP of N protein using *N*-Halotag-TurboID-Flag with and without co-expression of G3BP1-GFP and after western blot we could observe G3BP1 in the enriched fraction whereas no GFP signal was observed in the flowthrough fraction (Fig. S14†). We further verified that this cross-linked product was specifically generated with the HaloTag-G3BP1 construct (not G3BP1-HaloTag) (Fig. S15a†). These results indicated that the G3BP1 and ^SARS-CoV-2^N interaction might occur at the N-terminal domain of G3BP1. Moreover, when using the HaloTag-2xTEV-Flag-G3BP1 construct, which has a longer linker (33 amino acids) than Halotag-Flag-G3BP1 (11 amino acids), almost identical cross-linking results were obtained, demonstrating that cross-linking with this system is independent of the linker size between HaloTag and the POI (Fig. S15b†). These results confirmed that G3BP1 and ^SARS-CoV-2^N physically interact with each other. Our results are in good agreement with recent publications showing that G3BP1 is a physical interacting partner of ^SARS-CoV-2^N based on various validation methods.^41,58^

## Discussion

In this study, we confirmed that AzNP is a promising photo-activable chemical moiety for our visible light-inducible proximity cross-linking method (*Spotlight*), and our method could directly enrich the physiological interaction partners of SARS-CoV-2 N protein. In the current system, we used HaloTag as a model system for genetic targeting of the AzNP ligand to the POIs in a live model. HaloTag has been utilized as a universal tag for many POIs in diverse types of cellular experiments. Recently, experiments with HaloTag-CRISPR knock-in cell lines and a CRISPR knock-in mouse model (*i.e.*, Titin-HaloTag^59^) showed that HaloTag itself may not disturb the physiology of the conjugated POI. Thus, we believe that VL1 can be utilized for many POIs to which HaloTag can be genetically conjugated without functional perturbation. Furthermore, our method is not limited to the use of HaloTag, and it can be adapted to other chemical ligand-protein tagging systems using markedly smaller tag proteins (*e.g.*, SNAP-tag and CLIP-tag) in the future, which might be helpful to cover more specific interacting partners of POIs.

The Photo-methionine or Photo-leucine method^24^ that can incorporate UV-activable photo-crosslinking amino acids in newly synthesized proteins *via* natural aminoacyl tRNA synthetases at the proteome-wide level is currently one of the popular methods for the identification of the interactome using a photo-crosslinking reaction. However, this method has not been utilized in a mouse model to date, because animal-level incorporation of UAA is required for the experiment, which can cause toxicity and physiological damage to the model. Compared with this method, we specifically targeted cross-linkable ligands to only the HaloTag-POI, which is cost-effective and also free from toxicity issues. We also showed that *Spotlight* enabled target protein cross-linking in the mouse brain using PSD95-HaloTag.

TurboID has been utilized for interactome mapping in diverse experiments; however, owing to its rather diffusive labeling characteristics of biotin-AMP, the current PL methods (*i.e.*, BioID, TurboID, and APEX) require a “filtration approach” by comparison of the data with biotinylated proteins of cytosolic TurboID (*e.g.*, TurboID-NES).^60^ However, these filtered data still cannot reflect the direct binding partners selectively but rather only reflect the unclear “contour” of proximal proteins compared to the cytosolic background, and these data cannot decipher whether the selected proteins are true interacting partners of a POI. Compared with these PL method, our approach can selectively identify the direct interacting partners of target proteins, which is beneficial for direct identification of the molecular network of a POI in live cells. Furthermore, we expect that our *Spotlight* method can be utilized with TurboID for the same target POI because the photo-crosslinking reaction of *Spotlight* can orthogonally occur with the amide coupling-based biotinylation reaction of TurboID.

We performed mass analysis of eight samples of ^SARS-CoV-2^*N*-HaloTag-AP and HaloTag-AP with four control conditions (±VL1, ±BirA) to confirm whether each step of our experiments (*e.g.*, photo-crosslinking and streptavidin enrichment) were working as expected (Fig. S10 and S12†). For general use of our method, we consider that it might not be necessary to include all of these control samples, and it might be sufficient to compare the streptavidin-enriched sample of VL1-crosslinked POI-HaloTag (POI-HaloTag-AP/+VL1/+BirA) *versus* VL1-crosslinked free HaloTag (HaloTag-AP/+VL1/+BirA) to obtain the POI interactome proteins, as shown in Fig. 5. Our analysis result of free HaloTag cross-linked proteins (ESI Dataset 2†) might provide the background protein information for other experiments using our *Spotlight* method.

We also tested whether the efficiency of generation of cross-linked product was affected by linkers in the HaloTag-G3BP1 construct and photo-crosslinking probes at the HaloTag surface (Fig. S15a and b†). This result implies that the HaloTag position at the N-terminus of G3BP1 can have some level of flexibility of approach to the surface of ^SARS-CoV-2^N protein, which seems to be practically unaffected by the peptide linker length between HaloTag and G3BP1, whereas the PEG linker length of the HaloTag ligand can determine the orientation of active species at the HaloTag surface^61^ that may affect the cross-linking efficiency to the specific interaction partner. These features of positioning-dependent cross-linking efficiency due to the intrinsically short labeling radius of our method might be a limiting factor to cover the complete physical interactome of a POI. However, this might also be considered a strength of the current method because any cross-linked proteins in our system should be interacting proteins of POIs.

In this study, we performed a proof of concept ex vivo experiment of *Spotlight* using mouse brain samples, and it showed lower crosslinking efficiency than that in the cultured cells possibly owing to the low penetration of blue light. We envision that our *Spotlight* method can be further improved by using other probes that can be activated by infrared light, such as diazocoumarin,^62^ although these dyes need to be tested whether their targeting and the lifetime of carbene on the target protein surface can be sufficient to effectively react with the residues on the proximal proteins in the future.

## Conclusions

In summary, we have developed a new blue light inducible proximity photocrosslinking method “*Spotlight*” and demonstrated its the ability to identify protein–protein interaction in cell as well as crosslinking in the tissue samples. We successfully demonstrated our probe's ability to capture the interacting partner selectively in model system and we successfully identify improved interactome of ^SARS-CoV-2^N protein in live cells. From these results, we believe that our new method can be utilized to identify specific interactome of various POIs in live system.

## Data availability

Our raw mass analysis files were deposited to the ProteomeXchange (http://proteomecentral.proteomexchange.org) *via* the PRIDE partner repository with identifier PXD027149. The atomic coordinates and structure factors of the crystal structures have been deposited in the Protein Data Bank (PDB) under accession codes 7WAM (HaloTag-VL1, https://www.rcsb.org/structure/7WAM) and 7WAN (HaloTag-UL2, https://www.rcsb.org/structure/7WAN).

## Author contributions

P. K. M. synthesized the chemical probes, did cloning for HaloTag constructs, prepared western blot for crosslinking/crosslinking-biotinylation, confocal and IP; M. G. K. performed cloning, confocal imaging, western blotting, and mass sample preparation, H. L. did the co-crystallization of UL2 and VL1 with HaloTag, N. S. did confocal imaging, all authors contributed to this work; PKM, MGK and RHW wrote the manuscript from inputs of all the authors.

## Conflicts of interest

The authors declare no competing interests.

## Supplementary Material

SC-013-D1SC04871F-s001

SC-013-D1SC04871F-s002

SC-013-D1SC04871F-s003

SC-013-D1SC04871F-s004

## References

[cit1] Rhee H. W., Zou P., Udeshi N. D., Martell J. D., Mootha V. K., Carr S. A., Ting A. Y. (2013). Science.

[cit2] Roux K. J., Kim D. I., Raida M., Burke B. (2012). J. Cell Biol..

[cit3] Branon T. C., Bosch J. A., Sanchez A. D., Udeshi N. D., Svinkina T., Carr S. A., Feldman J. L., Perrimon N., Ting A. Y. (2018). Nat. Biotechnol..

[cit4] Zhou Y., Zou P. (2020). Curr. Opin. Chem. Biol..

[cit5] Parvez S., Fu Y., Li J., Long M. J. C., Lin H.-Y., Lee D. K., Hu G. S., Aye Y. (2015). J. Am. Chem. Soc..

[cit6] McCutcheon D. C., Lee G., Carlos A., Montgomery J. E., Moellering R. E. (2020). J. Am. Chem. Soc..

[cit7] Geri J. B., Oakley J. V., Reyes-Robles T., Wang T., McCarver S. J., White C. H., Rodriguez-Rivera F. P., Parker Jr D. L., Hett E. C., Fadeyi O. O., Oslund R. C., MacMillan D. W. C. (2020). Science.

[cit8] Yang B., Tang S., Ma C., Li S.-T., Shao G.-C., Dang B., DeGrado W. F., Dong M.-Q., Wang P. G., Ding S., Wang L. (2017). Nat. Commun..

[cit9] Liu C., Wu T., Shu X., Li S.-T., Wang D. R., Wang N., Zhou R., Yang H., Jiang H., Hendriks I. A., Gong P., Zhang L., Nielsen M. L., Li K., Wang L., Yang B. (2021). Adv. Biol..

[cit10] Liu J., Cao L., Klauser P. C., Cheng R., Berdan V. Y., Sun W., Wang N., Ghelichkhani F., Yu B., Rozovsky S., Wang L. (2021). J. Am. Chem. Soc..

[cit11] Mishra P. K., Yoo C. M., Hong E., Rhee H. W. (2020). Chembiochem.

[cit12] Suchanek M., Radzikowska A., Thiele C. (2005). Nat. Methods.

[cit13] Kohl B., Brüderlin M., Ritz D., Schmidt A., Hiller S. (2020). J. Proteome Res..

[cit14] Singha M., Roy S., Pandey S. D., Bag S. S., Bhattacharya P., Das M., Ghosh A. S., Ray D., Basak A. (2017). Chem. Commun..

[cit15] Brachet E., Ghosh T., Ghosh I., König B. (2015). Chem. Sci..

[cit16] Borra S., Borkotoky L., Newar U. D., Kalwar A., Das B., Maurya R. A. (2019). Org. Biomol. Chem..

[cit17] Montoya L. A., Pluth M. D. (2012). Chem. Commun..

[cit18] Guo Y., Zeng T., Shi G., Cai Y., Xie R. (2014). RSC Adv..

[cit19] Sawa M., Hsu T.-L., Itoh T., Sugiyama M., Hanson S. R., Vogt P. K., Wong C.-H. (2006). Proc. Natl. Acad. Sci. U.S.A..

[cit20] Ryu Y., Schultz P. G. (2006). Nat. Methods.

[cit21] Yang Y., Song H., He D., Zhang S., Dai S., Lin S., Meng R., Wang C., Chen P. R. (2016). Nat. Commun..

[cit22] Baruah H., Puthenveetil S., Choi Y.-A., Shah S., Ting A. Y. (2008). Angew. Chem., Int. Ed. Engl..

[cit23] Pinney K. G., Katzenellenbogen J. A. (1991). J. Org. Chem..

[cit24] Suchanek M., Radzikowska A., Thiele C. (2005). Nat. Methods.

[cit25] Mackinnon A. L., Taunton J. (2009). Curr. Protoc. Chem. Biol..

[cit26] Ohana R. F., Encell L. P., Zhao K., Simpson D., Slater M. R., Urh M., Wood K. V. (2009). Protein Expression Purif..

[cit27] Wilhelm J., Kuhn S., Tarnawski M., Gotthard G., Tunnermann J., Tanzer T., Karpenko J., Mertes N., Xue L., Uhrig U., Reinstein J., Hiblot J., Johnsson K. (2021). Biochemistry.

[cit28] Los G. V., Encell L. P., McDougall M. G., Hartzell D. D., Karassina N., Zimprich C., Wood M. G., Learish R., Ohana R. F., Urh M., Simpson D., Mendez J., Zimmerman K., Otto P., Vidugiris G., Zhu J., Darzins A., Klaubert D. H., Bulleit R. F., Wood K. V. (2008). ACS Chem. Biol..

[cit29] Frei M. S., Hoess P., Lampe M., Nijmeijer B., Kueblbeck M., Ellenberg J., Wadepohl H., Ries J., Pitsch S., Reymond L., Johnsson K. (2019). Nat. Commun..

[cit30] Buckley D. L., Raina K., Darricarrere N., Hines J., Gustafson J. L., Smith I. E., Miah A. H., Harling J. D., Crews C. M. (2015). ACS Chem. Biol..

[cit31] Friedman Ohana R., Levin S., Wood M. G., Zimmerman K., Dart M. L., Schwinn M. K., Kirkland T. A., Hurst R., Uyeda H. T., Encell L. P., Wood K. V. (2016). ACS Chem. Biol..

[cit32] Krishnamurty R., Brigham J. L., Leonard S. E., Ranjitkar P., Larson E. T., Dale E. J., Merritt E. A., Maly D. J. (2013). Nat. Chem. Biol..

[cit33] Kang M.-G., Lee H., Kim B. H., Dunbayev Y., Seo J. K., Lee C., Rhee H.-W. (2017). Chem. Commun..

[cit34] Murrey H. E., Judkins J. C., am Ende C. W., Ballard T. E., Fang Y., Riccardi K., Di L., Guilmette E. R., Schwartz J. W., Fox J. M., Johnson D. S. (2015). J. Am. Chem. Soc..

[cit35] Lee J., Yu P., Xiao X., Kodadek T. (2008). Mol. BioSyst..

[cit36] Ai H.-w., Shen W., Sagi A., Chen P. R., Schultz P. G. (2011). ChemBioChem.

[cit37] Lee S.-Y., Lee H., Lee H.-K., Lee S.-W., Ha S. C., Kwon T., Seo J. K., Lee C., Rhee H.-W. (2016). ACS Cent. Sci..

[cit38] Markmiller S., Soltanieh S., Server K. L., Mak R., Jin W. H., Fang M. Y., Luo E. C., Krach F., Yang D. J., Sen A., Fulzele A., Wozniak J. M., Gonzalez D. J., Kankel M. W., Gao F. B., Bennett E. J., Lecuyer E., Yeo G. W. (2018). Cell.

[cit39] Yang P., Mathieu C., Kolaitis R.-M., Zhang P., Messing J., Yurtsever U., Yang Z., Wu J., Li Y., Pan Q., Yu J., Martin E. W., Mittag T., Kim H. J., Taylor J. P. (2020). Cell.

[cit40] Ariotti N., Hall T. E., Rae J., Ferguson C., McMahon K. A., Martel N., Webb R. E., Webb R. I., Teasdale R. D., Parton R. G. (2015). Dev. Cell.

[cit41] Gordon D. E., Jang G. M., Bouhaddou M., Xu J., Obernier K., White K. M., O'Meara M. J., Rezelj V. V., Guo J. Z., Swaney D. L., Tummino T. A., Hüttenhain R., Kaake R. M., Richards A. L., Tutuncuoglu B., Foussard H., Batra J., Haas K., Modak M., Kim M., Haas P., Polacco B. J., Braberg H., Fabius J. M., Eckhardt M., Soucheray M., Bennett M. J., Cakir M., McGregor M. J., Li Q., Meyer B., Roesch F., Vallet T., Mac Kain A., Miorin L., Moreno E., Naing Z. Z. C., Zhou Y., Peng S., Shi Y., Zhang Z., Shen W., Kirby I. T., Melnyk J. E., Chorba J. S., Lou K., Dai S. A., Barrio-Hernandez I., Memon D., Hernandez-Armenta C., Lyu J., Mathy C. J. P., Perica T., Pilla K. B., Ganesan S. J., Saltzberg D. J., Rakesh R., Liu X., Rosenthal S. B., Calviello L., Venkataramanan S., Liboy-Lugo J., Lin Y., Huang X.-P., Liu Y., Wankowicz S. A., Bohn M., Safari M., Ugur F. S., Koh C., Savar N. S., Tran Q. D., Shengjuler D., Fletcher S. J., O'Neal M. C., Cai Y., Chang J. C. J., Broadhurst D. J., Klippsten S., Sharp P. P., Wenzell N. A., Kuzuoglu-Ozturk D., Wang H.-Y., Trenker R., Young J. M., Cavero D. A., Hiatt J., Roth T. L., Rathore U., Subramanian A., Noack J., Hubert M., Stroud R. M., Frankel A. D., Rosenberg O. S., Verba K. A., Agard D. A., Ott M., Emerman M., Jura N., von Zastrow M., Verdin E., Ashworth A., Schwartz O., d'Enfert C., Mukherjee S., Jacobson M., Malik H. S., Fujimori D. G., Ideker T., Craik C. S., Floor S. N., Fraser J. S., Gross J. D., Sali A., Roth B. L., Ruggero D., Taunton J., Kortemme T., Beltrao P., Vignuzzi M., García-Sastre A., Shokat K. M., Shoichet B. K., Krogan N. J. (2020). Nature.

[cit42] Samavarchi-Tehrani P., Abdouni H., Knight J. D. R., Astori A., Samson R., Lin Z.-Y., Kim D.-K., Knapp J. J., St-Germain J., Go C. D., Larsen B., Wong C. J., Cassonnet P., Demeret C., Jacob Y., Roth F. P., Raught B., Gingras A.-C. (2020). bioRxiv.

[cit43] Fairhead M., Howarth M. (2015). Methods Mol. Biol..

[cit44] Hirsch J. D., Eslamizar L., Filanoski B. J., Malekzadeh N., Haugland R. P., Beechem J. M., Haugland R. P. (2002). Anal. Biochem..

[cit45] Sundararaman B., Zhan L., Blue S. M., Stanton R., Elkins K., Olson S., Wei X., Van Nostrand E. L., Pratt G. A., Huelga S. C., Smalec B. M., Wang X., Hong E. L., Davidson J. M., Lécuyer E., Graveley B. R., Yeo G. W. (2016). Mol. Cell.

[cit46] Thomas M. G., Martinez Tosar L. J., Desbats M. A., Leishman C. C., Boccaccio G. L. (2009). J. Cell Sci..

[cit47] Youn J. Y., Dyakov B. J. A., Zhang J., Knight J. D. R., Vernon R. M., Forman-Kay J. D., Gingras A. C. (2019). Mol. Cell..

[cit48] Protter D. S. W., Parker R. (2016). Trends Cell Biol..

[cit49] Chen Y. M., Ou B. T., Chen C. Y., Chan H. H., Chen C. J., Wang R. Y. (2019). Viruses.

[cit50] Mouland A. J., Mercier J., Luo M., Bernier L., DesGroseillers L., Cohen E. A. (2000). J. Virol..

[cit51] Banerjee A., Benjamin R., Balakrishnan K., Ghosh P., Banerjee S. (2014). Retrovirology.

[cit52] Kehn-Hall K., Guendel I., Carpio L., Skaltsounis L., Meijer L., Al-Harthi L., Steiner J. P., Nath A., Kutsch O., Kashanchi F. (2011). Virology.

[cit53] Yang W., Ru Y., Ren J., Bai J., Wei J., Fu S., Liu X., Li D., Zheng H. (2019). Cell Death Dis..

[cit54] Kula A., Guerra J., Knezevich A., Kleva D., Myers M. P., Marcello A. (2011). Retrovirology.

[cit55] Lahaye X., Gentili M., Silvin A., Conrad C., Picard L., Jouve M., Zueva E., Maurin M., Nadalin F., Knott G. J., Zhao B., Du F., Rio M., Amiel J., Fox A. H., Li P., Etienne L., Bond C. S., Colleaux L., Manel N. (2018). Cell.

[cit56] Garcia-Moreno M., Järvelin A. I., Castello A. (2018). Wiley Interdiscip. Rev.: RNA.

[cit57] Markmiller S., Soltanieh S., Server K. L., Mak R., Jin W., Fang M. Y., Luo E.-C., Krach F., Yang D., Sen A., Fulzele A., Wozniak J. M., Gonzalez D. J., Kankel M. W., Gao F.-B., Bennett E. J., Lécuyer E., Yeo G. W. (2018). Cell.

[cit58] Nabeel-Shah S., Lee H., Ahmed N., Marcon E., Farhangmehr S., Pu S., Burke G. L., Ashraf K., Wei H., Zhong G., Tang H., Yang J., Blencowe B. J., Zhang Z., Greenblatt J. F. (2020). bioRxiv.

[cit59] Rivas-Pardo J. A., Li Y., Mártonfalvi Z., Tapia-Rojo R., Unger A., Fernández-Trasancos Á., Herrero-Galán E., Velázquez-Carreras D., Fernández J. M., Linke W. A., Alegre-Cebollada J. (2020). Nat. Commun..

[cit60] Cho K. F., Branon T. C., Rajeev S., Svinkina T., Udeshi N. D., Thoudam T., Kwak C., Rhee H.-W., Lee I.-K., Carr S. A., Ting A. Y. (2020). Proc. Natl. Acad. Sci. U. S. A..

[cit61] Kang M. G., Lee H., Kim B. H., Dunbayev Y., Seo J. K., Lee C., Rhee H. W. (2017). Chem. Commun..

[cit62] Dai S. Y., Yang D. (2020). J. Am. Chem. Soc..

