# Supplementary material for: A chemical tool for blue light-inducible proximity photo-crosslinking in live cells
Source: Chem Sci. 2021 Dec 10;13(4):955–66. doi: 10.1039/d1sc04871f (PMC8790779; doi:10.1039/d1sc04871f)
Supplement: SC-013-D1SC04871F-s001 [file SC-013-D1SC04871F-s001.pdf]

Electronic Supplementary Material (ESI) for Chemical Science.
This journal is © The Royal Society of Chemistry 2021

Supplementary Information

A Chemical Tool for Blue-Light Inducible Proximity Photocrosslinking in Live Cells

Pratyush Kumar Mishra[1,2,†], Myeong-Gyun Kang[1,†], Hakbong Lee[3], Seungjoon Kim[4], Subin Choi[1], Nirmali Sharma[1,2], Cheol-Min Park[2], Jaewon Ko[4], Changwook Lee[3]*, Jeong Kon Seo[5,*], and Hyun-Woo Rhee[1,6,*].

[1]Department of Chemistry, Seoul National University, Seoul, 08826, Korea.

[2]Department of Chemistry, Ulsan National Institute of Science and Technology (UNIST), Ulsan ,44191, Korea

[3]Department of Biological Sciences, Ulsan National Institute of Science and Technology (UNIST), Ulsan, 44919, Korea.

[4]Department of Brain and Cognitive Sciences, Daegu Gyeongbuk Institute of Science and Technology (DGIST), Daegu, 42988, Korea.

[5]UNIST Central Research Facilities (UCRF), Ulsan National Institute of Science and Technology (UNIST), Ulsan, 44919, Korea.,

[6]School of Biological Sciences, Seoul National University, Seoul, 08826, Korea

[†] These authors equally contributed to this work.

Table of contents:

Plasmid Information……………………………..…………………………………………...……..S3

Data collection and refinement statistics…………………..…………………………..…..………S5

Figure S1………………………………………………………………………………………………S6

Figure S2………………………………………………………………………………………………S8

Figure S3……………………………………………………………………………………………S10

Figure S4……………………………………………………………………………………………S11

Figure S5……………………………………………………………………………………………S12

Figure S6……………………………………………………………………………………………S13

Figure S7……………………………………………………………………………………………S15

Figure S8……………………………………………………………………………………………S16

Figure S9……………………………………………………………………………………………S17

Figure S10…………………………………………………………………………………………..S19

Figure S11…………………………………………………………………………………………S21

Figure S12…………………………………………………………………………………………..S22

Figure S13…………………………………………………………………………………………..S24

Figure S14………………………………………………………………………..……….………...S25

Figure S15…………………………………………………………………………………………..S26

Materials and Methods……………………………………..…………………………..……..…S28

PSD95-Halotag expression in mouse brain and proximity crosslinking with VL1…………………….…S30

Photocrosslinking of N-Halotag expressing cells/Mass sample Preparation/LC-MS/MS……………………………..……………………………………………………………..S31

Probe Synthesis…………………………………………………………..……..……………………S32

Table S1. Plasmids Information

Name	Features	Promotor/ Vector	Details
Nucleocapsid (N)-HaloTag	*KpnI*-$^{SARS\text{-}CoV\text{-}2}$N-TEV-*NheI*-V5-Halotag-TEV-AviTag-Stop-*NotI*	CMV/pCDNA5	AviTag: GLNDIFEAQKIEWHE<br>V5: GKPIPNPLLGLDST<br>TEV: ENLYFQSENLYFQS
N-HaloTag-TurboID	*KpnI*-Nucleocapsid-TEV-*NheI*-HaloTag-*BamHI*-V5-TurboID-Flag-*NotI*	CMV/pCDNA5	Flag: DYKDDDDK
HaloTag-(33aa)-G3BP1	*KpnI*-AviTag-*NheI*-V5-HaloTag-*BamHI*-TEV-Flag-G3BP1-Stop-*NotI*	CMV/ pCDNA5	AviTag: GLNDIFEAQKIEWHE<br>V5: GKPIPNPLLGLDST<br>TEV: ENLYFQSENLYFQS<br>HA: YPYDVPDYA
HaloTag-(11aa)-G3BP1	*HindIII-V5*-HaloTag-*Flag-ClaI*-G3BP1-*GSG-His6-Stop*	CMV/pCDNA5	Flag: DYKDDDDK<br>V5: GKPIPNPLLGLDST
G3BP1-HaloTag	*KpnI*-G3BP1-*BamHI*-V5-Halotag-TEV-GSG-His6-Stop	CMV/pCDNA5	V5: GKPIPNPLLGLDST<br>TEV: ENLYFQSENLYFQS
G3BP1-BFP-HaloTag	*KpnI*-G3BP1-*BamHI*-Linker-EBFP-*AgeI*-V5-Halotag-TEV-His6-Stop-*XhoI*	CMV/pCDNA5	Linker: GAPGSAGSAAGSG<br>V5: GKPIPNPLLGLDST<br>TEV: ENLYFQSENLYFQS
FKBP25-V5-HaloTag-AviTag	*KpnI*-FKBP25-BamHI-*NheI*-V5-HaloTag-Avitag-Stop-*NotI*	CMV/ pCDNA5	V5: GKPIPNPLLGLDST<br>AviTag: GLNDIFEAQKIEWHE
FKBP12-HaloTag-TurboID-Flag	*SpnI-FKBP12-NheI-HaloTag-BamHI-V5-TurboID-Flag-NotI*	CMV/ pCDNA5	V5: GKPIPNPLLGLDST<br>Flag:DYKDDDDKDYKDDDDK
EGFP-FRB	*AgeI-EGFP-HindIII-EcoRI*-FRB-*Stop-BamHI*	CMV/pEGFP	EGFP: Enhanced green fluorescent protein<br>FRB: FK506 rapamycin binding domain of mTOR
V5-LaminA/C-HaloTag	*KpnI-AviTag-NheI-V5*-Halotag--*EcoRI-LaminA/C-Stop-NotI*	CMV/pCDNA5	AviTag: GLNDIFEAQKIEWHE<br>V5: GKPIPNPLLGLDST
Tom20-V5-HaloTag	*KpnI*-Tom20-Linker-*NheI*-V5-Halotag-TEV-AviTag-Stop-*NotI*	CMV/pCDNA5	Linker: GGSGDPPVAT<br>AviTag: GLNDIFEAQKIEWHE<br>V5: GKPIPNPLLGLDST
P80Coilin-V5-HaloTag	*KpnI*-p80Coilin-*BamHI*-V5-Halotag-	CMV/pCDNA5	AviTag: GLNDIFEAQKIEWHE

	TEV-AviTag-Stop-*NotI*		V5: GKPIPNPLLGLDST
MRPL12-V5-HaloTag	*KpnI*-MRPL12-*NheI*-V5-Halotag-Strep2-STOP-*NotI*	CMV/pCDNA5	V5: GKPIPNPLLGLDST<br>Strep2: WSHPQFEK
HNRNPD-V5-HaloTag	*KpnI*-HRNPD-NheI-Linker-TEV-V5-AViTag-Halotag-His6-Stop-*NotI*	CMV/pCDNA5	Linker: GGSG
V5-HaloTag-Sec61b	*AflII*-V5-Halotag-TEV-AviTag-*NheI*-Sec61Beta-Stop-*XhoI*	CMV/pCDNA5	AviTag: GLNDIFEAQKIEWHE<br>V5: GKPIPNPLLGLDST
FKBP12-EGFP	*NdeI*-FKBP12-*HindIII*-*NotI*-EGFP-*XhoI*-His6-*Stop*	T7/pET21a	His6: Six histidine for Ni-NTA affinity purification<br>EGFP: Enhanced green fluorescent protein
FRB-V5-HaloTag	*NotI*-His6-FRB-V5-SacII-HaloTag-Stop-NotI	T7/pH6HTN	His6: Six histidine for Ni-NTA affinity purification<br>FRB: FK506 rapamycin binding domain of mTOR<br>V5: GKPIPNPLLGLDST
BirA-His6	*XbaI-BirA-HindIII-NotI-His6-Stop*	T7/pET28a	His6: Six histidine for Ni-NTA affinity purification
Nucleocapsid-GFP	*KpnI*-Nucleocapsid-*BsiWI*-Linker-*NheI*-EGFP -*Stop-NotI*	CMV/pCDNA5	EGFP: Enhanced green fluorescent protein<br>Linker: GAPGSAGSAAGSG
EGFP	*KpnI*-EGFP-*Stop-NotI*	CMV/pCDNA5	EGFP: Enhanced green fluorescent protein
HA-FRB	*HindIII-HA-MluI*-FRB-*Stop*	CMV/PRK5	FRB: FK506 rapamycin binding domain of mTOR<br>HA: YPYDVPDYA

Table S2. Data Collection and Refinement Statistics

	HaloTag-UL2	HaloTag-VL1
X-ray source	Beamline 7A, PAL	Beamline 7A, PAL
Temperature (K)	100	100
Space group:	$P2_1$	$P4_32_12$
Cell parameters		
a, b, c (Å)	44.906, 72.764, 44.432	63.078, 63.078, 164.112
α, β, γ (°)	90.000, 109.300, 90.000	90.000, 90.000, 90.000
**Data processing**		
Wavelength (Å)	0.97933	0.97960
Resolution (Å)	50.00 - 2.28	50.00 - 1.49
R_{merge} (%)	12.4 (28.9)	7.0 (79.9)
I/σ	12.8 (4.0)	29.1 (2.4)
Completeness (%)	97.9 (89.2)	98.7 (97.8)
Redundancy	3.0 (2.3)	4.7 (4.8)
Measured reflections	34999	257488
Unique reflections	11723	54493
**Refinement statistics**		
Resolution (Å)	32.28 – 2.28	27.80 – 1.49
Reflections	11713	54433
Number of atoms		
Protein	2326	2353
Water	96	355
Ligand/ion	28	36
R-factor (%)	17.78	14.86
R_{free} (%)	22.83	18.83
RMSD		
Bond lengths (Å)	0.003	0.013
Bond angles (°)	0.661	1.493
B-factors ($Å^2$)		
Protein	21.37	19.34
Waters	23.42	33.77
Ligand/ion	23.44	49.12
Ramachandran plot,		
Favored regions (%)	95.14	96.92
Allowed regions (%)	4.86	3.08
Disallowed regions (%)	0	0

*Highest resolution shell is shown in parenthesis.

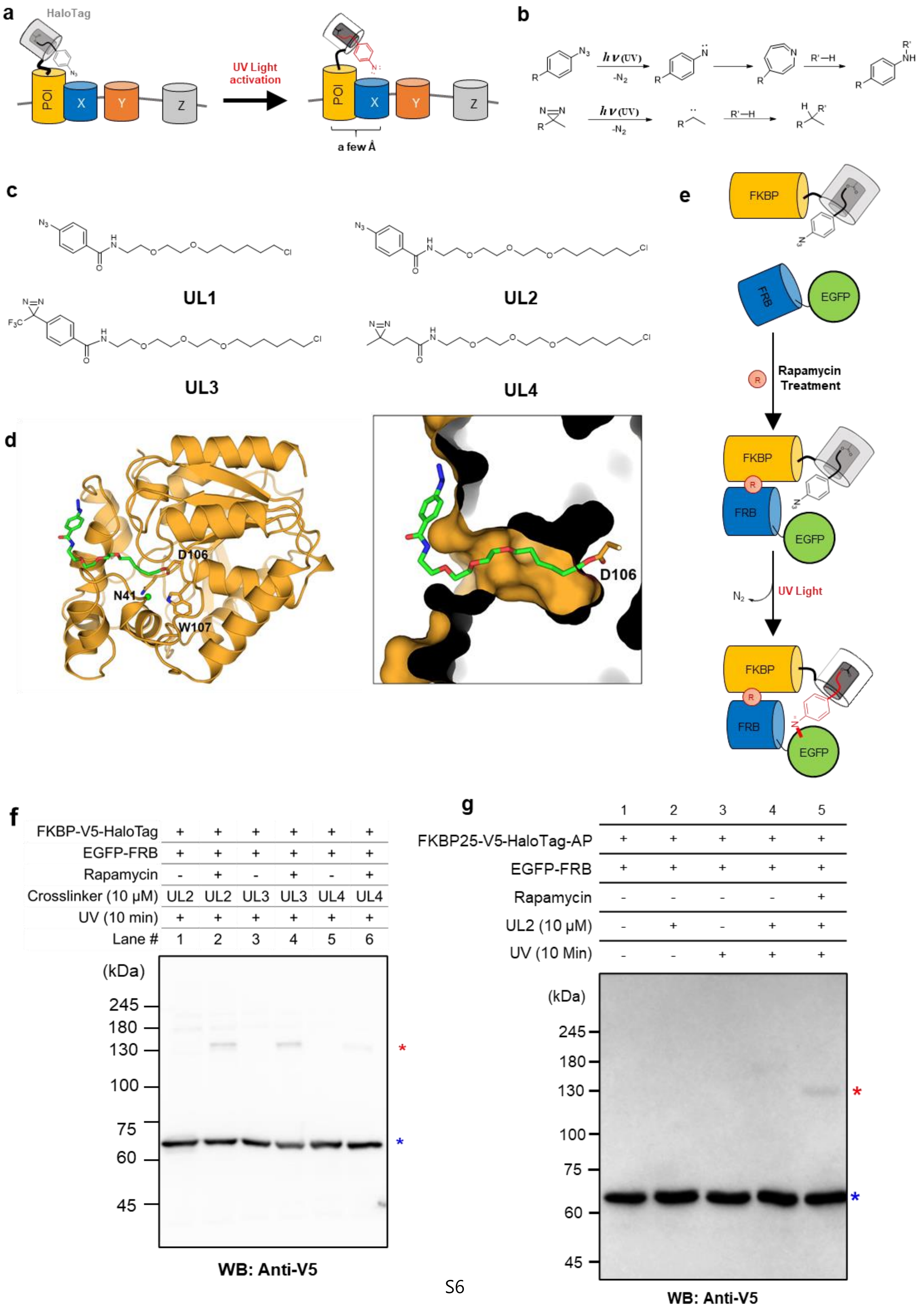

a
HaloTag
POI
X
Y
Z
UV Light activation
a few Å
b
$h\nu$(UV)
-N2
R'—H
c
UL1
UL2
UL3
UL4
d
D106
N41
W107
e
FKBP
FRB
EGFP
Rapamycin Treatment
UV Light
f
FKBP-V5-HaloTag + + + + + +
EGFP-FRB + + + + + +
Rapamycin - + - + - +
Crosslinker (10 μM) UL2 UL2 UL3 UL3 UL4 UL4
UV (10 min) + + + + + +
Lane # 1 2 3 4 5 6
(kDa)
245
180
130
100
75
60
45
WB: Anti-V5
g
1 2 3 4 5
FKBP25-V5-HaloTag-AP + + + + +
EGFP-FRB + + + + +
Rapamycin - - - - +
UL2 (10 μM) - + - + +
UV (10 Min) - - + + +
(kDa)
245
180
130
100
75
60
45
WB: Anti-V5

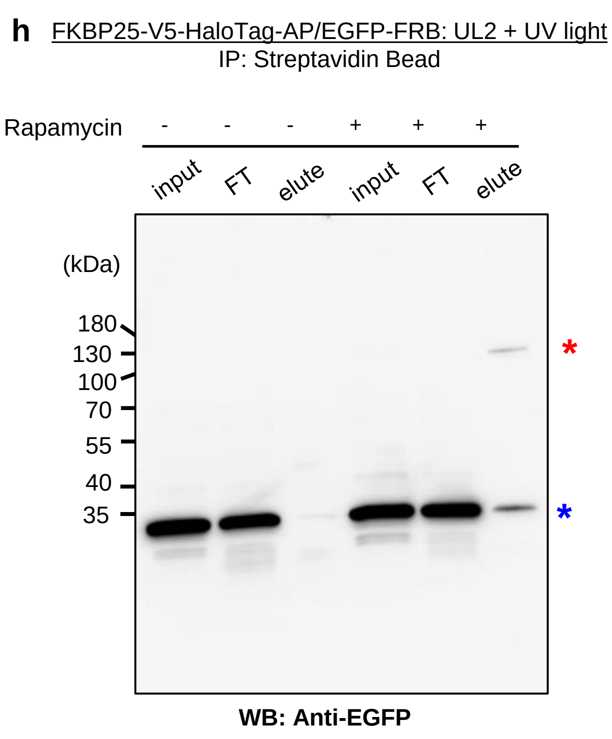

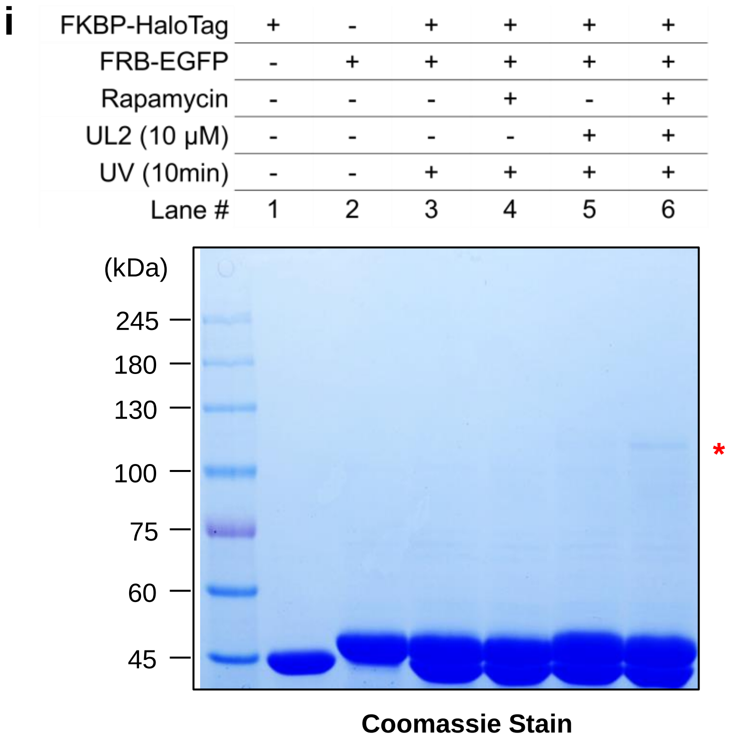

Figure S1 (Related to Figure 1). (**a**) Scheme of proximity photo-crosslinking with a phenylazide conjugated HaloTag ligand. (**b**) Reaction mechanism of UV-activable photo-crosslinkers (i.e., phenylazide, diazirine). (**c**) Chemical structures of UV-activable para-azidophenyl and diazirine conjugated Spotlight probes (UL1, UL2, UL3, UL4). (**d**) Co-crystal structure of HaloTag with UL2. (**e**) Scheme of UL probe-mediated FKBP-FRB photo-crosslinking in the presence of rapamycin and UV light. (**f**) Photo-crosslinking test of EGFP-FRB and FKBP25-V5-HaloTag with various UV-activable photo-crosslinking probes. (**g**) Western blot result of UL2-mediated photo-crosslinking of FKBP25-V5-HaloTag and EGFP-FRB with rapamycin (100 nM) and UV light illumination (10 min) in live cells. The cross-linked EGFP-FRB and FKBP25-V5-HaloTag product is marked with a red asterisk and non-crosslinked FKBP25-V5-HaloTag (**f**) and EGFP-FRB (**g**) are marked with blue asterisks. (**h**) Western blot result of cross-linked FKBP25-V5-HaloTag and EGFP-FRB after immunoprecipitation of FKBP25-V5-HaloTag via its C-terminal-tagged biotinylation acceptor peptide (AP). In the cell lysate, AP was biotinylated by the addition of biotin ligase (BirA), biotin, and ATP, and biotinylated proteins were enriched by streptavidin magnetic beads (see Methods). In the eluted fraction, both the cross-linked EGFP-FRB and FKBP25-V5-HaloTag products (red asterisk) and non-crosslinked EGFP-FRB (blue asterisk) were observed in the rapamycin-treated sample. Anti-EGFP antibody was used for the detection of EGFP-FRB in this blot. (**i**) Purified FKBP12-HaloTag cross-linking with purified FRB-EGFP Coomassie staining result showing that FKBP12-HaloTag cross-linked with FRB-EGFP by UL2 probe in lane 6 with rapamycin pre-treatment. The asterisk indicates the cross-linked band in lane 6.

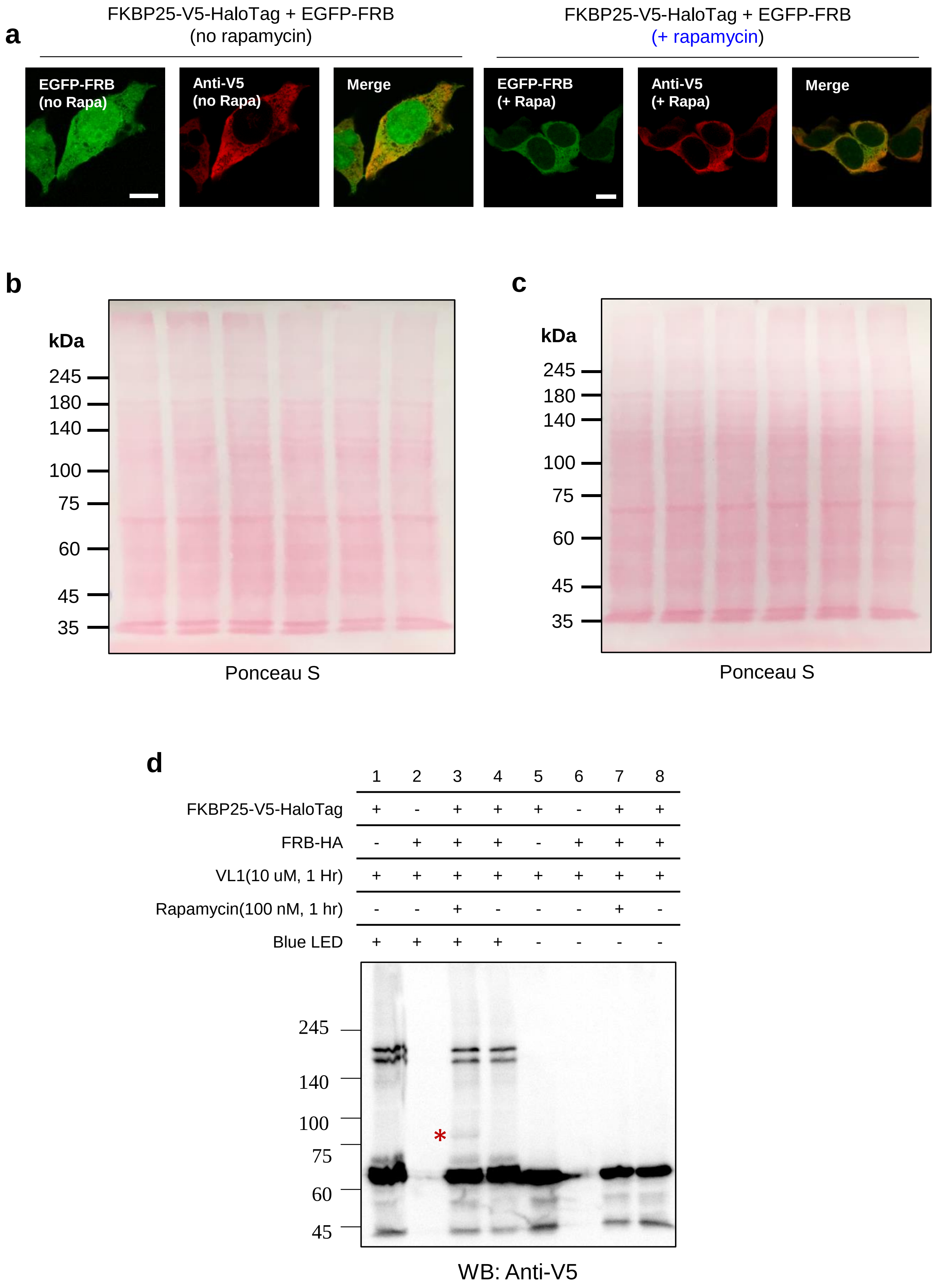

(**Figure S2:** Caption in the next page)

Figure S2 (Related to Figure 1) (**a**) Confocal imaging of the result in HEK293T cells. FKBP25-V5-HaloTag was transfected by Lipofectamine 2000 (Invitrogen) and rapamycin (100 nM) was treated for 1 h. FKBP25-V5-HaloTag was visualized by anti-V5 antibody and anti-mouse Alexa fluor 568 antibody after fixation and permeabilization. The green channel shows the EGFP signal. After rapamycin treatment, the nucleocytoplasmic pattern of EGFP-FRB was changed to a cytosolic localization pattern where FKBP25-V5-HaloTag is localized. Scale bar = 10 μm. (**b**) and (**c**) Ponceau stain of respective western blots in Figure 1e. (**d**) Western blot results of rapamycin-induced HA-FRB cross-linking using FKBP26-HaloTag in HEK293T cells with blue LED light; the cross-linked product is marked with a red asterisk.

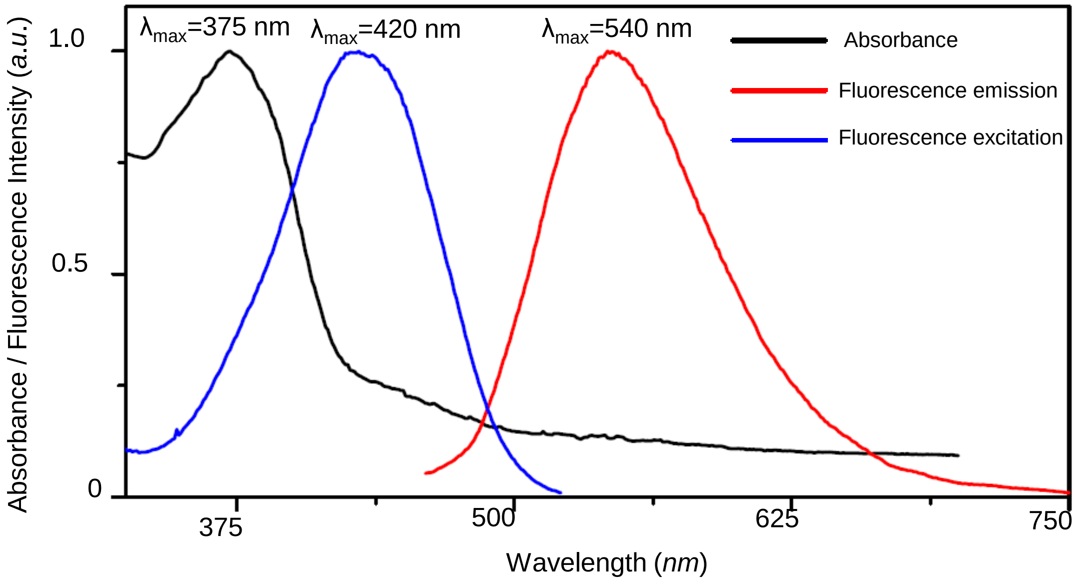

Figure S3. Absorption and fluorescence emission and excitation spectra of **VL1** (10 μM) in PBS buffer (10 mM, pH = 7.4) 10% EtOH.

Photo-crosslinking of **VL1** with BSA

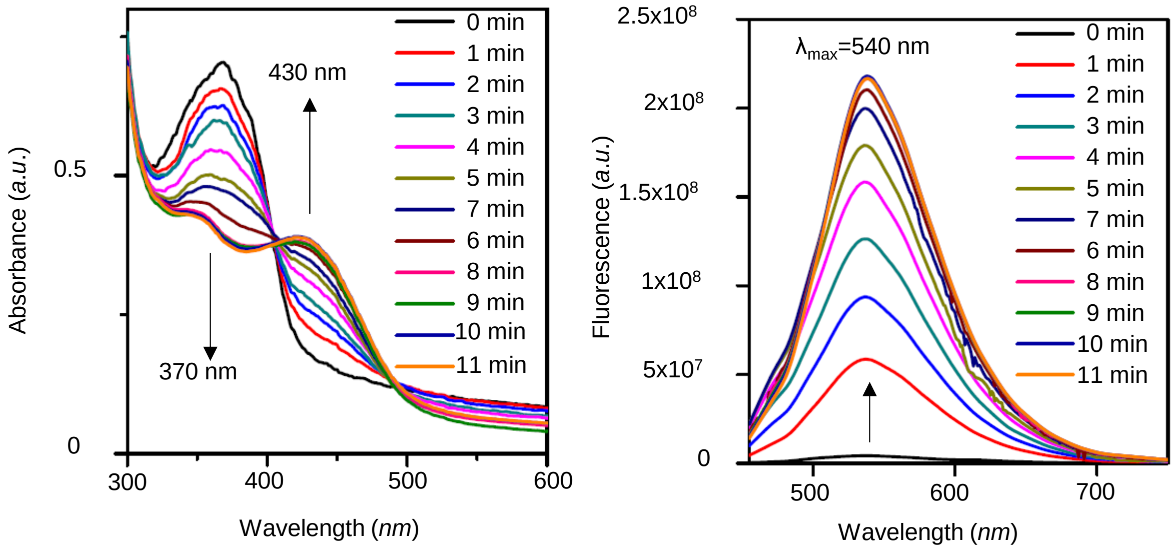

Figure S4. Time-course measurement of the photo-crosslinking reaction of VL1 with BSA. *In vitro* photo-crosslinking reaction with 1 mg/ml BSA and 100 µM, **VL1** under blue LED light (36 W) illumination. The BSA solution (1 mg /ml) was prepared in 10 mM PBS buffer (pH 7.4, 10% EtOH) and then a 10 mM stock of VL1 was added to obtain a final concentration of 100 µM. For the time-course measurement of the photo-crosslinking reaction under blue LED light illumination, a small portion (200 µl) of the VL1 and BSA mixture was taken and kept under blue LED illumination for the designated time; the spectrum was recorded using a 96-well plate with Spectramax 2000 for measurement of the absorbance and fluorescence emission spectra. An excitation wavelength of 420 nm was used for the measurement of the fluorescence emission of photo-activated VL1.

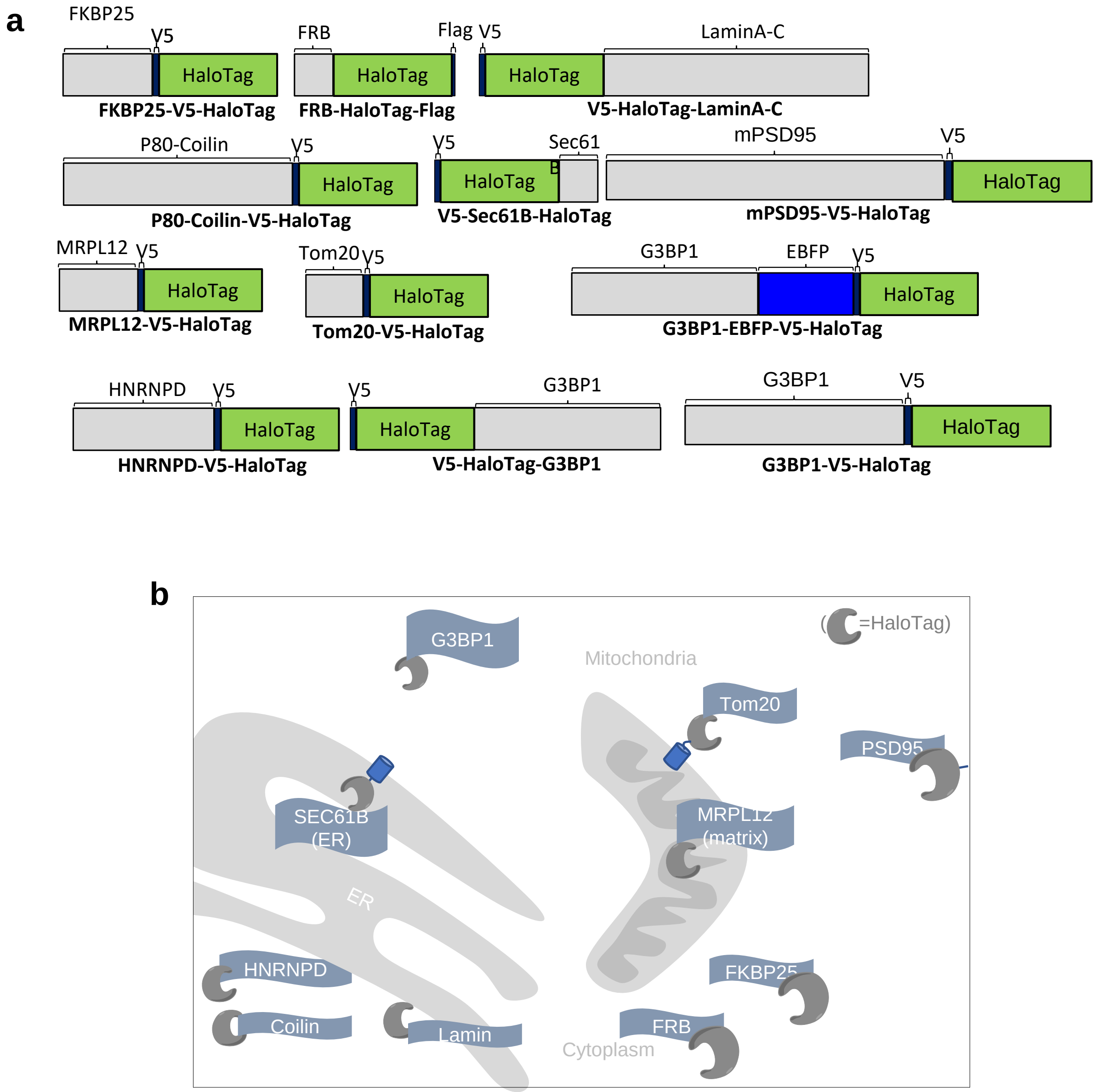

Figure S5 (**Related to Figure 3**) (**a**) Construct map of various HaloTag-V5-POIs. (**b**) Expected subcellular localization of HaloTag-V5-POIs.

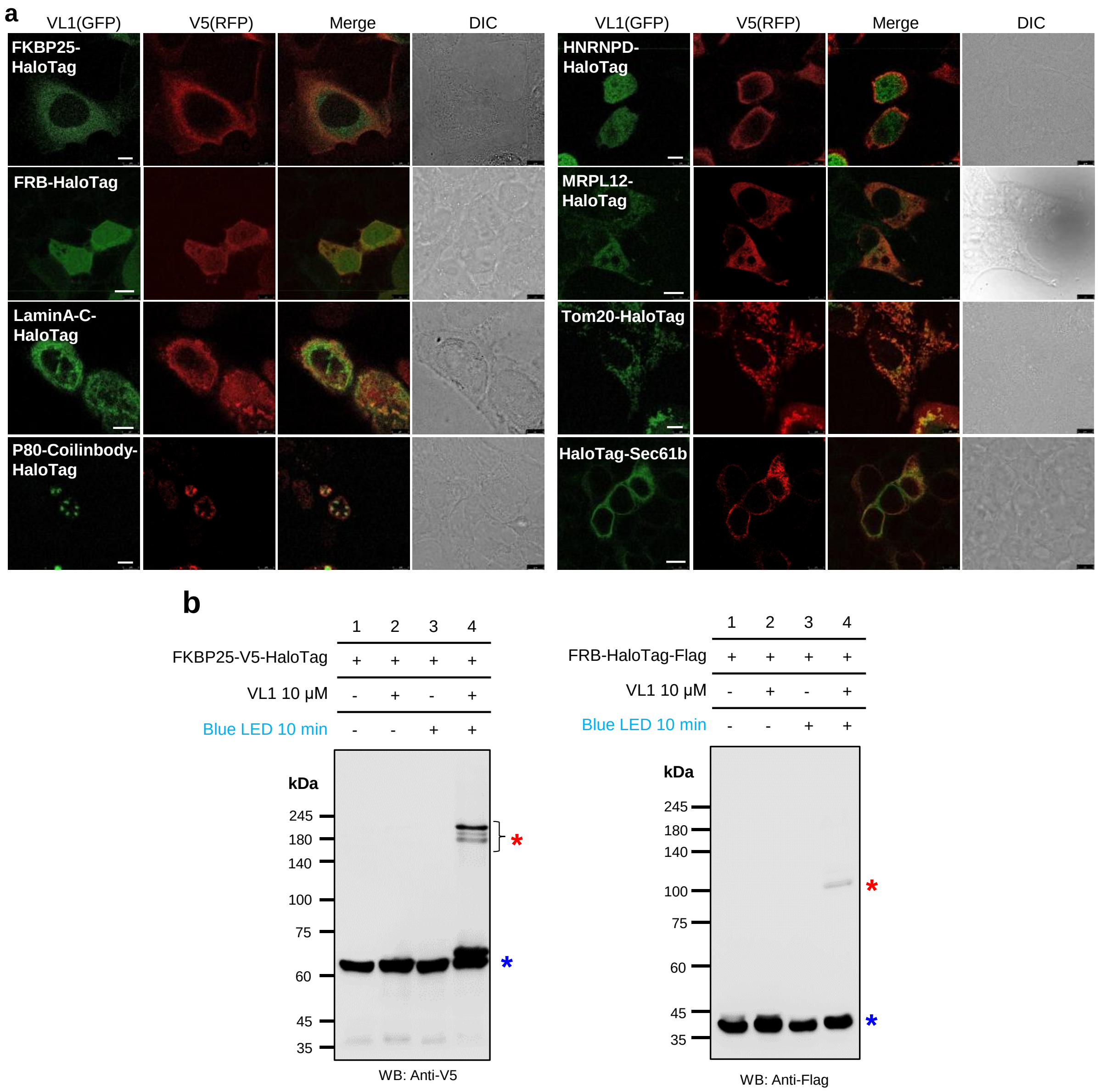

(**Figure S6:** Caption in the next page)

c

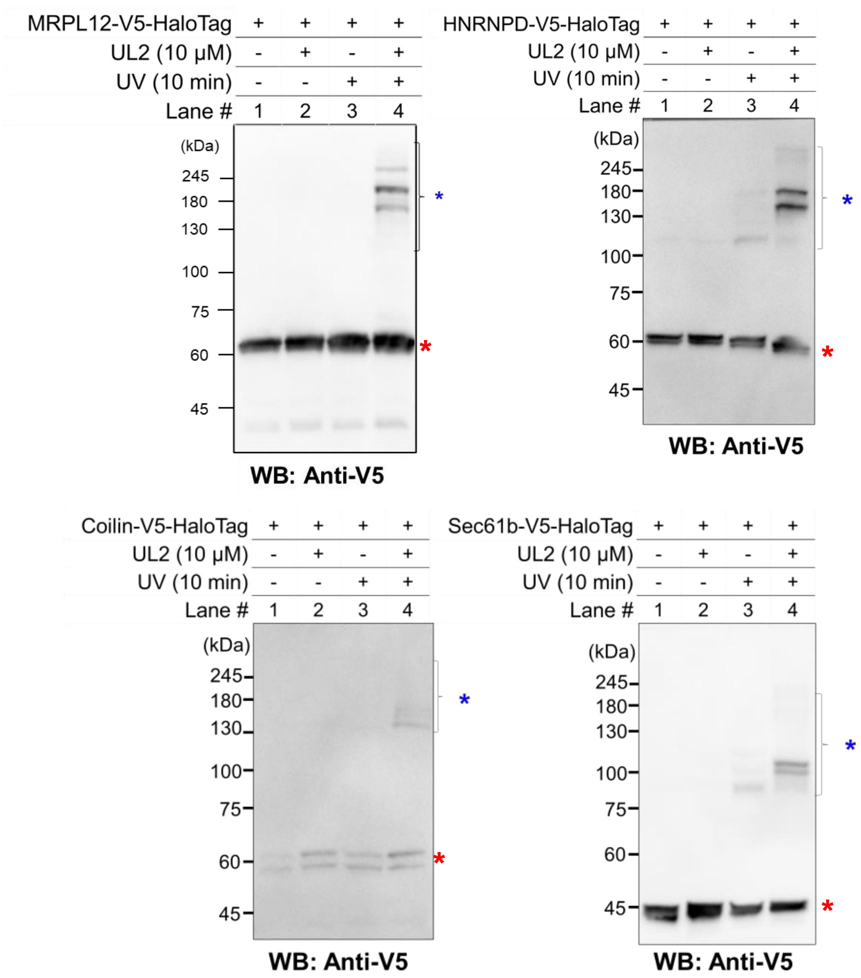

Figure S6 (Related to Figure 3) (**a**) Merged images of VL1 fluorescence (GFP channel) and immunofluorescence image with anti-V5 antibody stain (RFP channel) of HaloTag-V5-POIs. VL1 images are shown in Figure 3. (**b**) Western blot analysis of VL1-mediated photo-crosslinked products of FKBP25-V5-HaloTag and FRB-HaloTag-Flag using anti-V5 antibody and anti-Flag antibody, respectively. Photo-crosslinked products are marked with red asterisks and non-crosslinked POI-HaloTag proteins are marked with blue asterisks. (**c**) Western blot analysis of UL2-mediated photo-crosslinked products of various HaloTag-V5-POIs under UV light illumination (10 min). Photo-crosslinked products are marked with blue asterisks and non-crosslinked POI-HaloTag proteins are marked with red asterisks. For all western blot analyses, negative control samples (no probe and/or no light illumination) were included.

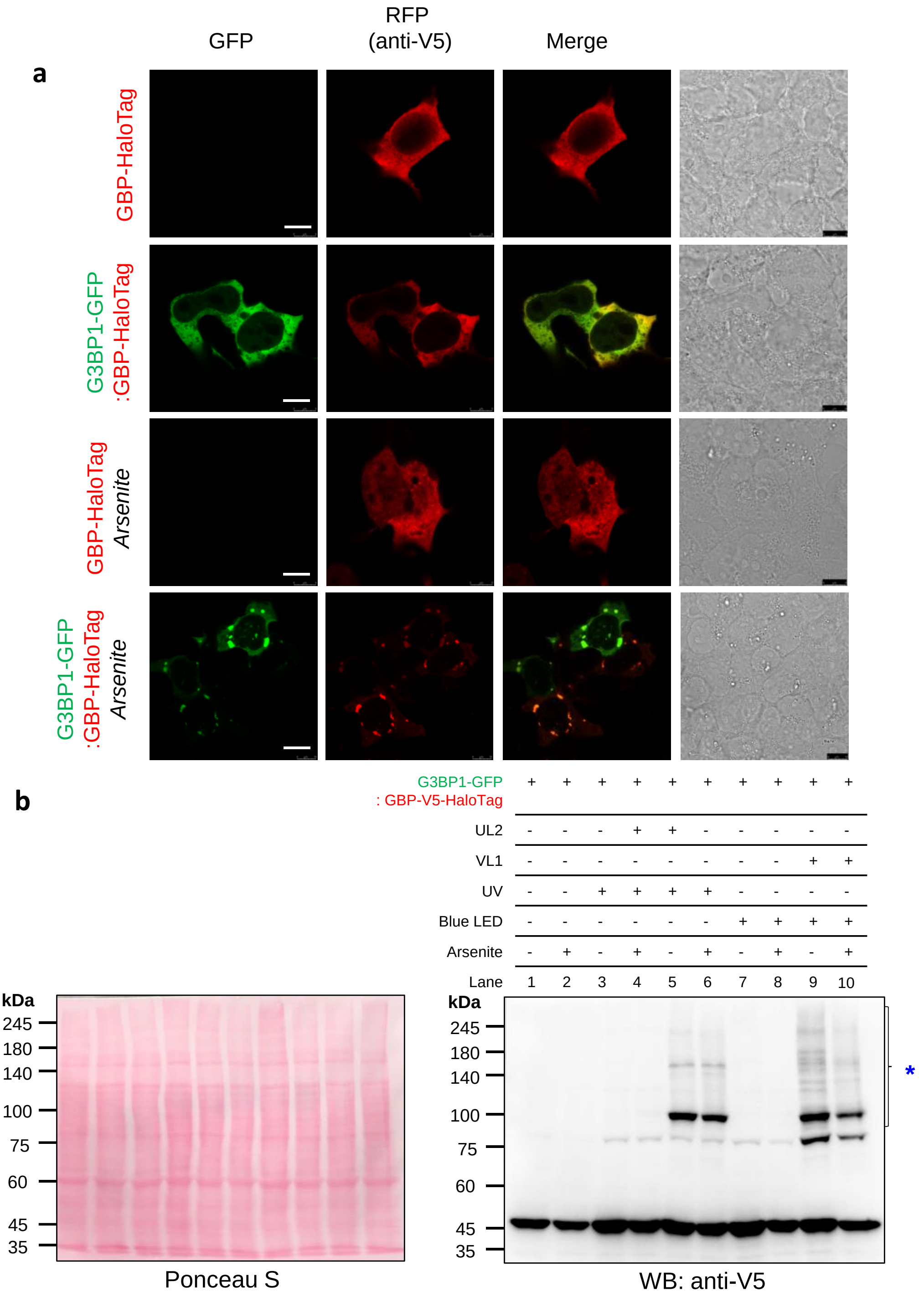

Figure S7 (Related to Figure 4). (**a**) Confocal imaging of G3BP1-GFP with and without GBP-HaloTag under a stress condition (0.5 mM sodium arsenite, 1 h incubation). (**b**) Western blot analysis of G3BP-GFP with and without GBP-HaloTag; cross-linking was performed using UL2 and VL1 by irradiating with UV and blue LED light, respectively.

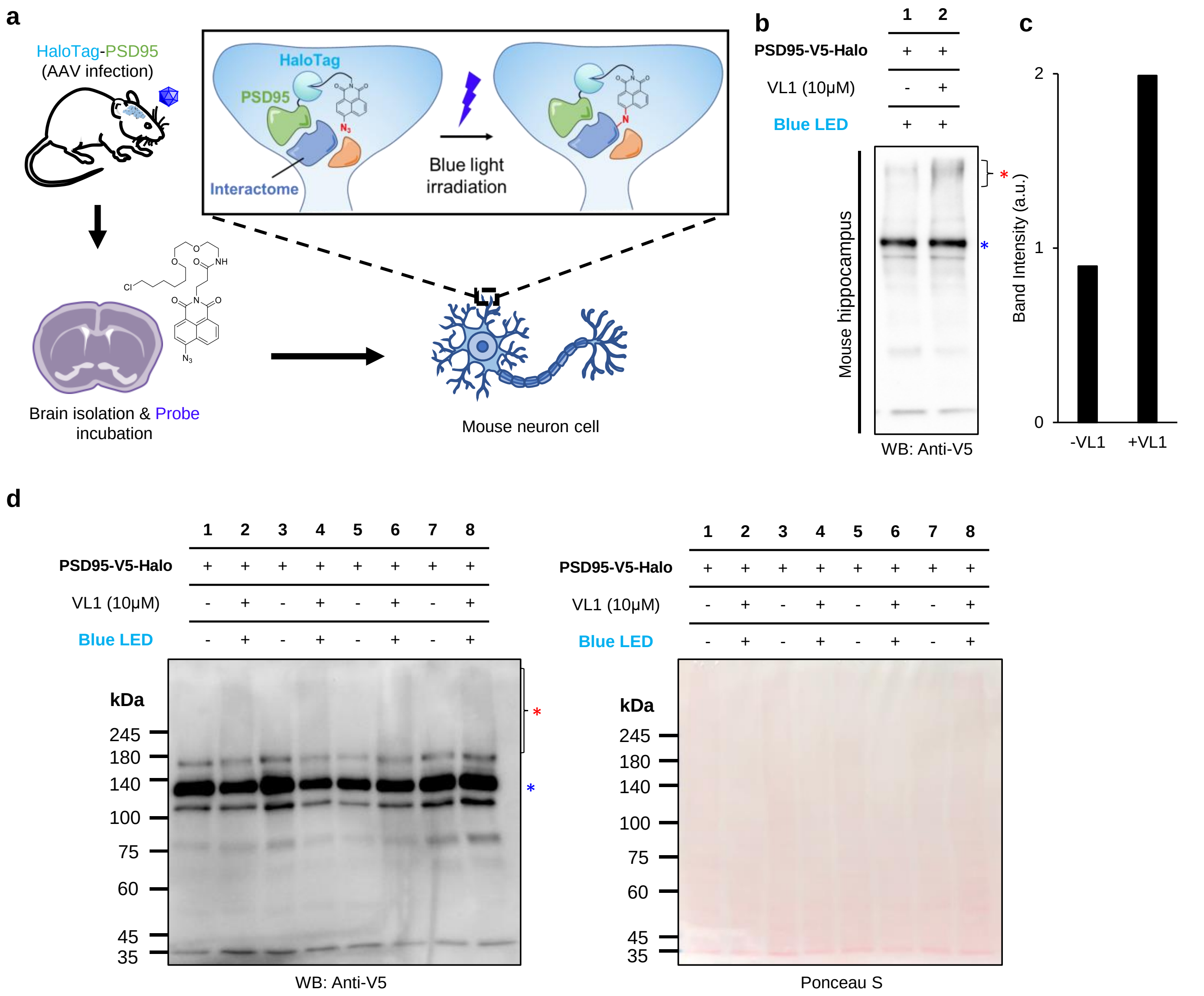

Figure S8 (**a**) Scheme of the VL1-mediated photo-crosslinking reaction of PSD95-HaloTag in mouse brain tissues. (**b**) Western blot analysis of VL1-mediated photo-crosslinked products of PSD95-HaloTag compared by control non-treated with VL1. (**c**) Band intensity of the cross-lin ked bands in Figure S8**b**. (**d**) Western blot analysis of photo-crosslinked products of PSD95-H aloTag compared by the negative control (-VL1, -Blue LED). Photo-crosslinked products are m arked with red asterisks and non-crosslinked POI-HaloTag proteins are marked with blue aste risks.

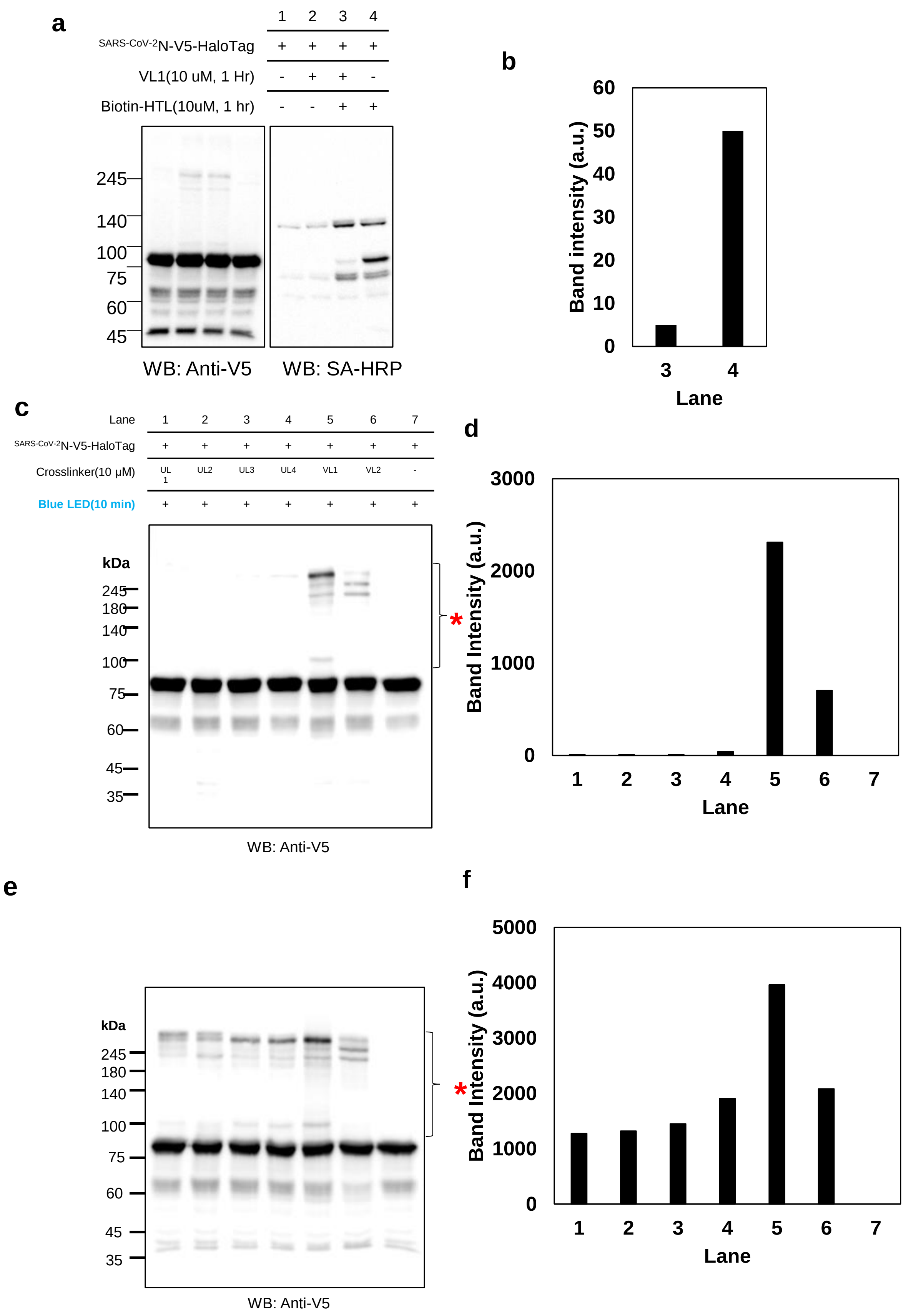

(**Figure S9:** Caption in the next page)

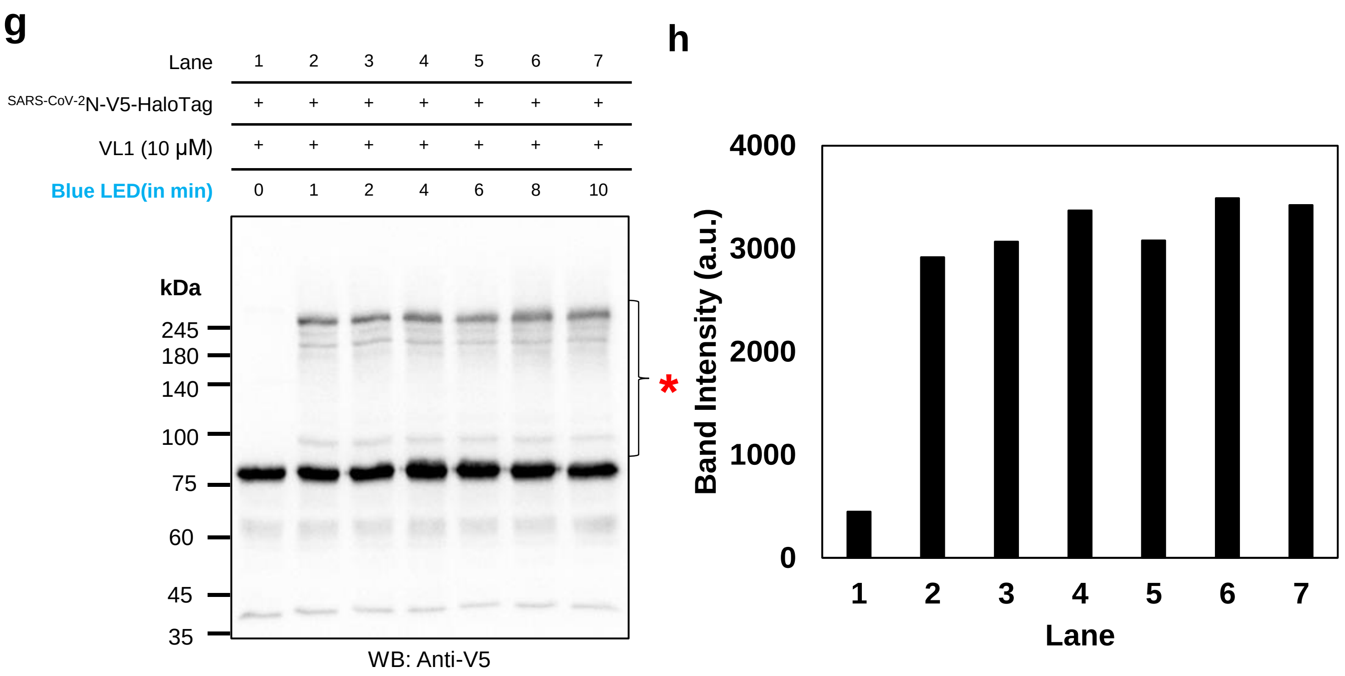

Figure S9 (Related to Figure 5) (**a**) Completion binding assay of VL1 with biotin-HTL on N -HaloTag-expressing HEK293T cells. Biotin-HTL (10 μM) was treated for 60 min after VL1 (10 μM) incubation for 60 min. Biotin-HTL can label unoccupied N-HaloTag protein with or with out prior treatment of VL1, and streptavidin-HRP can detect this population in the western bl ot analysis. (**b**) Bar graph of band intensities of the biotin-HTL-labeled signal of (a). (**c**) Com parison of the photo-crosslinking efficiencies of VL and UL probes for SARS-CoV-2 N-HaloTa g under blue LED light illumination. Anti-V5 antibody was utilized for the detection of N-Ha loTag in this blot. (**d**) Bar graph of band intensities of photo-crosslinked products of (c). (**e**) Comparison of photo-crosslinking efficiencies of VL and UL probes for SARS-CoV-2 N-HaloTa g under UV light illumination. Anti-V5 antibody was utilized for the detection of N-HaloTag in this blot. (**f**) Bar graph of band intensities of photo-crosslinked products of (e). (**g**) Time-course measurement of VL1-mediated photo-crosslinked product of N-HaloTag during blue LE D light illumination (0–10 min). (**h**) Bar graph of band intensities of photo-crosslinked produ cts of (g).

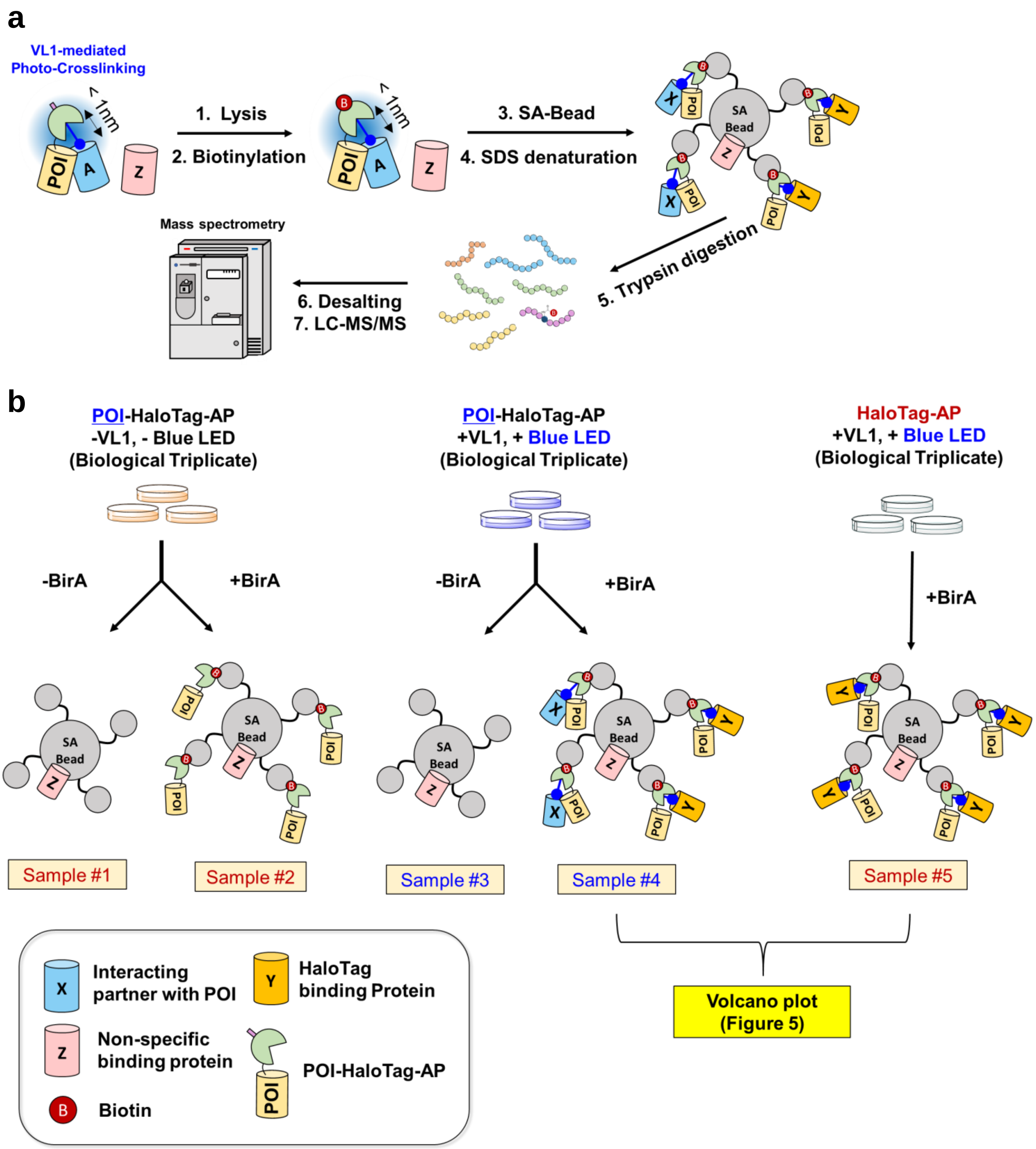

Figure S10 (Related to Figure 5c). (**a**) Workflow of the sample preparation for the mass analysis of Spotlight-cross-linked samples. After VL1-mediated photo-crosslinking of POI-HaloTag, the cells were lysed and POI-HaloTag-AP (or Avitag) was in vitro biotinylated by purified biotin ligase (BirA). Amicon filtration was performed to remove free biotin in the lysate and SA-beads were added to the lysate. Proteins were denatured by the addition of 10% SDS buffer during SA-Bead incubation, which effectively reduces non-specific binding events. Reduction and alkylation of proteins were performed to break disulfide bonds. After trypsin digestion, desalted peptides were analyzed by LC-MS/MS for mapping interactomes. (**b**) A total of six plates of POI-HaloTag-AP (or Avitag)-transfected HEK293T

cells were prepared with a 100 pi dish scale for mass analysis, half of which (three plates) were incubated with VL1, illuminated under blue LED light, and lysed. From each plate, half the volume of the lysates was incubated with BirA for the in vitro biotinylation reaction of AP (or Avitag). All samples, including negative controls (i.e., no VL1 and no light/no biotin ligase), were incubated with SA-bead and SA-bead eluates, trypsin-digested, and analyzed by LC-MS/MS. Proteins exclusively identified in the sample "+VL1, +BirA" were obtained after triple filtration with the results of negative controls. Following this protocol, we performed the mass sampling and LC-MS/MS analysis of N-HaloTag-AP and HaloTag-Avitag.

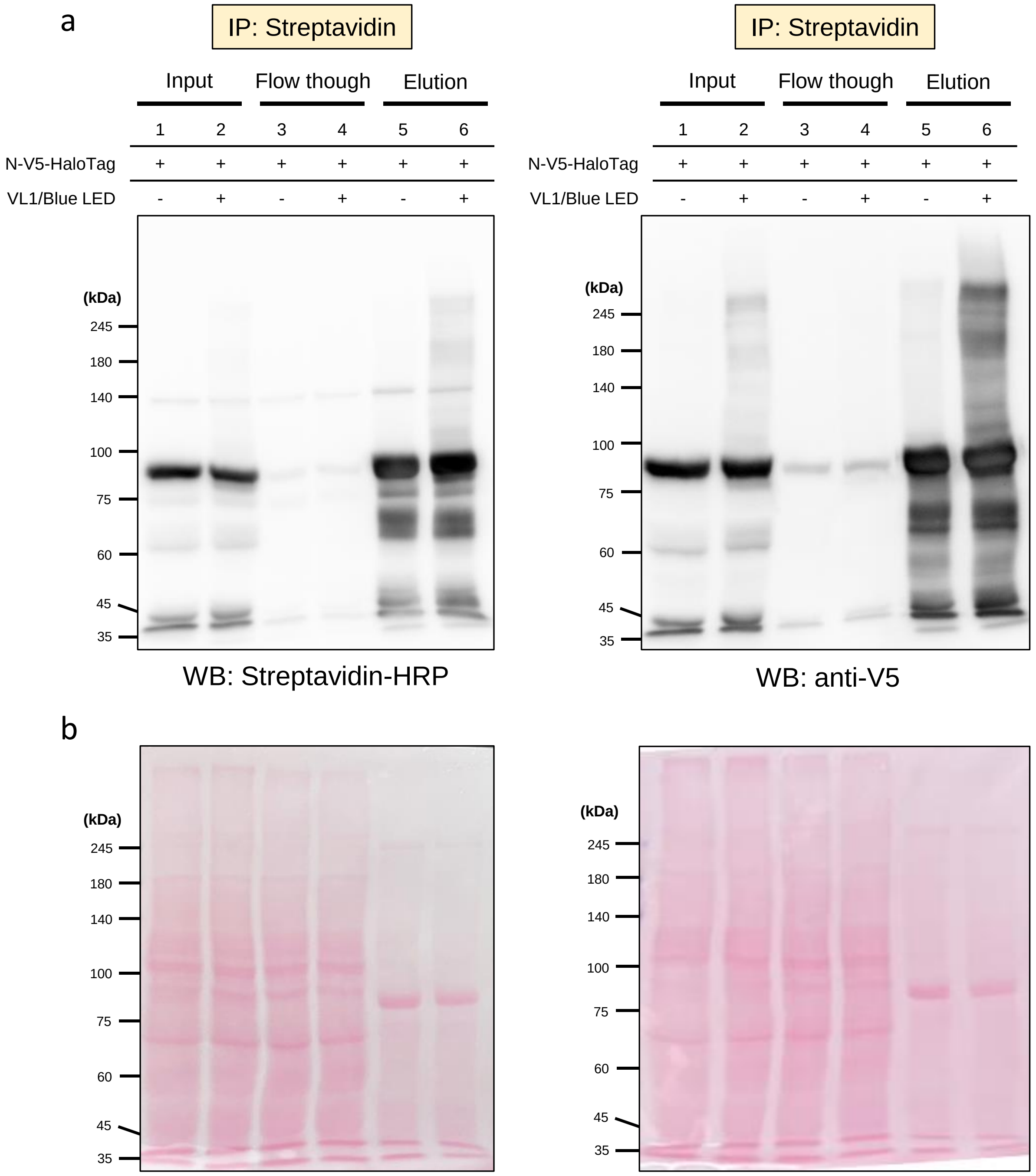

Figure S11 (Related to Figure 5c). (**a**) Western blot results showing enrichment efficiency after in vitro biotinylation. The BirA reaction was carried out using10 μM BirA, 10 μM biotin, 5 mM $MgCl_2$, and 2 mM ATP, and incubated for 2 h. After the reaction, free biotin was removed using Amicon filtration and enrichment was carried out using streptavidin beads (incubated with beads for 1 h, washed with 2% SDS in 1X TBS four times, and eluted with 1X SDS and 1 mM biotin at 95°C for 10 min). (**b**) Ponceau stain of respective western blots in (**a**).

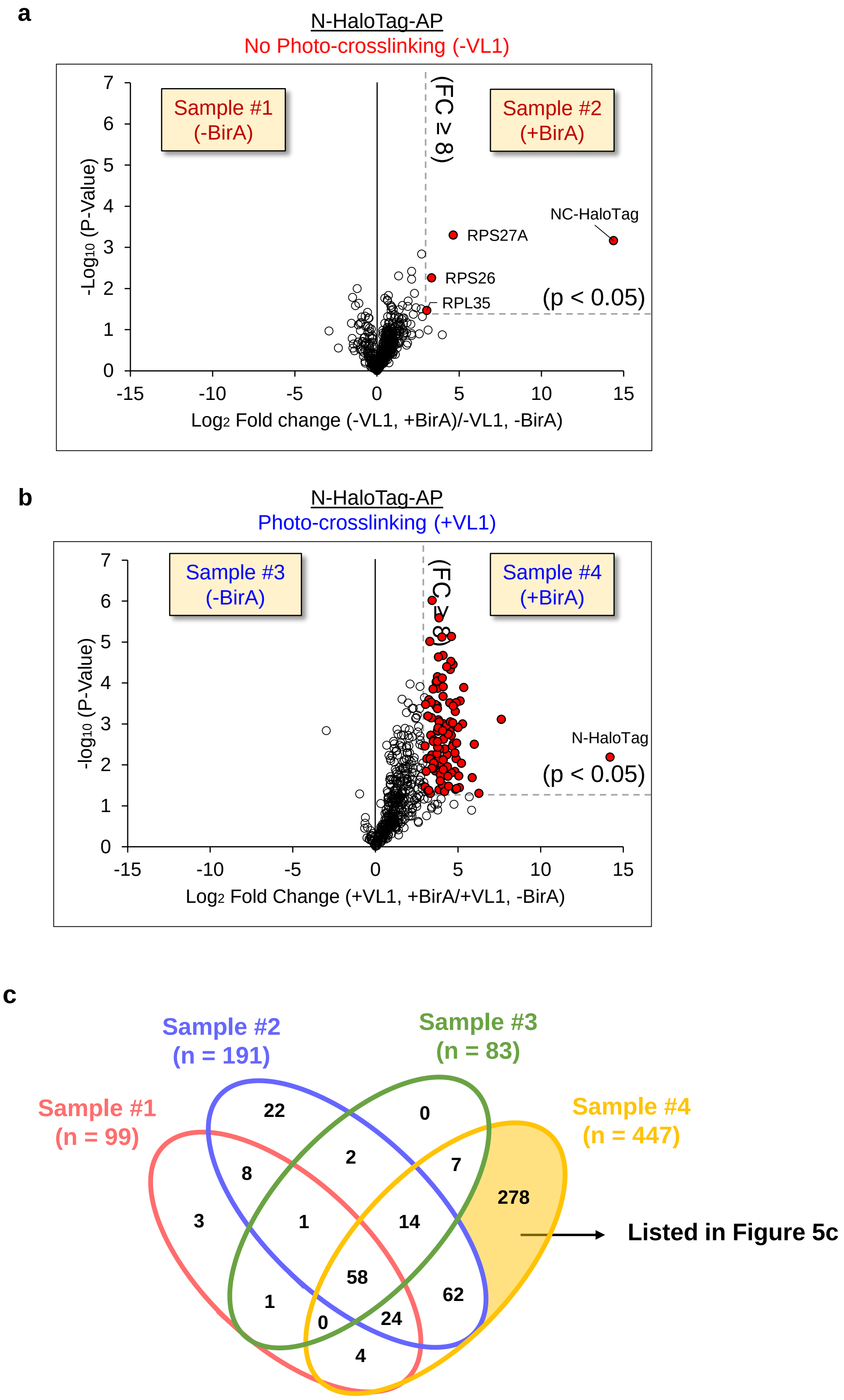

Figure S12 (Related to Figure 5c). (a) Volcano plot showing significant enrichment of four proteins, including the bait protein N-HaloTag-AP, on the no VL1-crosslinked and biotinylated N-HaloTag-AP (-VL1, +BirA: depicted as sample 1 of Figure S9) over the no VL1-crosslinked a nd no biotinylated N-HaloTag-AP (-VL1, -BirA; depicted as sample 2 of Figure S9). FC ≥ 8, $p < 0.05$. See **Supplementary Dataset 1** for details. (b) Volcano plot showing significant enrich

ment of 110 proteins including the bait protein, N-HaloTag-AP, on the VL1-crosslinked and biotinylated N-HaloTag-AP (+VL1, +BirA: depicted as sample 4 of Figure S9) over the VL1-crosslinked and no biotinylated N-HaloTag-AP (+VL1, -BirA; depicted as sample 3 of Figure S9). FC ≥ 8, $p < 0.05$. See **Supplementary Dataset 1** for details. These results indicate that the SDS denaturation washing step is highly efficient to eliminate the non-specific protein binding to bait protein on the streptavidin beads. (c) Venn diagram shows the number of proteins which are the reproducibly observed in triplicates of Sample #1~4. See **Supplementary Dataset 1** for details. 278 proteins are statistically enriched on the N-HaloTag-AP (+VL1, +BirA, Sample #4) colored in yellow and N-interactome was filtered by comparison with the HaloTag-AP (+VL1, +BirA, Sample #5).

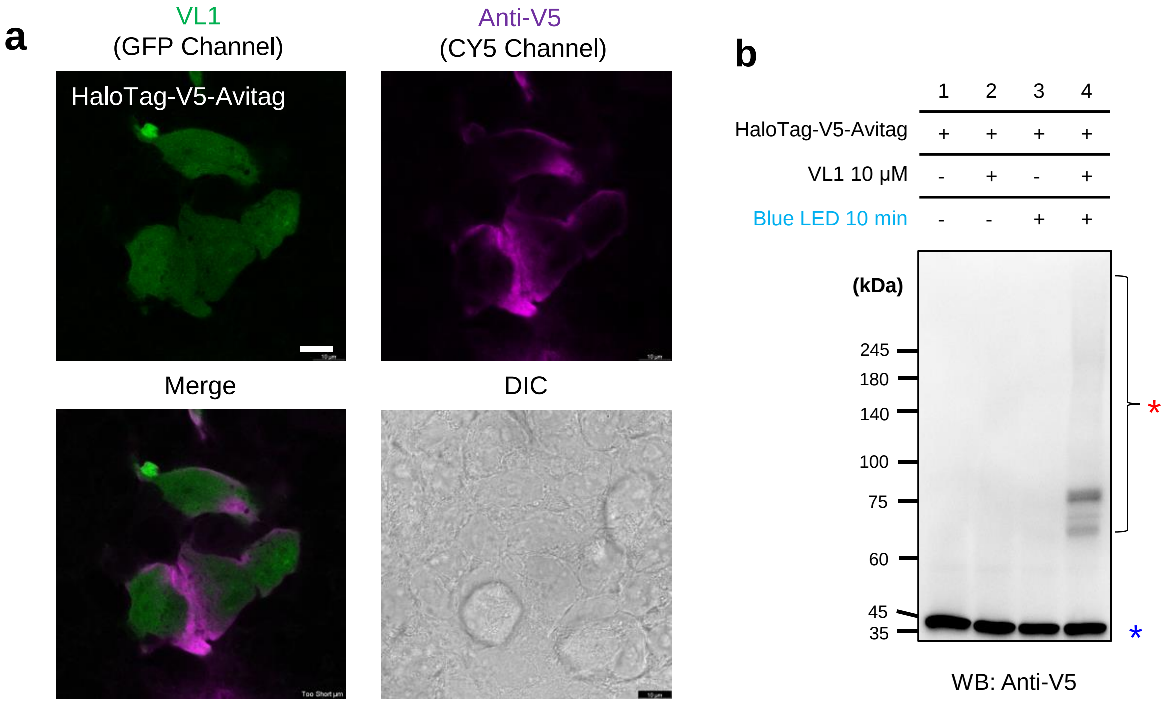

Figure S13 (Related to Figure 5c and d). (**a**) Imaging of HaloTag-V5-AP by anti-V5 antibody (AF647-conjugated, Cy5 channel). HaloTag-targeted VL1 was observed in the GFP channel. Scale bar = 10 μm. (**b**) Western blot analysis of VL1-mediated photo-crosslinked products of HaloTag-V5-AP under blue LED illumination (10 min). Photo-crosslinked products are marked with a red asterisk and non-crosslinked HaloTag-V5-AP proteins are marked with a blue asterisk. For all western blot analyses, negative control samples (no probe and/or no light illumination) were included.

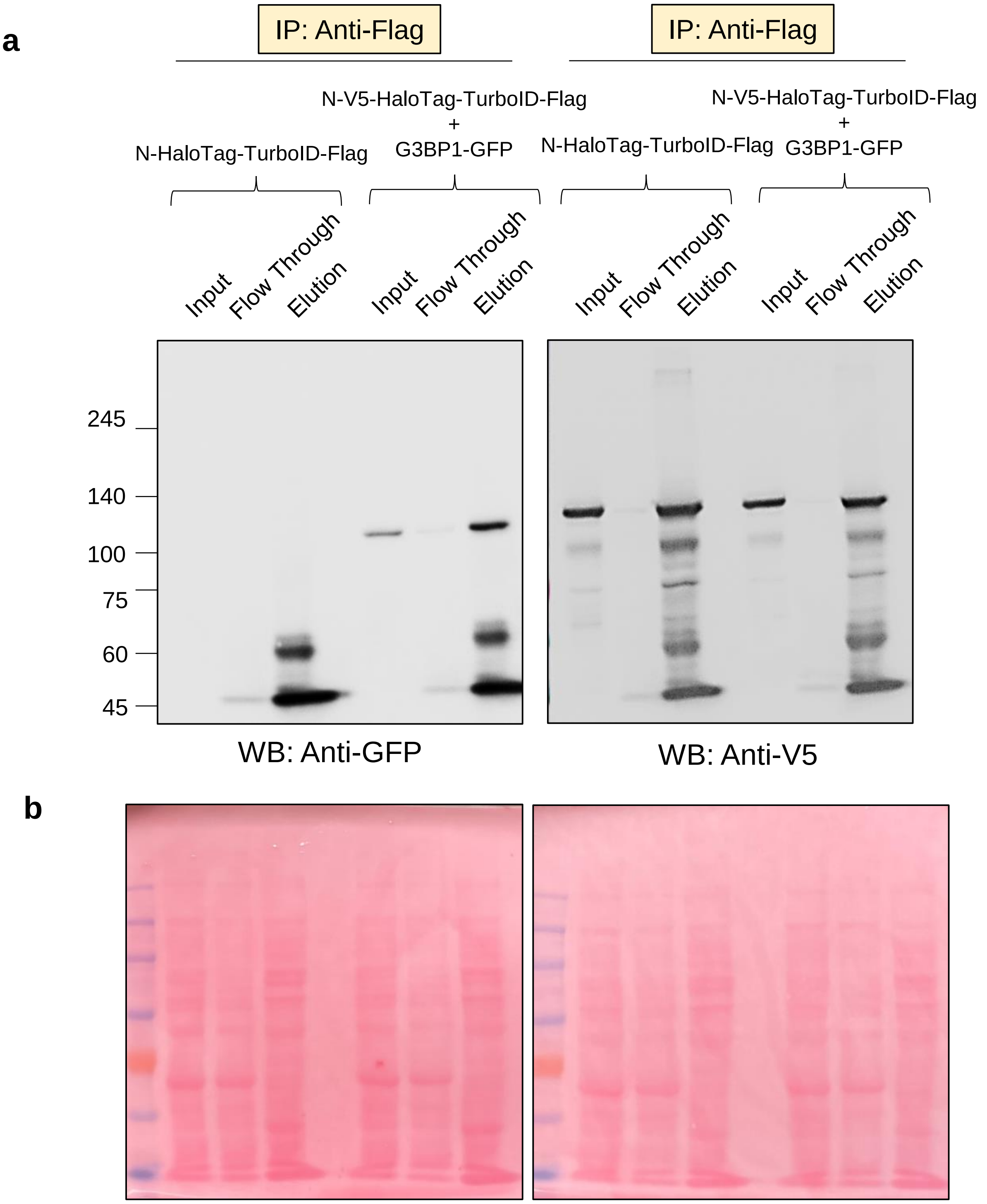

Figure S14 (Related to Figure 5g-j). (**a**) Western blot images showing immunoprecipitation using anti-Flag enrichment for N-HaloTag-TurboID with and without G3BP1-GFP. HEK293T cells were transfected with the POIs. After 24 h of transfection, lysis was performed using 0.5% CHAPS solution and ~0.7 mg of the lysate was incubated with 20 µl of anti-Flag agarose bead (prewashed with 0.5% CHAPS solution) for 2 h, washed three times with 0.5% CHAPS solution, and eluted with 2X SDS loading buffer followed by heating at 95°C for 10 min. (**b**) Ponceau stain of respective western blots in (**a**).

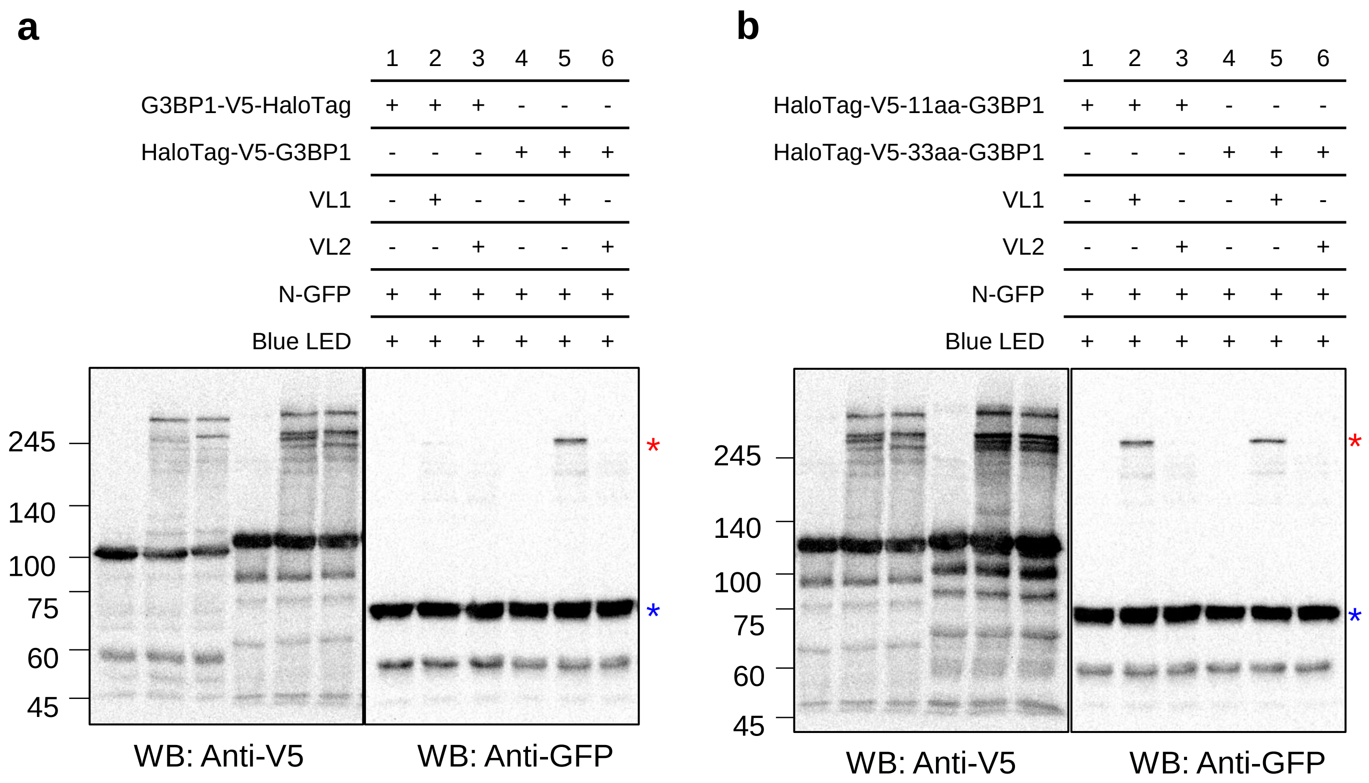

Figure S15 (Related to Figure 5g-j). (**a**) Validation of protein-protein interaction by a VL-crosslinking assay. Either N- or C-terminal G3BP1-conjugated HaloTag constructs (i.e., G3BP1-HaloTag, HaloTag-G3BP1) were co-expressed with G3BP1 in HEK293T cells, and either VL1 or VL2 was incubated at 10 μM for 1 h. No probe-treated samples were included as negative controls. All samples were illuminated by blue LED light (10 min) and lysed for western blot analysis using anti-V5 antibody (left) and anti-GFP antibody (right). In the result of the anti-V5 western blot, both VL1 and VL2 generated cross-linked products of G3BP1-HaloTag and HaloTag-G3BP1 proteins. However, in the anti-GFP western blot result, only VL1 generated the N-GFP cross-linked product on the HaloTag-G3BP1 protein whose HaloTag is conjugated at the N-terminus of G3BP1. (**b**) Either a short linker (11 aa) or long linker (33 aa) construct of HaloTag-G3BP1 (i.e., HaloTag-11aa-G3BP1 HaloTag-33aa-G3BP1) was co-expressed with N-GFP in HEK293T cells, and incubated with either VL1 or VL2 at 10 μM for 1 h. No probe-treated samples were included as negative controls. All of the samples were illuminated by blue LED light (10 min) and lysed for western blot analysis using anti-V5 antibody (left) and anti-GFP antibody (right). In the result of anti-V5 western blot, both VL1 and VL2 generated cross-linked products of HaloTag-11aa-G3BP1 and HaloTag-33aa-G3BP1 proteins. In the anti-GFP western blot result, both HaloTag-11aa-G3BP1 and HaloTag-33aa-G3BP1 showed VL1-mediated N-GFP cross-linked products. Photo-crosslinked products with VL1 are marked with red asterisks and non-crosslinked HaloTag-V5-AP proteins are marked with blue asterisks.

Materials and Methods

Plasmids and Cloning. Genes were cloned into the specified vectors using standard enzymatic restriction digest and ligation with T4 DNA ligase. To generate constructs where short tags (e.g., V5 epitope or AviTag) or signal sequences were appended to the protein, the tag was included in the primers used to PCR-amplify the gene. PCR products were digested with restriction enzymes and ligated into cut vectors (e.g., pcDNA3, pCDNA5, pDisplay, pET21a and pH6HTN). In all cases, the CMV promoter was used for expression in mammalian cells. **Table S1** below summarizes the genetic constructs cloned and used for this study.

Protein purification. Gene encoding protein was amplified using PCR and cloned into modified pET21a or pH6HTN vector with histidine tag (6X His). The genes (FKBP12-EGFP, FRB-V5-HaloTag, and BirA-His6 in Table S1) were transformed into expressed in BL21 (DE3) Escherichia coli cells following induction at 42ºC for 30 sec and BL21 was cultured on ampicillin treated agar plate. A colony was picked into 5ml ampicillin containing LB-Broth overnight, and 1ml LB was transferred to 1L new LB broth. After reaching to 0.5 optical density, cells were treated with 0.25 mM IPTG and cultured at 18 ºC for 24 hr. Cells were harvested 24 hours post-induction, lysed by B-per buffer (Invitrogen), centrifugation, and protein was purified by Ni^{2+}-NTA chromatography. The eluted protein was finally concentrated, and free imidazole ring was removed by using Amicon Ultra-15 centrifugal filter (Mw. 10 kDa cutoff, Millipore) and flash-frozen in liquid nitrogen for storage. For crystallization, HaloTag proteins were purified as described previously[1]. For the formation of HaloTag complexed with VL1 and UL2 ligands, purified proteins were mixed with 3-fold molar excess of VL1 and UL2, respectively. After incubating for 3 hours on ice, proteins were injected onto a size-exclusion column (GE healthcare, superdex200 16/600) equilibrated with 25 mM Tris pH 7.5, 150 mM NaCl, 5 mM DTT. Finally, the eluted proteins were concentrated to 16 mg/ml and stored at -80°C.

Crystal structure determination. The Halotag proteins bound to VL1 or UL2 were crystallized by the hanging-drop method by mixing 1 µl protein and 1 µl of crystallization solution. The crystallization condition of the HaloTag-VL1 complex was 1.2 M Ammonium sulfate, 0.1 M citric acid pH 5.3, and 1% MPD at room temperature, and HaloTag-UL2 complex were crystallized under buffer containing 25% PEG 8K, 0.1 M MES pH 6.5, 0.2 M Na-acetate at room temperature. The crystals were soaked into a cryoprotectant solution consisting of crystallization buffer plus 30% glycerol, and flash frozen in liquid nitrogen. X-ray diffraction data were collected at the beamline 7A of the Pohang Accelerator Laboratory (PAL), and processed by HKL2000 program[2]. The crystal structure was solved by the molecular replacement by Phenix[3] using apo-HaloTag (PDB accession code: 5Y2X) as a search model. Model building and refinement were carried out using Coot[4] and Phenix[3], respectively. Data collection and refinement statistics are summarized in Table S2.

General Transfection Protocol. For transfection cells were cultured in DMEM (hyclone, SH30243) supplemented with 10% FBS, 2 mM L-glutamine, 50 units/mL penicillin, and 50 µg/ml streptomycin at 37°C under 5% CO2, for 12 well plate at 60-70% confluency, 1000 ng plasmid DNA was mixed with 2µg of Polyethylenimine (PEI, Polysciences, 23966) using 100 uL no-FBS, DMEM and added to the well, after 2-3 hours of addition, media was changed to full media described above and transfected cells were used for imaging or crosslinking experiments after 20-24 hrs of transfection.

General Imaging Protocol. For imaging experiments, HEK-AD or HEK293T cells were cultured in DMEM supplemented with 10% FBS, 2 mM L-glutamine, 50 units/mL penicillin, and 50 μg/ml streptomycin at 37°C under 5% CO2. After 24 hr transfection, 10μM **VL1** in DMEM was treated into cells for 1 hr. Then, cells were washed by DPBS (thermofisher, 21300) 3 times and cells were fixed with 4% paraformaldehyde solution (chembio, CBPF-9004) in DPBS at room temperature for 15 min. Cells were then washed with DPBS two times and permeabilized with chilled methanol at −20°C for 5 min. Cells were washed again two times with DPBS and blocked for 30 min with 2% BSA (milipore, 82-100-6) in DPBS ("blocking buffer") at room temperature. To detect HaloTag fusion protein expression, cells were incubated with mouse anti-V5 antibody (Invitrogen, cat. no. R960-25, 1:5000 dilution) for 1 h at room temperature. After washing four times with TBST each 5 min, cells were simultaneously incubated with secondary Alexa Fluor 568-goat anti-mouse IgG (Invitrogen, cat. no. A-11004, 1:1000 dilution) for 30 min at room temperature.

General Protocol for Visualizing Crosslinking on Western blot. For crosslinking experiments, HEK-293T cells were cultured in DMEM supplemented with 10% FBS, 2 mM L-glutamine, 50 units/mL penicillin, and 50 μg/ml streptomycin at 37°C under 5% CO2. After 24 hr transfection, 10μM **VL1** in DMEM was treated into cells for 1 hr. Then, cells were washed by DPBS 3 times and subjected to Blue LED light for 10 min and RIPA lysis buffer (Elpis biotech, EBA-1149) was added after removal of DPBS. Lysis was performed for 30 min at 4°C Then, sample was loaded into 6% SDS-PAGE gel and run at 150V for 60 min. After separation, proteins on gel was transferred to nitrocellulose membrane at 400 mA for 90 min. Protein loading level was checked by ponceau staining and ponceau was removed by 1xTBST buffer. Blocking was performed with 2% skim milk in TBST for 1 hr. Blocking solution was replaced with primary antibody in 2% skim milk and incubated for 1 hr. After 4 times (each for 5 min) of 1xTBST buffer washing, membrane was incubated with secondary antibody in 2% skim milk in TBST for 30 min. After 4 times washing with 1xTBST buffer, developing was performed with ECL kit (Biorad, 1705061) and image was taken by Gel doc machine (Genesys)

PSD95-HaloTag Expression in Mouse Brain and Proximity Crosslinking with VL1.

Adenoassociated virus (AAV)vector encoding mouse PSD95-V5-Halotag was generated by amplification of the full-length region (Genbank accession number: NM_019621.1) by PCR and subsequently subcloned into the pAAV-T2A-tdTomato vector (a gift from Dr. Hailan Hu, Zhejiang University, China) at XbaI and BamHI sites.[5] AAV packing plasmids (pAAV-PSD95-T2A-tdTomato, pHelper and AAV1.0 serotype 2/9) were transfected into HEK 293T cells. 72-108 hours after transfection, cells were harvested by addition of 0.5 M EDTA to the media and collected by centrifugation. These cells were subjected to four freeze-thaw cycles (7 minutes in ethanol/dry ice and 5 minutes in 37°C water) and centrifuged to divide supernatants from lysates. Collected supernatants were mixed and incubated with a solution containing 40% polyethylene glycol and 2.5 M NaCl on ice for 1 hour and centrifuged at 2000 rcf for 30 minutes. The pellets were resuspended in HEPES buffer (20 mM HEPES pH 8.0, 115 mM NaCl, 1.2 mM $CaCl_2$, 1.2 mM $MgCl_2$, 2.4 mM KH_2PO_4, and then mixed with chloroform by vortexing for 2 minutes and centrifuged at 400 rcf for 10 minutes. The supernatant was transferred into the Amicon Ultra Centrifugal Filters (0.5 ml, 3K MWCO; Millipore) to concentrate the viruses. AAV virus titer was determined by RT-PCR, and the virus concentrated at 1 X 1010–1011 infectious units/ml was used in the experiments. 4–week-old C57BL/6N male mice were anesthetized by intraperitoneal injection of avertin solution (2% 2,2,2-tribromoethanol dissolved in tert-amylalcohol), and fixed in a stereotaxic apparatus. Viruses were injected into hippocampus of mice with a Hamilton syringe using a Nanoliter 2010 Injector (World Precision Instruments) as following coordination: anteroposterior (AP), -2.1 mm; medial–lateral (ML), ± 1.2 mm; and dorsal–ventral (DV), 2.2 mm from bregma. After surgery, injected mouse was returned to its home cage for recovery. Hippocampi dissected from brains of injected mice were minced and incubated in 10 µM of VL1 for an hour. Tissues were washed by PBS twice and then, irradiated to Blue LED light for 30 min. After crosslinking with VL1, dissected hippocampi were homogenized in ice-cold homogenization buffer (320 mM sucrose, 5 mM HEPES-NaOH (pH 7.5), 1 mM EDTA, 0.2 mM PMSF, 1 µg/ml aprotinin, 1 µg/ml leupeptin, 1 µg/ml pepstatin, and 1 mM Na_3VO_4.) The homogenized sample was centrifuged at 1000 x g for 10 minutes, and the supernatant was centrifuged at 15,000 x g for 20 minutes. The pellets were resuspended in RIPA lysis buffer and centrifuged at 15,000 x g for 10 minutes. After concentration measurement by BCA assay (thermofisher, 23225), immunoblotting was performed as described above.

Mass sample preparation of photo-crosslinked N-HaloTag For the mass analysis of the crosslinked product HEK293T cells were grown as triplicate in 100π dish until confluency was 60-70% then 8,000 ng of plasmid DNA was transfected using PEI transfection reagent and media was changed to full media after 3 hours, after 24 hours of transfection 10 μM VL1 was incubated for 1 hour and washed 3 times with cold DPBS before crosslinking in Blue LED for 10 min, cells were scrapped in DPBS made pellet and lysed by RIPA buffer, then lysate was subjected to in-vitro biotinylation using recombinant wild type BirA solution (10 μM BirA, 10μM biotin, 200μM ATP, and 500μM $MgCl_2$) for 12 hours at room temperature. For removing free biotin, lysates were loaded on amicon filter and centrifugated at 12,000 x g for 4 x 15 min. 1xTBS buffer was added into concentrated lysate up to 400 μL and then, 100 μL of washed streptavidin beads were added. After 10 min incubation, 2ml SDS in TBS was added up to 2~10% final concentration and incubated for an hour. Beads were washed by 2% SDS in TBS buffer 4 times and incubated with 10mM DTT in 50mM ABC buffer at 37℃ for an hour. Alkylation was performed by 55mM IAM in ABC buffer at 37℃ in dark condition. After an hour incubation, solution was replaced by 200 μL of trypsin solution (2 μg trypsin, 1mM $CaCl_2$ in 50mM ABC buffer) and mixed at 37℃ for 12 hours. Then, supernatant was desalted using zip-tip (thermofisher, 87784), eluted fractions were dried using speed vac and kept in deep freezer until loaded on LC-MS/MS.

LC-MS/MS analysis Peptides were analyzed by Thermo-Scientific® Q Exactive Plus equipped with a nanoelectrospray ion source. A C18 reverse-phase HPLC column (500 mm × 75 μm i.d.) was used to separate the peptide mixture using a 2.4–17.6% acetonitrile/0.1% formic acid gradient for 120 min at a flow rate of 300 nL/min. For MS/MS analysis, precursor ion scan MS spectra (m/z 350 - 2000) were acquired using the Orbitrap spectrometer at a resolution of 70 K at 200 m/z with an internal lock mass. Resolution of 17,500 at m/z 200 for HCD spectra was set and the 15 most intensive ions were isolated and fragmented by higher energy collisional dissociation (HCD).

For protein identification, Scaffold (version 4.11.0, Proteome Software Inc., Portland, OR) was used to validate MS/MS based peptide and protein identifications. Peptide identifications were accepted if they could be established at greater than 95.0% probability by the Scaffold Local FDR algorithm. Protein identifications were accepted if they could be established at greater t han 99.0% probability and contained at least 2 identified peptide. Protein probabilities were assigned by the Protein Prophet algorithm.[6] Proteins that contained similar peptides and cou ld not be differentiated based on MS/MS analysis alone were grouped to satisfy the principles of parsimony.

In database searching, tandem mass spectra were extracted by Proteome Discoverer (version 2.2, Thermo Fisher Scientific, San Jose, CA). All MS/MS samples were analyzed using Sequest (XCorr Only). Sequest was set up to search Homo sapiens protein sequence database (42230 e ntries, UniProt (http://www.uniprot.org/)) assuming digestion with trypsin. Sequest was search ed with a fragment ion mass tolerance of 0.80 Da and a parent ion tolerance of 10.0 PPM. C arbamidomethyl of cysteine was specified in Sequest as a fixed modification. Oxidation of me thionine and acetyl of the n-terminus were specified in Sequest as variable modifications. For the volcano plot analysis (**Figure 5d**, **Figure S10a,b**), five multiple imputation of missing value s were followed via panda-view software[7] (see Supplementary Dataset 1).

Probe Synthesis

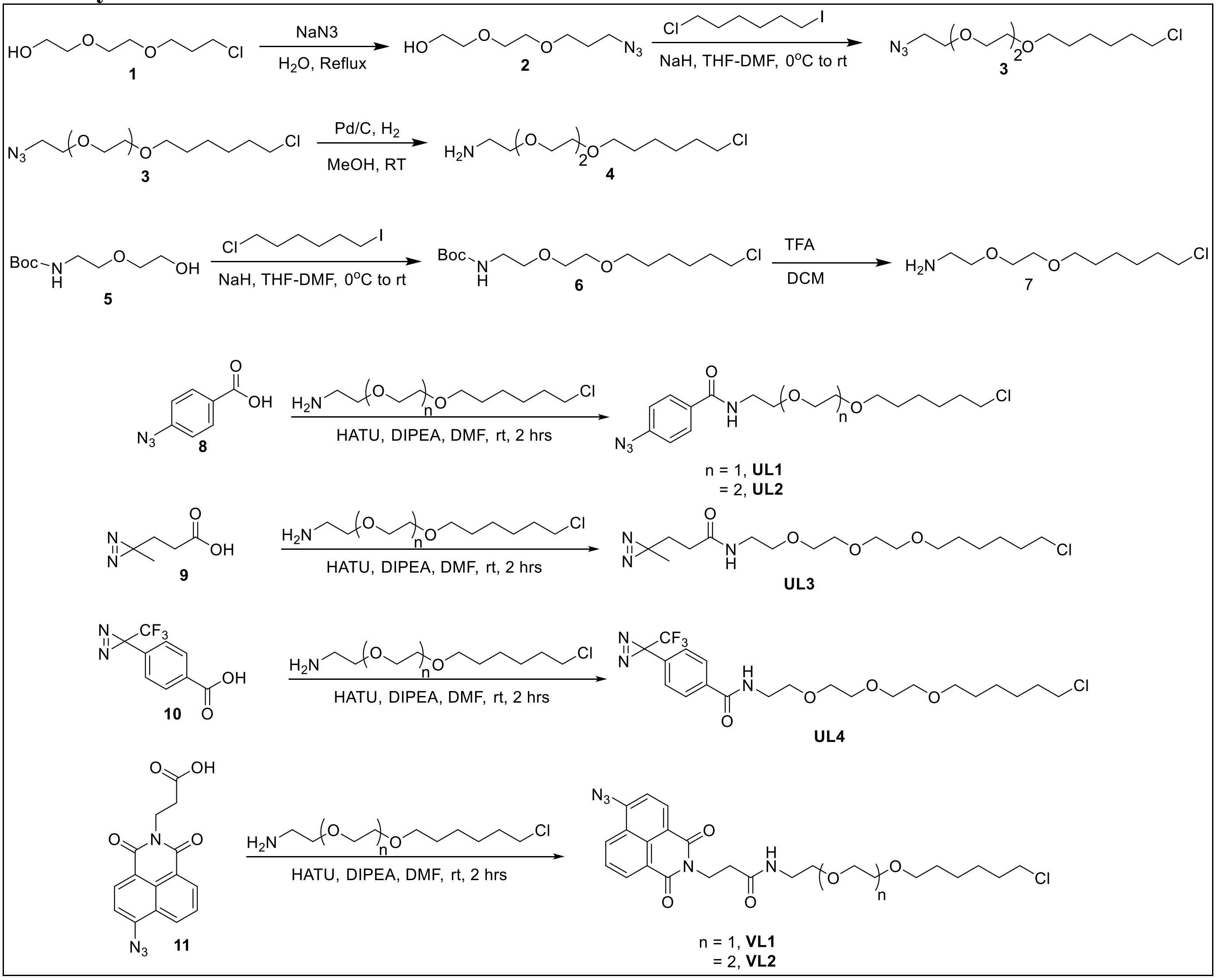

Scheme S1. Synthetic Scheme of **UL1-UL4** and **VL1-VL2**.

Synthesis of 1-(2-(2-(2-azidoethoxy)ethoxy)ethoxy)-6-chlorohexane To a solution of 2-(2-(2-azidoethoxy)ethoxy)ethanol (17.5 mg, 0.1mmol)[8] in DMF at 0 °C was added NaH (6 mg, 0.15 mmol). After stirring for 30 minutes at 0 °C, 1-chloro-6-iodohexane (18.6 μL, 0.12 mmol) was added. The mixture was stirred for 1h, and warmed to r.t., and stirred for 2 h. Ammonium chloride was introduced at 0 °C into the reaction mixture, extracted with diethyl ether (10 mL × 3), washed with saturated brine (10 mL), dried over anhydrous Na_2SO_4 and filtered, and concentrated in vacuo. The crude material was purified by flash chromatography on silica gel to afford compound 1b (12 mg, yield : 40%). ^{1}H NMR (400 MHz, Chloroform-*d*) δ 3.67 (m, 6H), 3.60 – 3.57 (m, 2H), 3.53 (t, J = 6.7 Hz, 2H), 3.46 (t, J = 6.6 Hz, 2H), 3.39 (t, J = 5.1 Hz, 2H), 1.82 – 1.73 (m, 2H), 1.60 (p, J = 6.8 Hz, 2H), 1.52 – 1.31 (m, 6H). ^{13}C NMR (100 MHz, Chloroform-*d*) δ 71.24, 70.74, 70.72, 70.66, 70.11, 70.04, 50.70, 45.07, 32.56, 29.46, 26.71, 25.43. APCI-MS: m/z calcd. for 293.15, measured for 265.88 $[M+H-N_2]^+$.

Synthesis of 2-(2-(2-((6-chlorohexyl)oxy)ethoxy)ethoxy)ethan-1-aminium chloride After two vacuum/H2 cycles to replace air inside the reaction tube with hydrogen, the mixture of 1b (35 mg, 0.12 mmol),10% Pd/C (3.5 mg 10 wt % of 1b) and 2-3 drops of 1N HCl in MeOH (0.6 mL) was stirred at r.t. under hydrogen balloon

for 2 h. The reaction mixture was filtered using celite filter to afford compound 1c as quantitative yield. ^{1}H NMR (400 MHz, DMSO-d_6) δ 8.39 – 8.02 (m, 3H), 3.59 (dq, J = 6.7, 3.6, 3.1 Hz, 4H), 3.55 – 3.41 (m, 8H), 3.35 (d, J = 2.9 Hz, 2H), 2.90 (t, J = 5.3 Hz, 2H), 1.73 – 1.63 (m, 2H), 1.46 (dd, J = 9.9, 4.7 Hz, 2H), 1.40 – 1.24 (m, 4H). ^{13}C NMR (100 MHz, DMSO-d_6) δ 70.62, 70.31 – 69.74 (m), 67.01, 45.82, 38.82, 32.45, 29.47, 26.54, 25.35. APCI-MS: m/z calcd for 267.16, measured for 267.95 $[M+H]^+$.

Synthesis of UL1 To synthesize aryl-azide-PEG2 HaloTag Ligand (UL1), 4-azidobezoic acid 5 (83 mg, 0.50 mmoles) was taken in 10 ml round bottom flask with HATU (214 mg, 0.56 mmoles), DIPEA (131.3 mg, 1.02 mmoles) and dissolved in 2 ml of DCM. The reaction mixture was stirred at room temperature for 30 minutes or until the color turned brown, then 2-(2-((6-chlorohexyl)oxy)ethoxy)ethanamine (138 mg, 0.61 mmoles)[9] dissolved in 1 ml DCM was added dropwise to reaction mixture and reaction was stirred for additional 3 hours at room temperature. After completion of the reaction, solvent was removed under reduced pressure to obtain a colorless oil which was purified by column chromatography over silica gel (Eluent: 40 % EtOAc in Hexane) to furnish the pure 6 (168 mg, 90%) as a colorless oil. 1H NMR (400 MHz, CDCl3) δ 7.80 (d, J = 8.7 Hz, 2H), 7.06 (d, J = 8.7 Hz, 2H), 6.67 (br s, 1H), 3.70 – 3.63 (m, 6H), 3.62 – 3.57 (m, 2H), 3.52 (t, J = 6.7 Hz, 2H), 3.46 (t, J = 6.7 Hz, 2H), 1.78 – 1.70 (m, 2H), 1.61-1.54 (m, 2H), 1.46-1.32 (m, 4H). 13C NMR (100 MHz, CDCl3) δ 166.55, 143.35, 131.19, 128.93, 119.04, 77.16, 71.39, 70.35, 70.12, 69.81, 45.12, 39.84, 38.73, 32.59, 29.55, 26.78, 25.50, HR-ESI-MS: m/z calcd. for 368.1615, measured for 368.1614.

Synthesis of UL2 To synthesize aryl-azide-PEG3 HaloTag Ligand (UL2), 4-azidobezoic acid 5 (45 mg, 0.28 mmoles) was taken in 10 ml round bottom flask with HATU (105 mg, 0.28 mmoles), DIPEA (64 mg, 0.58 mmoles) and dissolved in 2 ml of DCM. The reaction mixture was stirred at room temperature for 30 minutes or until the color turned brown, then 2-(2-(2-((6-chlorohexyl)oxy)ethoxy)ethoxy)ethanamine (68mg, 0.25 mmoles)[10] dissolved in 1 ml DCM was added dropwise to reaction mixture and reaction was stirred for additional 3 hours at room temperature. After completion of the reaction, solvent was removed under reduced pressure to obtain a colorless oil which was purified by column chromatography over silica gel (Eluent: 60 % EtOAc in Hexane) to furnish the pure 14 (98 mg, 95%) as a colorless oil. , 1H NMR (400 MHz, Acetone) δ 7.95 (d, J = 8.8 Hz, 2H), 7.77 (s, 1H), 7.16 (d, J = 8.8 Hz, 2H), 3.66 – 3.54 (m, 12H), 3.54 – 3.49 (m, 2H), 3.41 (t, J = 6.5 Hz, 2H), 1.75 (m, 2H), 1.57 – 1.49 (m, 2H), 1.47 – 1.32 (m, 4H). 13C NMR (101 MHz, Acetone) δ 166.67, 143.74, 132.45, 129.94, 119.66, 71.56, 71.10, 71.09, 70.92, 70.76, 70.54, 45.75, 40.50, 38.72, 33.36, 30.28, 27.36, 26.15, HR-ESI-MS: m/z calcd. for 412.1877, measured for 412.1877.

Synthesis of UL3 To synthesize N-(2-(2-(2-((6-chlorohexyl)oxy)ethoxy)ethoxy)ethyl)-3-(3-methyl-3H-diazirin-3-yl)propenamide (UL3), a solution of 3-(3-methyl-3H-diazirin-3-yl)propanoic acid (13.4 mg, 0.05 mmol)[11], EDC-HCl (10.5 mg, 0.055 mmol), TEA (7 uL, 0.05 mmol) and DMAP (6.1 mg, 0.05 mmol) in dry THF (0.3 mL) at r.t. was added a solution of 2-(2-(2-((6-chlorohexyl)oxy)ethoxy)ethoxy)ethan-1-aminium chloride (1c, 15.2 mg, 0.05 mmol) in THF (0.2 mL). The mixture was stirred for 2 h, and was concentrated in vacuo. The crude material was purified by flash chromatography to yield 3a (7 mg, yield : 37%). ^{1}H NMR (600 MHz, Chloroform-d) δ 6.30 (s, 1H), 3.64 – 3.51 (m, 12H), 3.48 – 3.42 (m, 4H), 2.02 – 1.98 (m, 2H), 1.79 – 1.72 (m, 4H), 1.60 (p, J = 6.9 Hz, 2H), 1.45 (dt, J = 14.8, 6.8 Hz, 2H), 1.37 (q, J = 8.0 Hz, 2H), 1.02 (s, 3H). ^{13}C NMR (150 MHz, Chloroform-d) δ 171.36, 71.26, 70.50, 70.47, 70.20, 70.05, 69.82, 45.01, 39.27, 32.50, 30.57, 30.06, 29.38, 26.65, 25.46, 25.37, 19.86. HRMS-DART: m/z calcd for 377.2081, measured for 378.2154 $[M+H]^+$.

Synthesis of UL4 To synthesize N-(2-(2-(2-((6-chlorohexyl)oxy)ethoxy)ethoxy)ethyl)-4-(3-(trifluoromethyl)-3H-diazirin-3-yl)benzamide (UL4), a solution of 4-(3-(trifluoromethyl)-3H-diazirin-3-yl)benzoic acid (11.5 mg, 0.05 mmol), EDC-HCl (10.5 mg, 0.055 mmol), TEA (7 uL, 0.05 mmol) and DMAP

(6.1 mg, 0.05 mmol) in dry THF (0.3 mL) at r.t. was added a solution of 2-(2-(2-((6-chlorohexyl)oxy)ethoxy)ethoxy)ethan-1-aminium chloride (1c, 15.2 mg, 0.05 mmol) in THF (0.2 mL). The mixture was stirred for 2 h, and was concentrated in vacuo. The crude material was purified by flash chromatography to yield 3a (14 mg, yield : 60%). ^{1}H NMR (600 MHz, Chloroform-*d*) δ 7.89 – 7.82 (m, 2H), 7.23 (d, *J* = 8.2 Hz, 2H), 7.09 (d, *J* = 5.4 Hz, 1H), 3.68 – 3.61 (m, 10H), 3.54 (dd, *J* = 5.8, 3.7 Hz, 2H), 3.51 (t, *J* = 6.7 Hz, 2H), 3.40 (t, *J* = 6.7 Hz, 2H), 1.78 – 1.71 (m, 2H), 1.54 (dt, *J* = 14.7, 6.9 Hz, 2H), 1.45 – 1.38 (m, 2H), 1.32 (p, *J* = 7.6, 7.1 Hz, 2H). ^{13}C NMR (100 MHz, Chloroform-*d*) δ 166.19, 135.70, 132.10, 127.62, 126.46, 126.45, 121.93 (d, *J* = 274.8 Hz), 71.27, 70.54, 70.52, 70.25, 70.05, 69.76, 45.00, 39.91, 32.50, 29.35, 28.36 (d, *J* = 40.5 Hz), 26.64, 25.36. HRMS-DART: m/z calcd for 479.1799, measured for 480.1871 $[M+H]^{+}$.

Synthesis of VL1 3-(6-azido-1,3-dioxo-1H-benzo[de]isoquinolin-2(3H)-yl)propanoic acid(180 mg, 0.582 mmol) was taken in a 10 ml round bottom flask and 1 ml DMF with HATU (254 mg, 0.67 mmol) and DIPEA (240 ul, 1.3 mmol) and stirred at room temperature for 30 min until reaction mixture turned to yellowish precipitate then NH2-PEG2-HTL was added along with 1 ml DMF and reaction mixture turns uniform liquid for a moment and again becomes precipitate kept on stirring for another 1 hour and then work up was performed using EtOAc and water, organic layer dried over Na2SO4 and purification was performed using silica gel Colum (1% MeOH in CHCl3) to afford 180 mg of yellow powder(60%).1H NMR (400 MHz, CDCl3) δ 8.64 (dd, J = 7.3, 1.1 Hz, 1H), 8.59 (d, J = 8.0 Hz, 1H), 8.45 (dd, J = 8.4, 1.0 Hz, 1H), 7.75 (dd, J = 8.4, 7.4 Hz, 1H), 7.48 (d, J = 8.0 Hz, 1H), 6.28 (s, 1H), 4.54 – 4.44 (m, 2H), 3.62 – 3.38 (m, 11H), 2.71 – 2.61 (m, 2H), 1.81 – 1.69 (m, 2H), 1.58 (d, J = 7.4 Hz, 2H), 1.48 – 1.31 (m, 4H). 13C NMR (100 MHz, CDCl3) δ 170.34, 163.97, 163.53, 143.73, 132.44, 131.95, 129.28, 129.04, 126.99, 124.46, 122.55, 118.81, 114.80, 77.16, 71.37, 70.35, 70.10, 69.90, 45.15, 39.32, 37.02, 34.86, 32.61, 29.55, 26.79, 25.52.

Synthesis of VL2 3-(6-azido-1,3-dioxo-1H-benzo[de]isoquinolin-2(3H)-yl)propanoic acid(116 mg, 0.37 mmol) was taken in a 10 ml round bottom flask and 1 ml DMF with HATU (160 mg, 0.42 mmol) and DIPEA (255 ul,) and stirred at room temperature for 30 min until reaction mixture turned to yellowish precipitate then NH2-PEG3-HTL was added along with 1 ml DMF and reaction mixture turns uniform liquid for a moment and again becomes precipitate kept on stirring for another 1 hour and then work up was performed using EtOAc and water, organic layer dried over Na2SO4 and purification was performed using silica gel Colum (1% MeOH in CHCl3) to afford yellow powder(55%). 1H NMR (400 MHz, CDCl3) δ 8.64 (dd, J = 7.3, 1.0 Hz, 1H), 8.59 (d, J = 8.0 Hz, 1H), 8.45 (dd, J = 8.4, 1.0 Hz, 1H), 7.75 (dd, J = 8.3, 7.4 Hz, 1H), 7.48 (d, J = 8.0 Hz, 1H), 6.40 (s, 1H), 4.53 – 4.46 (m, 2H), 3.65 – 3.53 (m, 9H), 3.50 (t, J = 6.7 Hz, 2H), 3.45 (dt, J = 11.0, 6.0 Hz, 4H), 2.70 – 2.61 (m, 2H), 1.79 – 1.69 (m, 2H), 1.56 (dd, J = 14.5, 7.0 Hz, 2H), 1.37 (ddd, J = 23.3, 15.9, 8.7 Hz, 4H).13C NMR (101 MHz, CDCl3) δ 170.42 , 163.96 , 163.51, 143.70, 132.42, 131.93, 129.28, 129.01, 126.98, 124.46, 122.58, 118.85, 114.79, 71.36, 70.63 (d, J = 4.7 Hz), 70.26 (d, J = 15.8 Hz), 69.95, 45.14, 39.38, 37.04, 34.81, 32.63, 29.52, 26.78, 25.51.

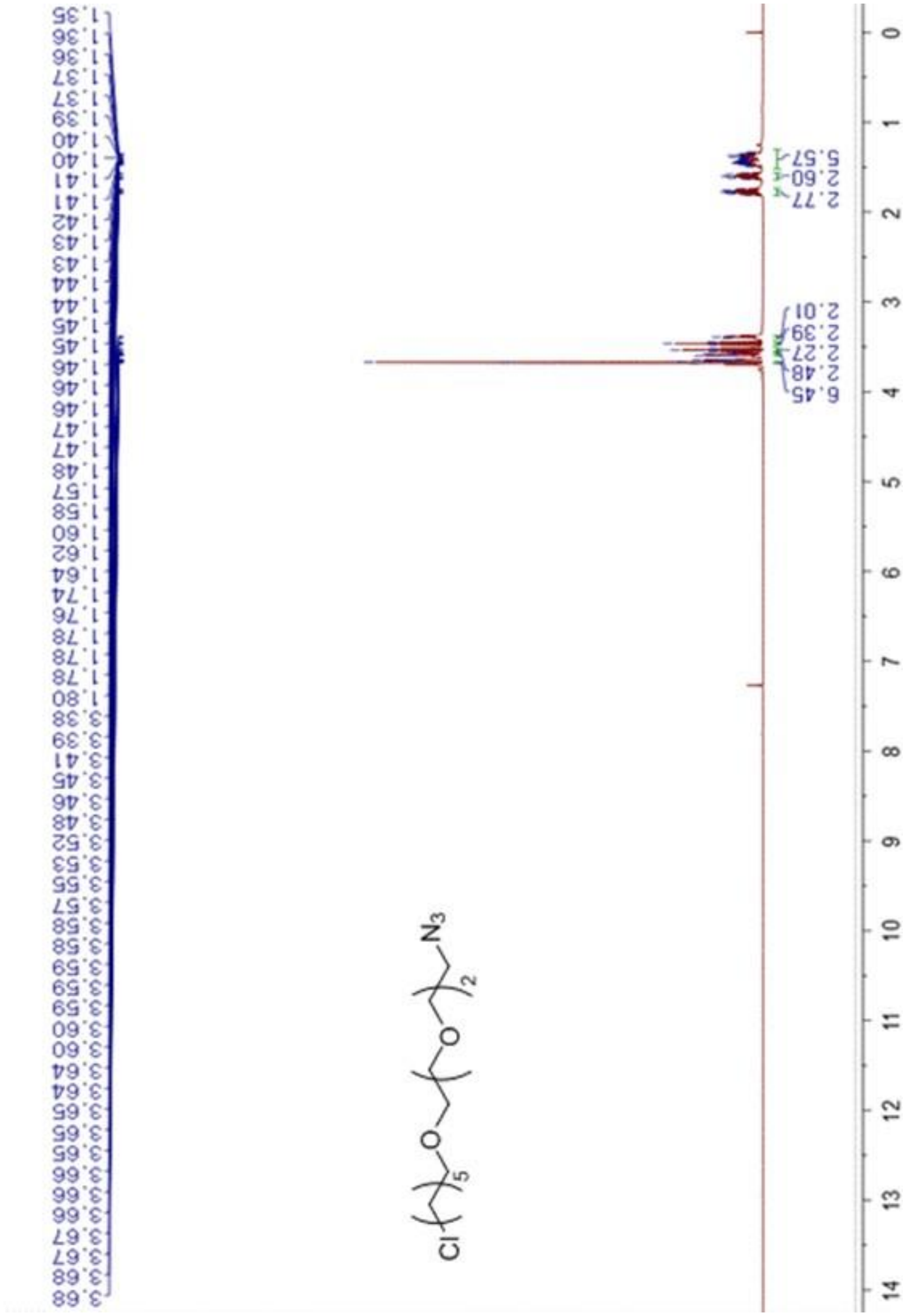

^{1}H NMR spectrum (400 MHz, Chloroform-d) of 1-(2-(2-(2-azidoethoxy)ethoxy)ethoxy)-6-chlorohexane

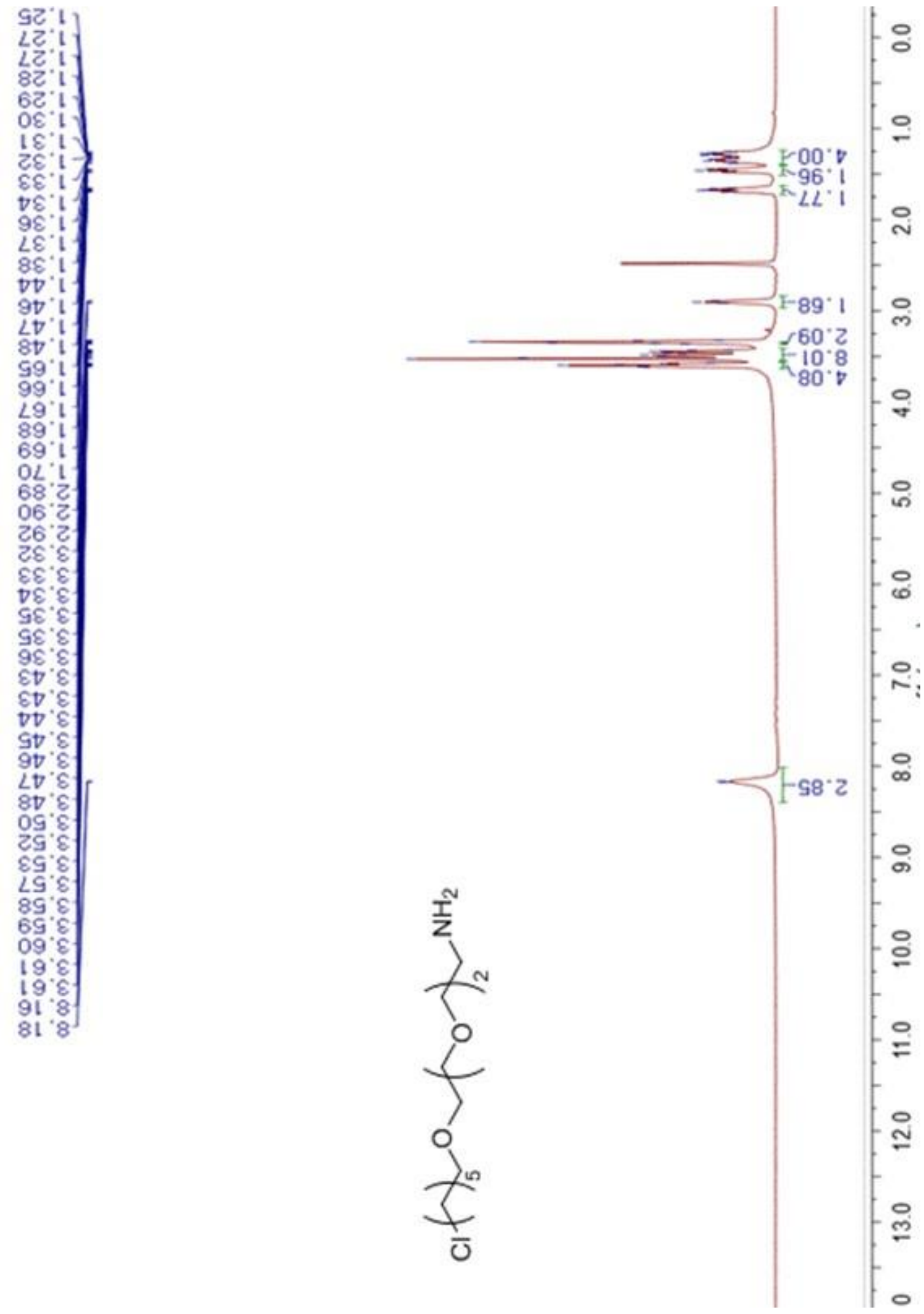

^{1}H NMR spectrum (400 MHz, Chloroform-d) of 2-(2-(2-((6-chlorohexyl)oxy)ethoxy) ethoxy)ethan-1-aminium chloride

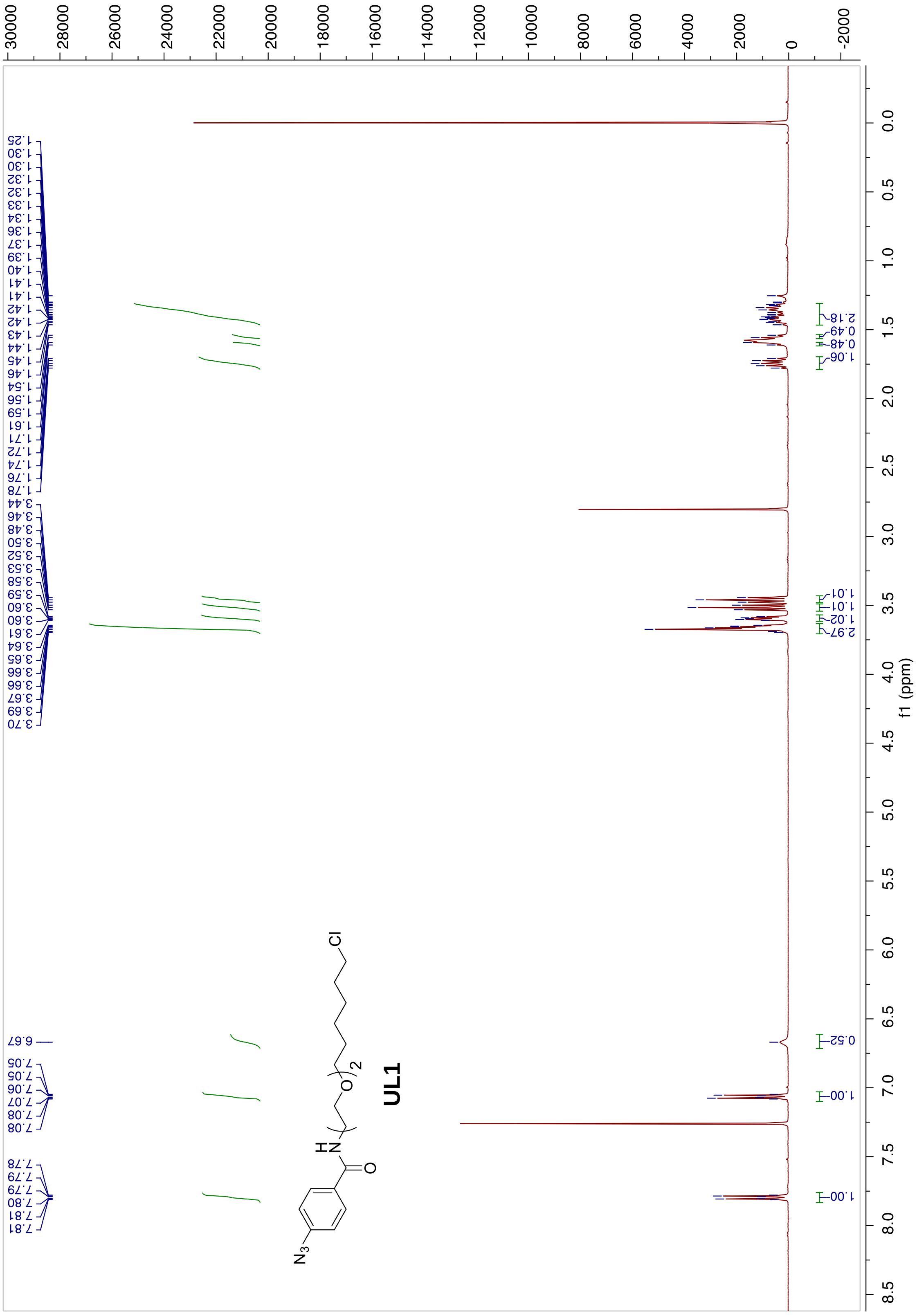

^{1}H NMR spectrum (400 MHz, $CDCl_3$) of **UL1**

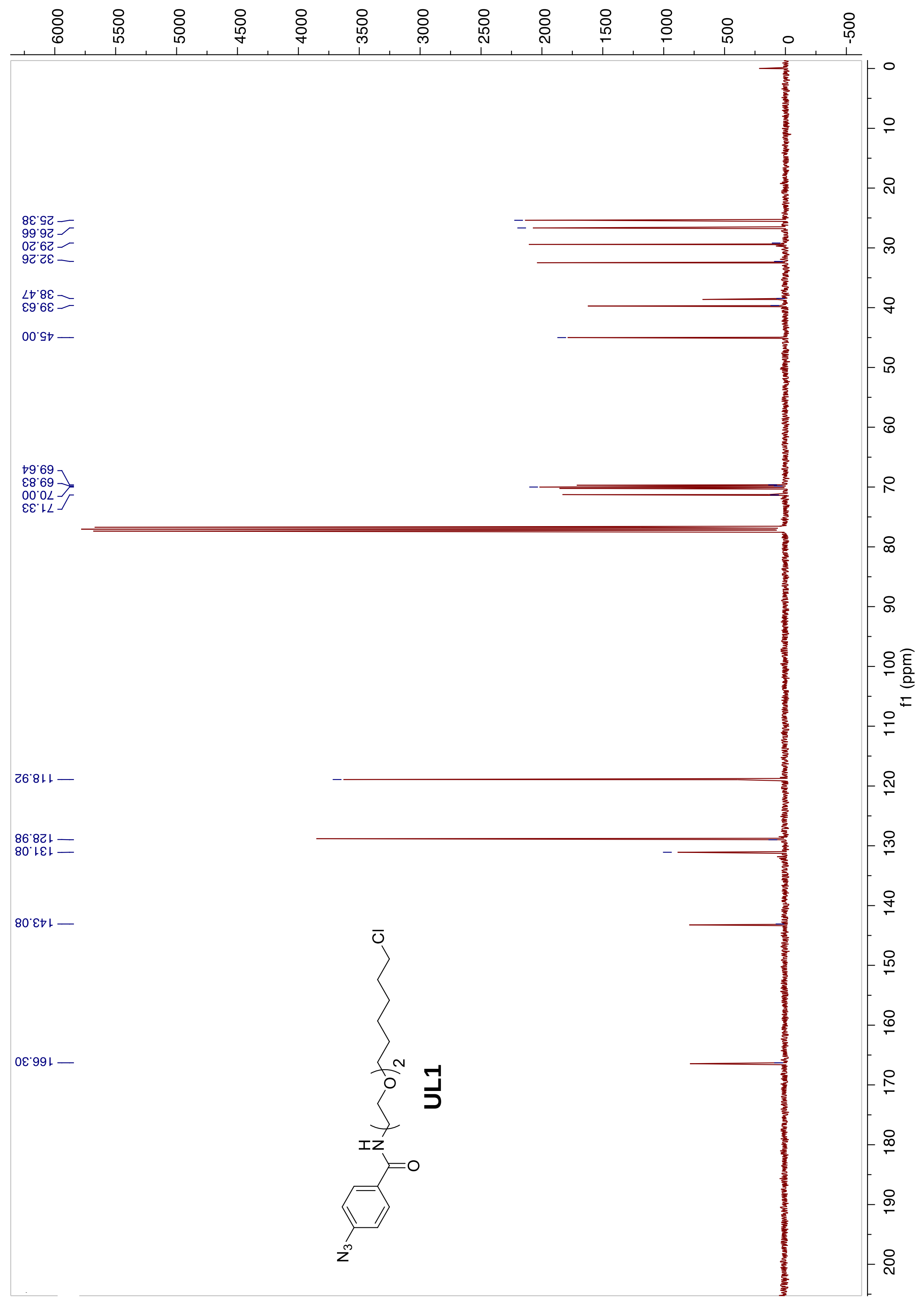

^{13}C NMR spectrum (100 MHz, $CDCl_3$) of **UL1**

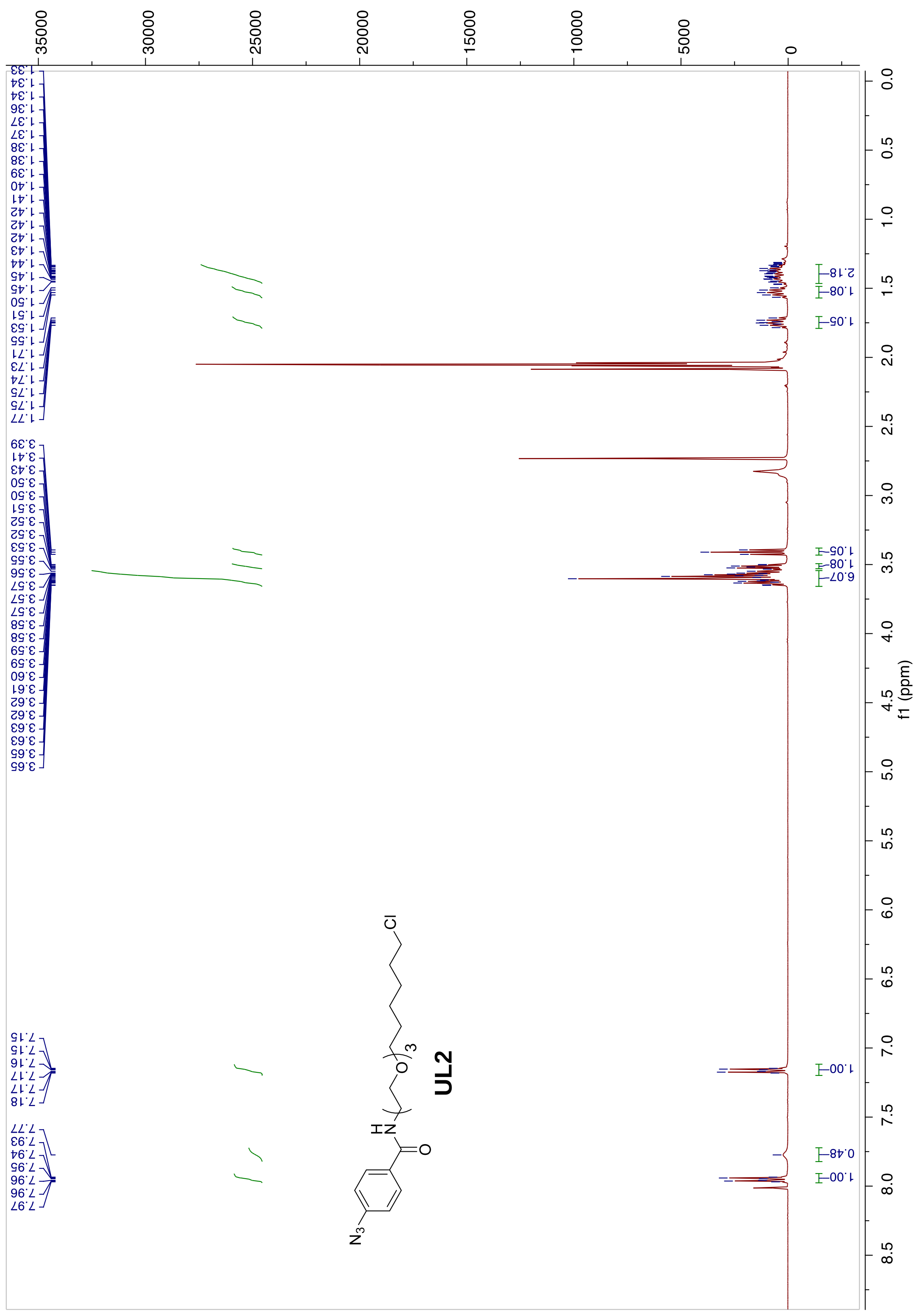

^{1}H NMR spectrum (400 MHz, Acetone) of **UL2**

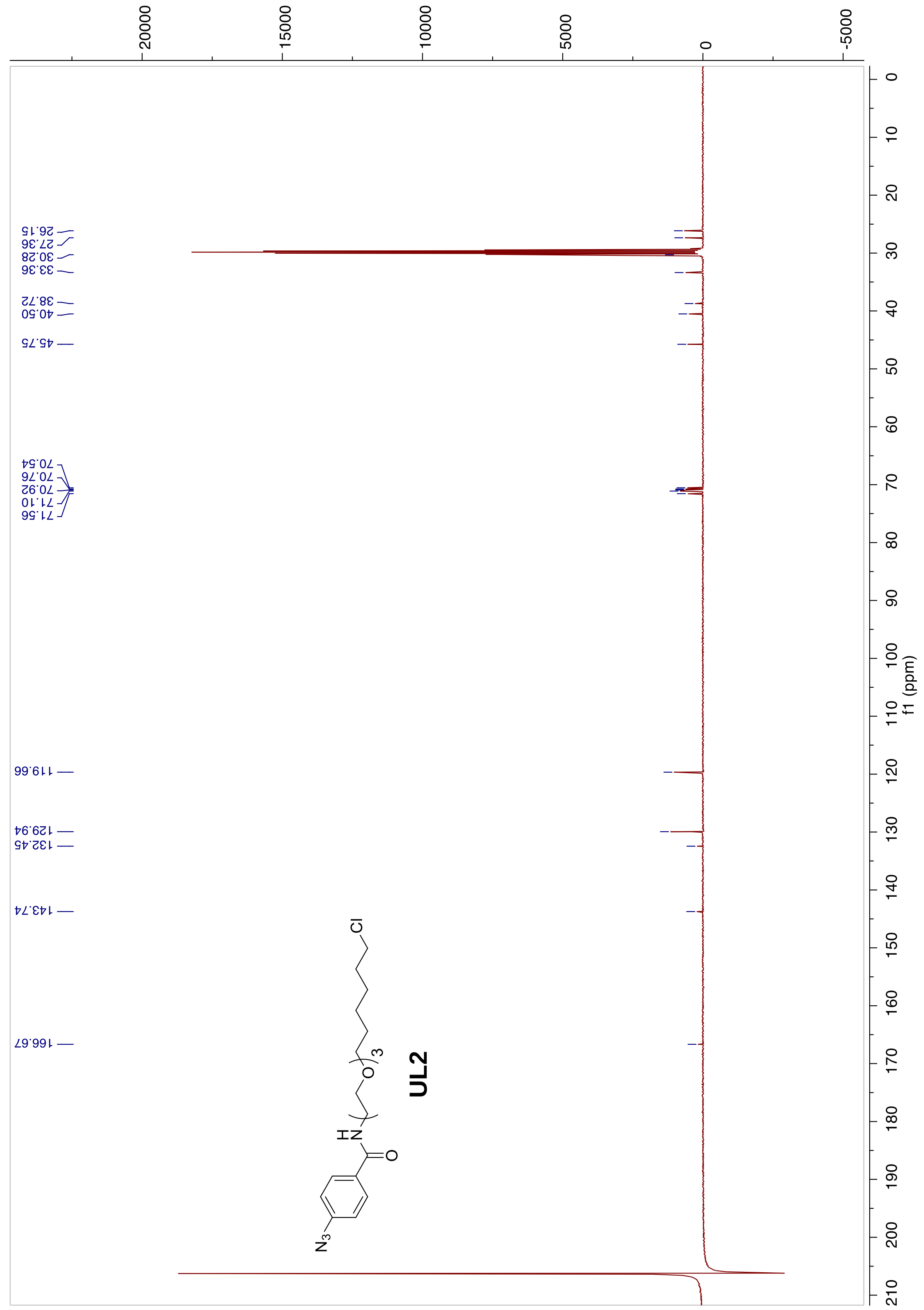

: ^{13}C NMR spectrum (100 MHz, Acetone) of **UL2**

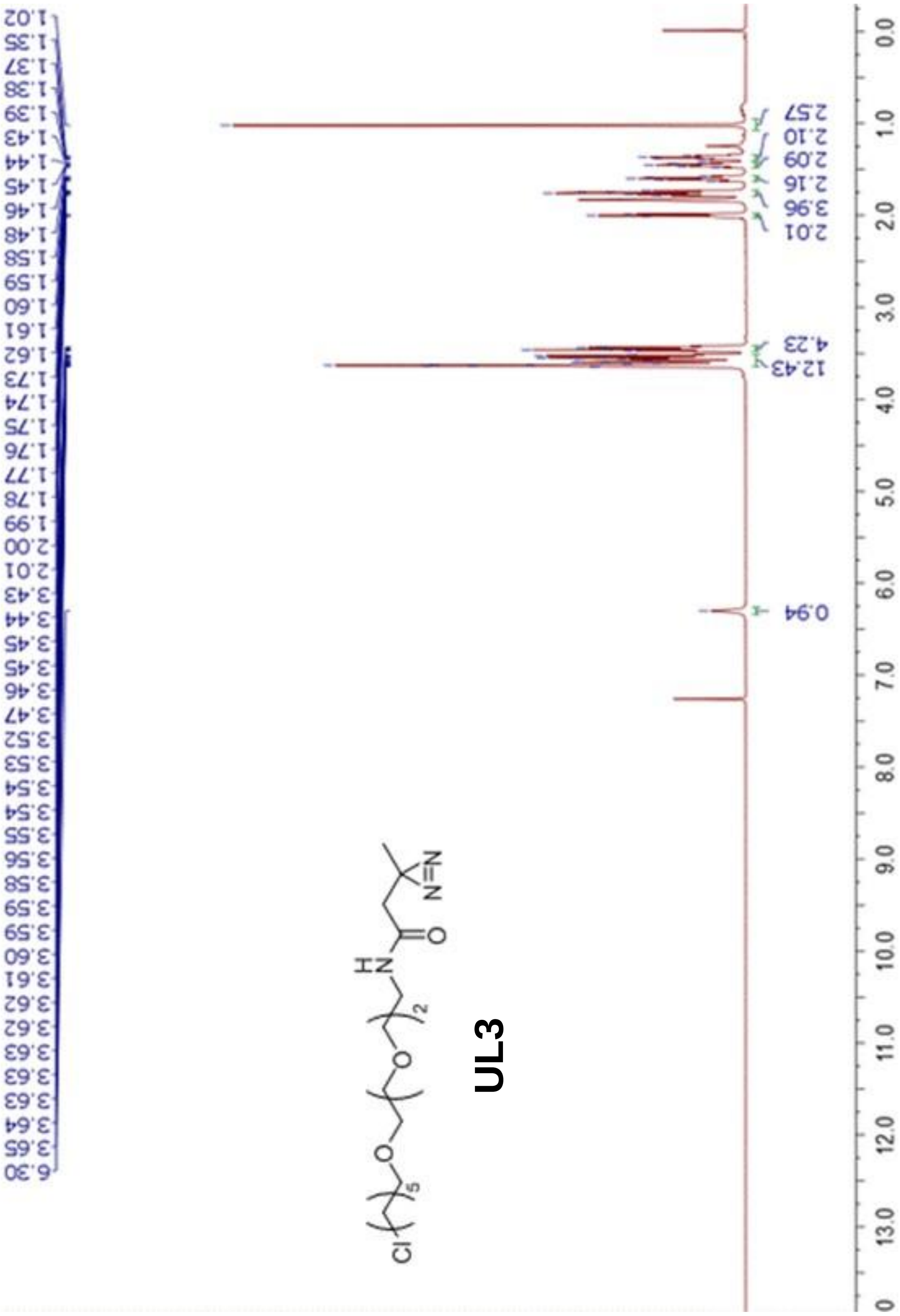

^{1}H NMR spectrum (400 MHz, Chloroform-d) of **UL3**

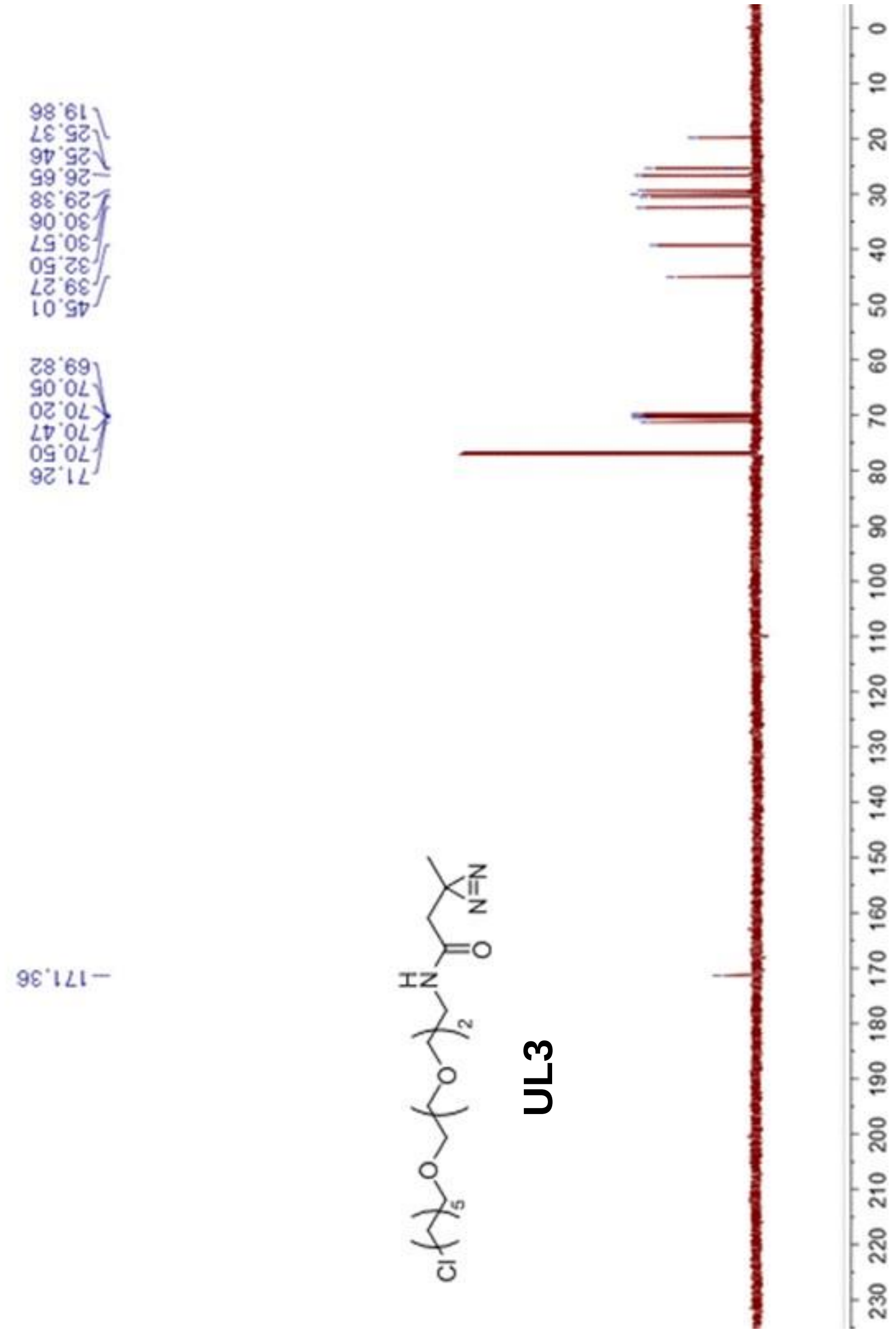

^{13}C NMR spectrum (100 MHz, Chloroform-d) of **UL3**

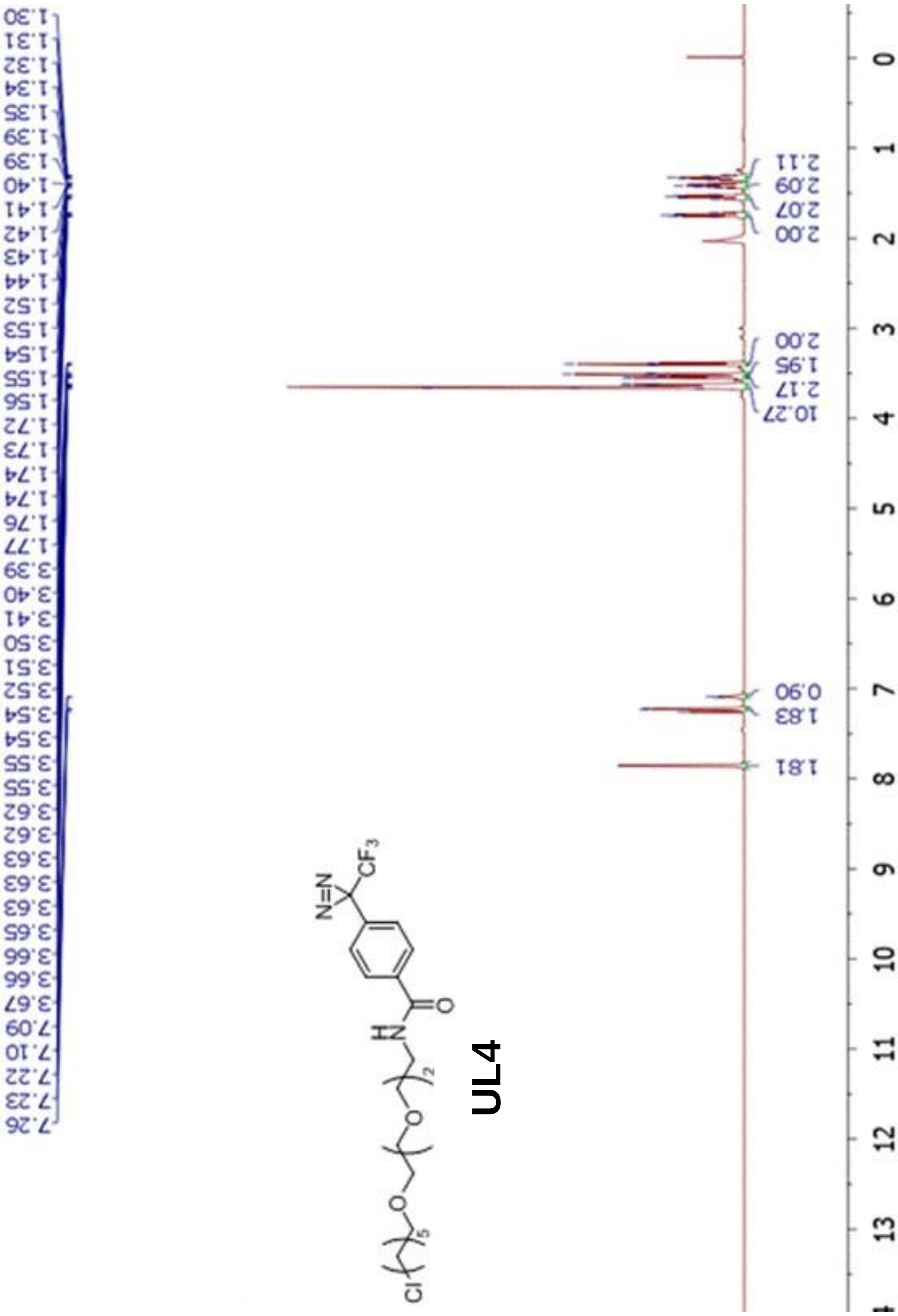

^{1}H NMR spectrum (400 MHz, Chloroform-d) of **UL4**

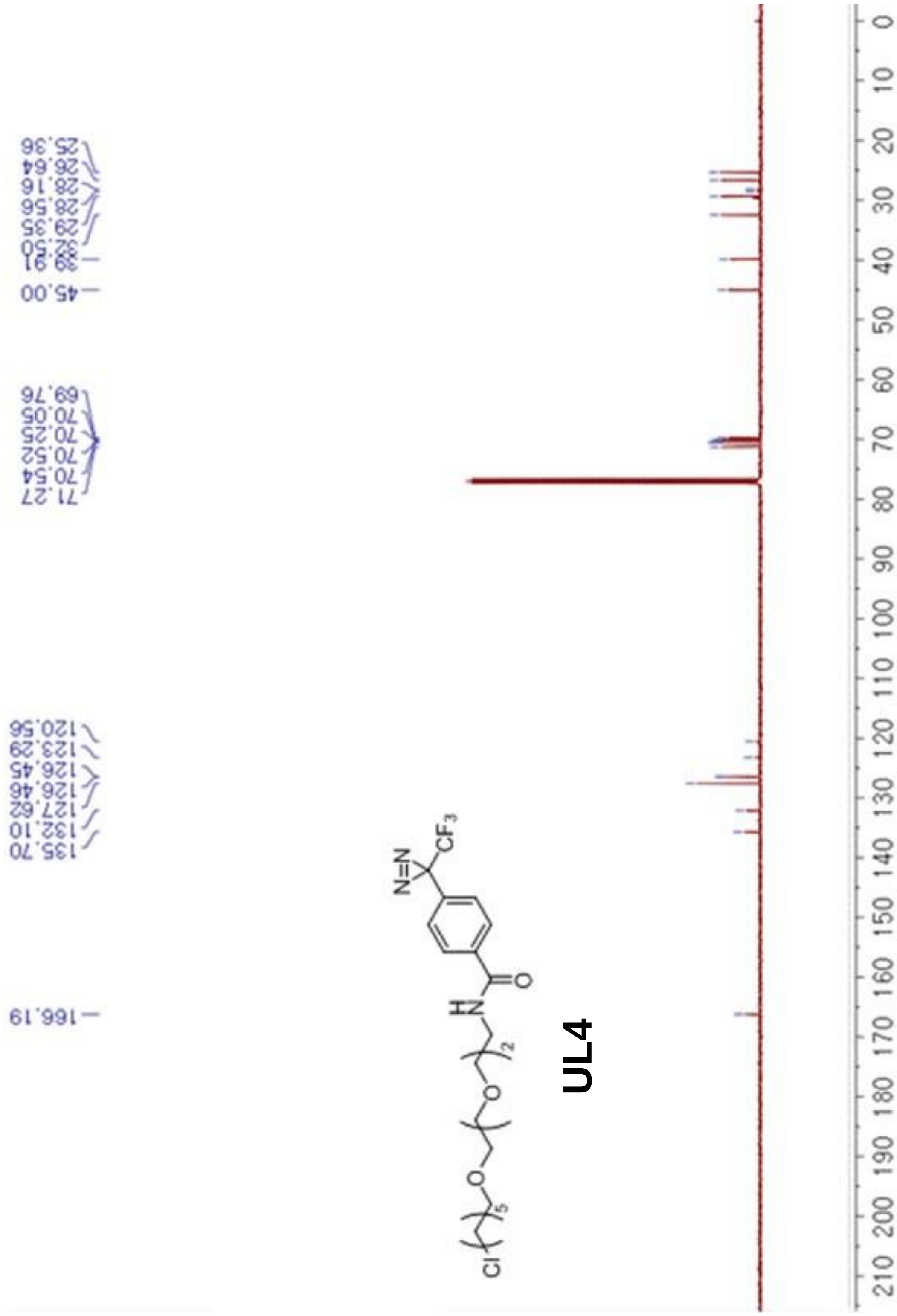

^{13}C NMR spectrum (100 MHz, Chloroform-d) of U**L4**

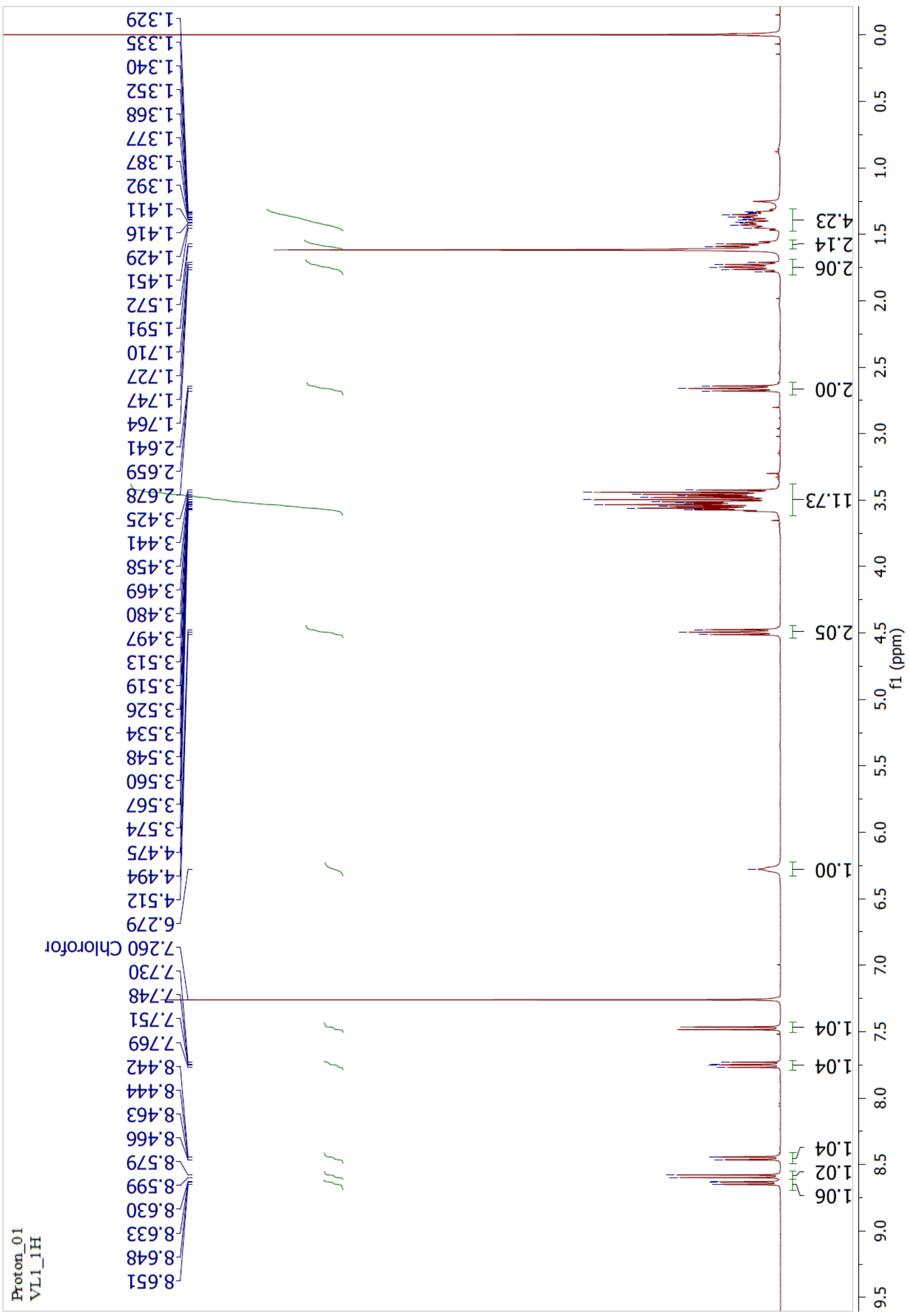

^{1}H NMR spectrum (400 MHz, Chloroform-d) of **VL1**

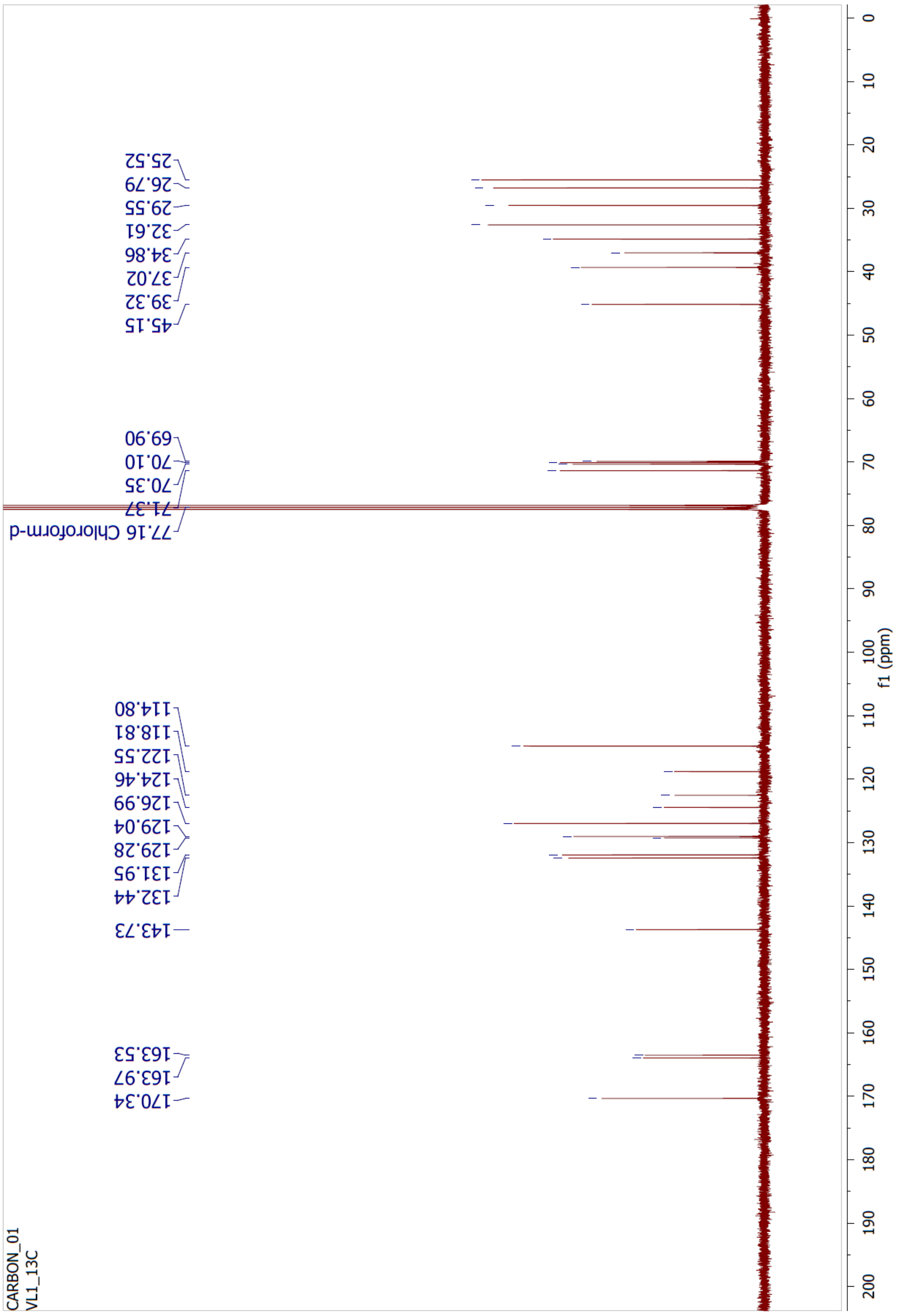

^{13}C NMR spectrum (100 MHz, Chloroform-d) of **VL1**

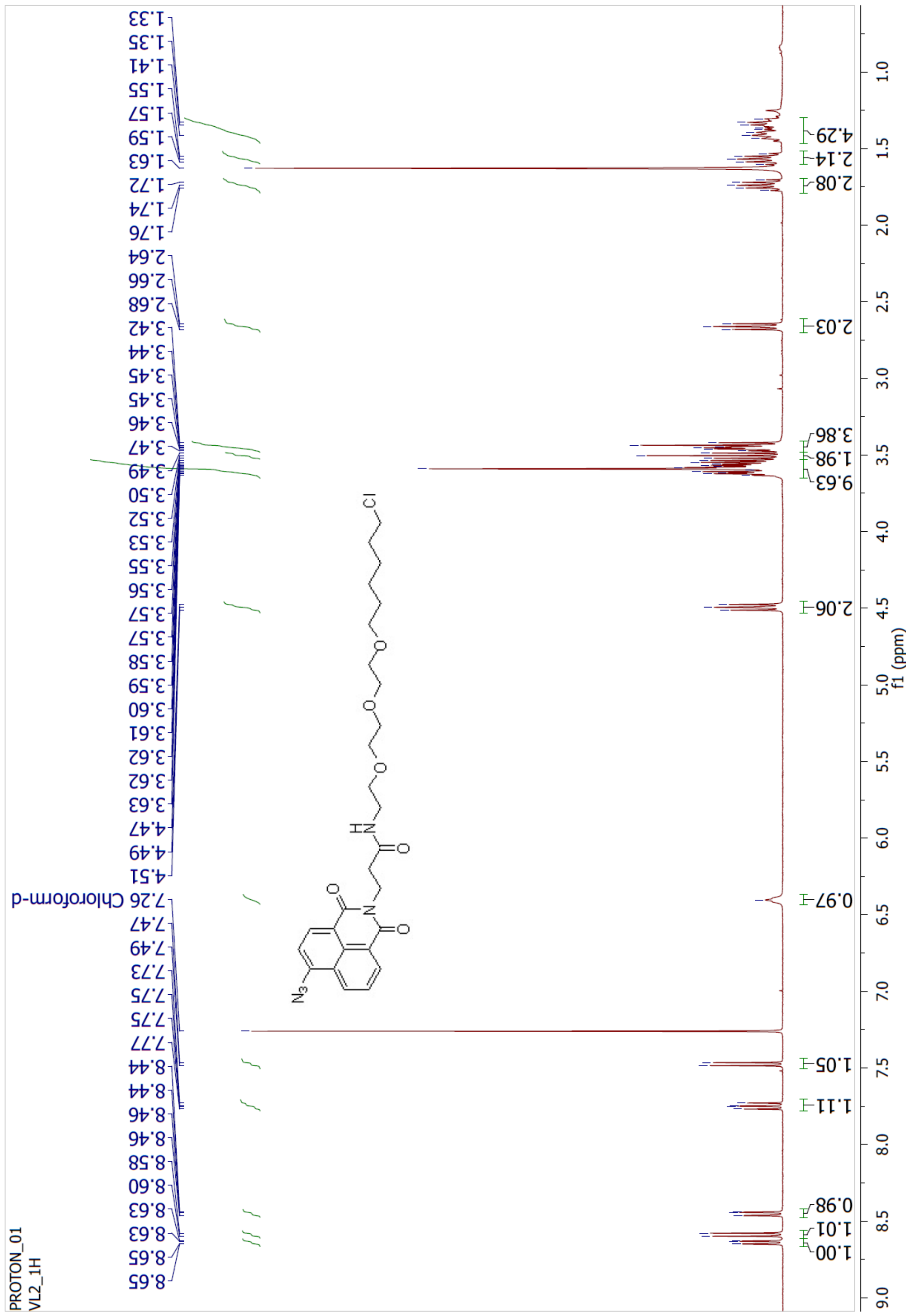

^{1}H NMR spectrum (400 MHz, Chloroform-d) of **VL2**

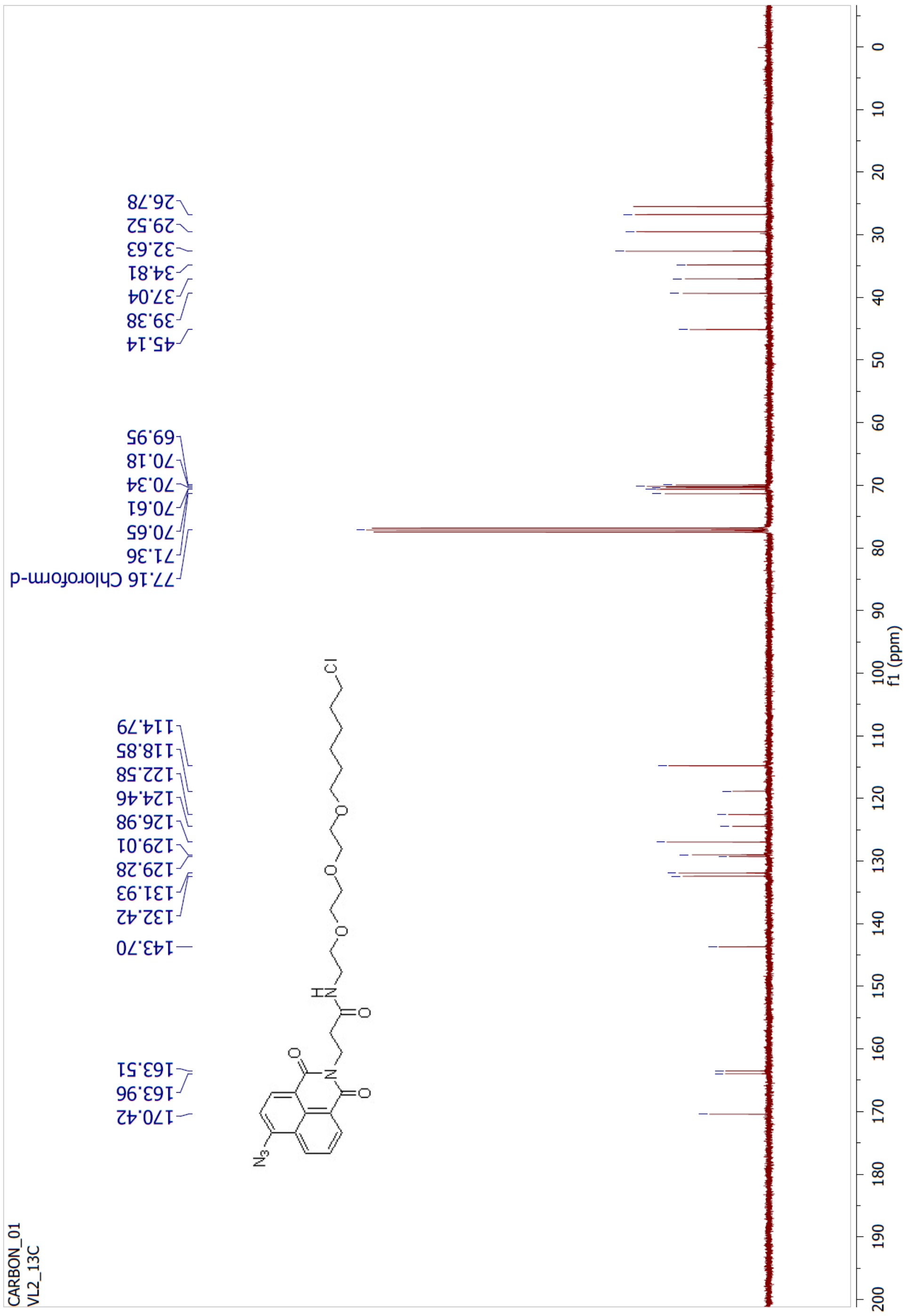

^{13}C NMR spectrum (100 MHz, Chloroform-d) of **VL2**

Supplementary references

1. Lee, S. Y.; Kang, M. G.; Shin, S.; Kwak, C.; Kwon, T.; Seo, J. K.; Kim, J. S.; Rhee, H. W., Architecture Mapping of the Inner Mitochondrial Membrane Proteome by Chemical Tools in Live Cells. *J Am Chem Soc* **2017,** *139* (10), 3651-3662.

2. Otwinowski, Z.; Minor, W., [20] Processing of X-ray diffraction data collected in oscillation mode. In *Methods in Enzymology*, Academic Press: 1997; Vol. 276, pp 307-326.

3. Adams, P. D.; Afonine, P. V.; Bunkoczi, G.; Chen, V. B.; Davis, I. W.; Echols, N.; Headd, J. J.; Hung, L.-W.; Kapral, G. J.; Grosse-Kunstleve, R. W.; McCoy, A. J.; Moriarty, N. W.; Oeffner, R.; Read, R. J.; Richardson, D. C.; Richardson, J. S.; Terwilliger, T. C.; Zwart, P. H., PHENIX: a comprehensive Python-based system for macromolecular structure solution. *Acta Crystallographica Section D* **2010,** *66* (2), 213-221.

4. Emsley, P.; Lohkamp, B.; Scott, W. G.; Cowtan, K., Features and development of Coot. *Acta Crystallogr D Biol Crystallogr* **2010,** *66* (Pt 4), 486-501.

5. Kim, S.; Park, D.; Kim, J.; Kim, D.; Kim, H.; Mori, T.; Jung, H.; Lee, D.; Hong, S.; Jeon, J.; Tabuchi, K.; Cheong, E.; Kim, J.; Um, J. W.; Ko, J., Npas4 regulates IQSEC3 expression in hippocampal somatostatin interneurons to mediate anxiety-like behavior. *Cell Rep.* **2021,** *36* (3), 109417.

6. Nesvizhskii, A. I.; Keller, A.; Kolker, E.; Aebersold, R., A Statistical Model for Identifying Proteins by Tandem Mass Spectrometry. *Analytical Chemistry* **2003,** *75* (17), 4646-4658.

7. Chang, C.; Xu, K.; Guo, C.; Wang, J.; Yan, Q.; Zhang, J.; He, F.; Zhu, Y., PANDA-view: an easy-to-use tool for statistical analysis and visualization of quantitative proteomics data. *Bioinformatics* **2018,** *34* (20), 3594-3596.

8. Bollong, M. J.; Pietila, M.; Pearson, A. D.; Sarkar, T. R.; Ahmad, I.; Soundararajan, R.; Lyssiotis, C. A.; Mani, S. A.; Schultz, P. G.; Lairson, L. L., A vimentin binding small molecule leads to mitotic disruption in mesenchymal cancers. *Proc Natl Acad Sci U S A* **2017,** *114* (46), E9903-E9912.

9. Neklesa, T. K.; Tae, H. S.; Schneekloth, A. R.; Stulberg, M. J.; Corson, T. W.; Sundberg, T. B.; Raina, K.; Holley, S. A.; Crews, C. M., Small-molecule hydrophobic tagging-induced degradation of HaloTag fusion proteins. *Nat Chem Biol* **2011,** *7* (8), 538-43.

10. Benink, H.; McDougall, M.; Klaubert, D.; Los, G., Direct pH measurements by using subcellular targeting of 5(and 6-) carboxyseminaphthorhodafluor in mammalian cells. *Biotechniques* **2009,** *47* (3), 769-74.

11. Wang, L.; Tachrim, Z. P.; Kurokawa, N.; Ohashi, F.; Sakihama, Y.; Hashidoko, Y.; Hashimoto, M., Base-Mediated One-Pot Synthesis of Aliphatic Diazirines for Photoaffinity Labeling. *Molecules* **2017,** *22* (8).
